# 2-(Methoxycarbonyl)thiophen-3-yl-diazonium Salts: Efficient Precursors for the Formation of C–C Bonds in Thiophene-Containing Heterocyclic Systems

**DOI:** 10.3390/molecules30183758

**Published:** 2025-09-16

**Authors:** Yurii V. Ostapiuk, Oksana V. Barabash, Mary Y. Ostapiuk, Mykola Kravets, Andreas Schmidt, Mykola D. Obushak

**Affiliations:** 1 Department of Organic Chemistry, Ivan Franko National University of Lviv, Kyryla i Mefodiya Str. 6, 79005 Lviv, Ukraineyurii.ostapiuk@lnu.edu.ua (M.Y.O.); mykola.obushak@lnu.edu.ua (M.D.O.); 2 Institute of Physical Chemistry of the Polish Academy of Sciences, Kasprzaka 44/52, 01-224 Warsaw, Poland; 3 Institute of Organic Chemistry, Clausthal University of Technology, Leibnizstrasse 6, D-38678 Clausthal-Zellerfeld, Germany

**Keywords:** 3-thienyldiazonium salts, Meerwein reaction, C–C bond formation, thienopyranone, thiazole, selenazole, bithiophene

## Abstract

A first application of 2-(methoxycarbonyl)thiophen-3-diazonium salts in the halothienylation of α,β-unsaturated functionalized compounds under Meerwein reaction conditions is presented. This process provides thiophene-containing building blocks that can then undergo cyclization reactions with bisnucleophiles to synthesize thiophene-3-yl-containing heterocyclic systems. In this way, substituted 2,3′-bithiophene compounds and thiophene-3-yl derivatives of 2-aminothiazole, 2-aminoselenazole, thiazolidine, and selenazolidine can be efficiently prepared.

## 1. Introduction

Substituted thiophene compounds are an important class of heterocyclic compounds due to their versatile applications. The thiophene core is an important scaffold for a variety of therapeutics and drugs covering the spectrum from antitumor, antibacterial, anti-inflammatory, antifungal, antihelminthic, antihypertensive, antiviral, antipsychotic agents to local anesthetics and SIRT2 inhibitors for the treatment of myocardial fibrosis [1,2,3,4,5,6]. Concerning materials chemistry, thiophene derivatives are also used as chemosensors [7], photovoltaic materials [8], dyes, and fluorophores [9,10,11]. It is striking that the derivatives substituted at positions 2 and 5 of the thiophene ring are the best studied which is due to the ease with which thiophene compounds can be modified at these positions. The introduction of substituents and the formation of C–C bonds at positions 3 and 4 can be difficult, especially if positions 2 and 5 of the thiophene ring are to remain unsubstituted. Nevertheless, the further development of the chemistry of 3-substituted thiophene compounds opens up new and interesting perspectives, and they are playing an increasingly important role in pharmaceutical research [5,6,12,13,14].

**Figure 1:**
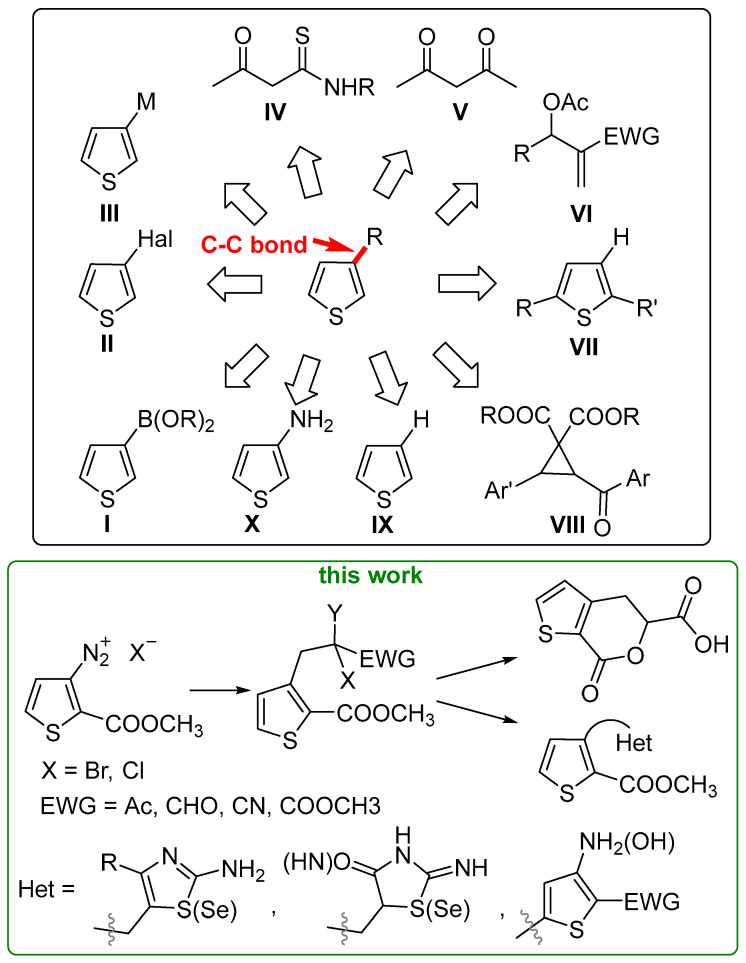
Methods for C–C bond formations in position 3 of thiophenes (above). Synthetic potential of the title compounds (below).

Thiophenes with a C–C bond in the 3-position have mostly been synthesized by modifying the existing thiophene core, often starting from 3-thienylboronic acids **I** for Suzuki-Miyaura cross-couplings [15,16,17,18] or 3-halothiophene compounds **II** for Sonogashira reactions or Suzuki couplings with boronic acids as coupling partners (Figure 1) [12,19]. In addition, 3-halothiophenes serve as starting materials for the preparation of organolithium compounds and organostannanes as intermediates for the introduction of substituents into the 3-position of the thiophene ring **III** [20,21,22]. 3-Halothiophene can be obtained by direct halogenation of thiophenes with an electron-withdrawing group in the 5-position or with substituted 2- and 5-positions [19,23,24]. However, the use of 3-aminothiophenes in the Sandmeyer reaction is often much more practical [13,14]. In some cases, the use of suitable acyclic precursors allows the construction of derivatives with a carbon substituent in the 3-position of a thiophene ring. Examples of this are the reaction of 3-oxobutanethioamides **IV** with α-halogen ketones [25,26], the cyclocondensation of 1,3-dicarbonyl compounds or their synthetic equivalents **V** with mercaptanes [27,28], and Baylis–Hillman adducts **VI** with mercaptanes [29]. Despite the synthetic convenience of these approaches, however, they generally only allow the introduction of a limited number of non-functionalized substituents. Only a few examples of intramolecular cyclization of 2-substituted thiophenes **VII** [30,31,32], ring opening/annulation of cyclopropane derivatives **VIII** [33,34], and Rh-catalyzed C-H alkylation of the thiophene core **IX** are known [35].

The literature provides evidence that thiophene-3-yldiazonium salts obtained from the corresponding 3-aminothiophenes **X** can be valuable intermediates in thiophene chemistry. For example, Sandmeyer reactions [13,14,36,37,38], substitution of the diazo group by a carbonyl fragment [39], azo couplings [40], arylations of heterarenes [41,42,43], Heck-Matsuda couplings [44,45,46,47], photoinduced Ir (ppy)_3_-catalyzed alkyl sulfonations [48], arylation of arenes [49,50], gold-catalyzed cross-coupling with silanes, alkynes or boronic acids [51,52,53], and the synthesis of aryltriazenes have been investigated [54].

However, these numerous methods have serious disadvantages. First, they are limited to suitable and readily available precursors or expensive catalysts (Pd, Ir, or Rh complexes). Second, these methods are not suitable for introducing polyfunctional substituents into position 3 of the thiophene core, which are useful for the further construction of other heterocycles.

As part of a current project, we investigated whether the Meerwein reaction can overcome these difficulties. In principle, it enables the production of arenic building blocks that are suitable as starting materials for the construction of various heterocycles [55,56], as we have shown in previous work [57,58,59,60,61] with the aim of synthesizing antitumor active substances [62,63]. In the present work, the title compounds, which were prepared from methyl-3-aminothiophene-2-carboxylate as a precursor, enable the production of thiophene-3-yl-containing bifunctional compounds that are suitable for use in heterocyclization reactions. We show that the application potential of this starting material extends beyond C–H arylation, arylations, and oxyarylations of certain alkenes [64,65,66,67,68,69] and can be applied to the addition reaction of a thiophene-3-yl fragment and a halogen atom to the C=C bond of functionalized alkenes in a Meerwein-type reaction. Here, we report the preparation of thiophen-3-yl derivatives of thiazole, selenazole, and thiophene, which can be obtained in two steps from functionalized α,β-unsaturated compounds and 2-(methoxycarbonyl)thiophen-3-yldiazonium salts. We present first examples of Meerwein reactions with thiophen-3-yldiazonium salts here.

## 2. Results and Discussion

**Scheme 1:**
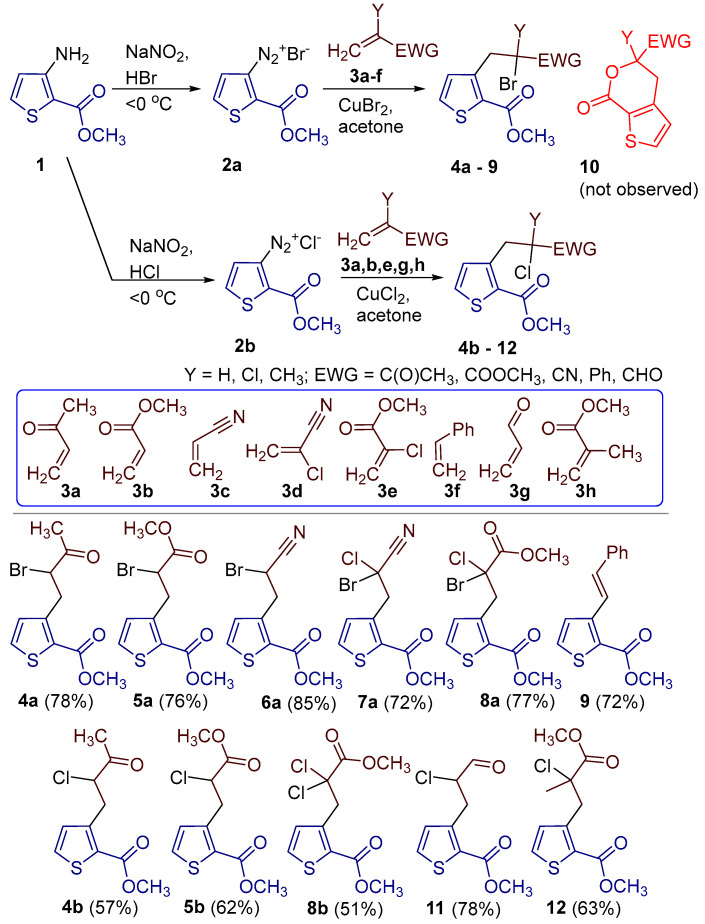
Gram-scale Meerwein halothienylation of unsaturated compounds **3a-h** with thiophen-3-yldiazonium salts **2**.

The reaction of 2-(methoxycarbonyl)thiophene-3-diazonium bromide with methyl vinyl ketone was selected as a model transformation and was carried out in the presence of CuBr_2_ as a catalyst. This diazonium salt was easily produced from 3-amino-2-(methoxycarbonyl)thiophene **1** by treating it with aqueous HBr, followed by diazotization with NaNO_2_ at −5 °C (Scheme 1). After 5 min at −5 to 0 °C, the solution of resulting 2-(methoxycarbonyl)thiophen-3-yl-diazonium bromide **2a** was used directly in a reaction with methyl vinyl ketone **3a** in acetone in the presence of CuBr_2_ (0.45 mol%). The mixture was stirred until the evolution of N_2_ ceased. The resulting ketone **4a** was extracted from the reaction mixture with CH_2_Cl_2_ and purified by distillation under reduced pressure (0.2 Torr). The targeted methyl 3-(2-bromo-3-oxobutyl)thiophene-2-carboxylate **4a** was obtained in a 78% yield.

The bromothienylations of other functionalized ethenes **3b–e** (methyl acrylate, acrylonitrile, 2-chloroacrylonitryl, methyl 3-chloroacrylate) with the diazonium bromide **2a** were carried out under analogous conditions. The resulting compounds **5a**–**8a** were purified by distillation under reduced pressure (0.2–0.5 torr). Distillations under slightly higher pressures (~2–5 torr) resulted in partial decomposition of the product and the formation of a resinous residue. However, the interaction of diazonium bromide **2a** with styrene **3f** leads to the formation of methyl 3-styrylthiophene-2-carboxylate **9**, and no product of the addition of a thiophene fragment and a bromine atom to the C=C bond was detected. 3-Styrylthiophene-2-carboxylate **9** has been previously synthesized by other methods, and the NMR data are in accordance with that reported in the literature [70,71].

An attempt to bromothienylate acrolein **3g** was unsuccessful and resulted in the formation of resinous substances of unknown structure. In contrast, the chlorothienylation of acrolein proceeds efficiently and leads to the formation of chloraldehyde **11** in a yield of 78%. We also performed chlorothienylation of methyl vinyl ketone, methyl acrylate, methyl methacrylate, and methyl 3-chloroacrylate. This reaction of diazonium chloride **2b** with the corresponding α,β-unsaturated functionalized compound **3** was carried out in acetone in the presence of CuCl_2_ × 2 H_2_O (29 mol%). The required 2-(methoxycarbonyl)thiophene-3-diazonium chloride **2b** was synthesized in situ from 3-amino-2-(methoxycarbonyl)thiophene, hydrochloric acid, and sodium nitrite in the temperature range of −5 to 0 °C. The compounds **4b**–**12** obtained were purified by distillation under reduced pressure (0.2–0.5 Torr). The structure of the synthesized compounds and their yields are shown in Scheme 1.

**Scheme 2:**
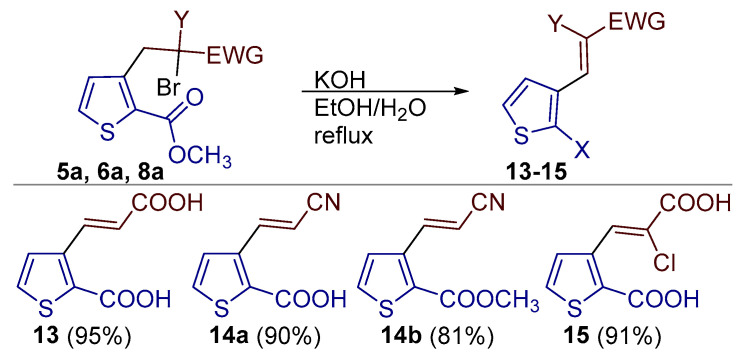
Synthesis of derivatives of (thiophen-3-yl)acrylic acids.

The presence of an *ortho*-methoxycarbonyl group in diazonium bromide **2a** opens up the possibility of forming dihydrothieno [2,3-*c*]pyranones **10** in the bromothiophene reaction of α,β-unsaturated functionalized compounds, although this was not achieved in all cases [66,67,68,69,72,73]. Since thieno [2,3-*c*]pyranones were not formed in the halothiophene reaction, we attempted to obtain them by treating the products **5a**, **6a**, and **8a** with a KOH solution while varying the reaction conditions [72,73]. Despite varying the solvents (methanol, alcohol, alcohol-water mixtures, aqueous dioxane, THF) and the reaction temperature (from 0 °C to reflux), only HBr cleavage was observed from compounds **5a** and **8a**, yielding derivatives of 3-(thiophen-3-yl)acrylic acids **13** and **15** (Scheme 2). Although the literature states that compound **13** is synthesized [74], the Supporting Information describes the diester (methyl (*E*)-3-(3-methoxy-3-oxoprop-1-en-1-yl)thiophene-2-carboxylate).

In the case of nitrile **6a**, treatment with KOH solution led to the formation of acid **14a** (this compound has been previously described [75]), while carrying out the reaction at 0 °C led to the isolation of ester **14b** (this compound has been previously described [76]). This indicates the extreme ease of HBr cleavage with the formation of a conjugated system. According to the ^1^H NMR data of **13**–**14b**, the elimination of hydrogen bromide mainly leads to the formation of the *E* isomer.

In all cases, the reaction with the base is exothermic and proceeds rapidly with the elimination of hydrogen bromide, probably due to the formation of a thermodynamically favorable conjugated system in thienylacrylic acid derivatives.

**Scheme 3:**
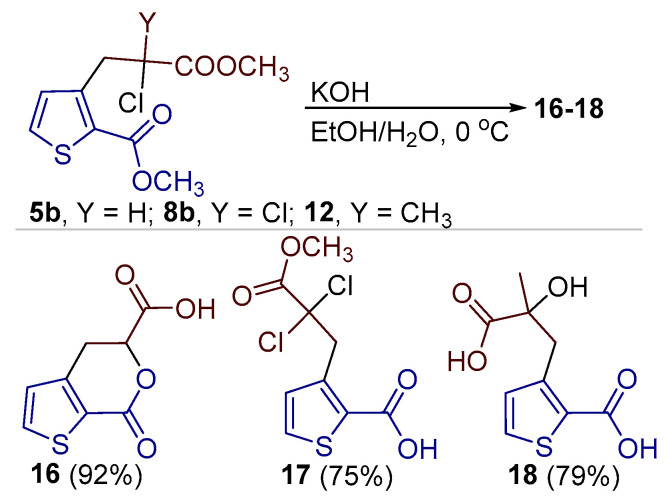
Hydrolysis of α-chloroesters **5b**, **8b**, and **12** at 0 °C.

Chlorinated ester **5b** reacted with a KOH solution under reflux to form a C=C bond in compound **13** with elimination of HCl. On the other hand, the reaction of ester **5b** with KOH at 0 °C led to 7-oxo-4,7-dihydro-5H-thieno [2,3-*c*]pyran-5-carboxylic acid **16** (Scheme 3). As the reaction is exothermic, the reaction mixture must be cooled intensively and stirred vigorously to prevent the elimination of hydrogen chloride and the formation of thienylacrylic acid. Under the same conditions, ester **8b** was converted to 3-(2,2-dichloro-3-methoxy-3-oxopropyl)thiophene-2-carboxylic acid **17**, and **7b** was converted to 3-(2-carboxy-2-hydroxypropyl)thiophene-2-carboxylic acid **18**. It should be noted that compound **18** is highly soluble in water. The interaction of compounds **4b** and **11b** with KOH solution led to the formation of resinous substances of unknown structure.

It appears that the type of halogen atom in halothienylation products is decisive for the course of either the elimination or substitution reaction. Substitution products **16** and **18** have been obtained from chlorothienylation products only.

**Scheme 4:**
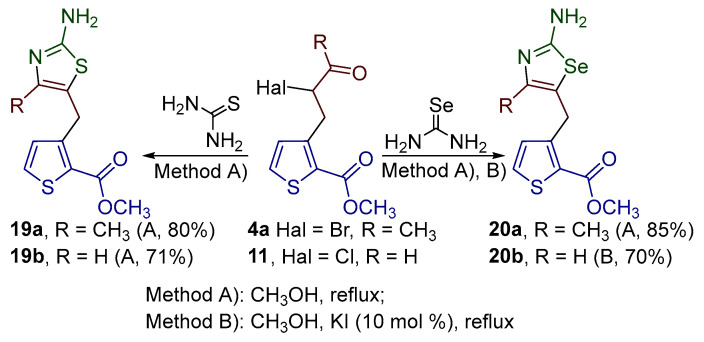
Syntheses of 3-thienyl containing derivatives of 2-aminothia(selena)zoles.

We then investigated the synthetic potential of the products of halothienylation for the construction of more complex heterocyclic systems. Although halocarbonyl compounds **4** and **11** proved to be labile to strong bases, they are undoubtedly suitable as bifunctional precursors for constructing a thiazole cycle. Thus, bromoketone **4a** and chloroaldehyde **11** reacted smoothly with thiourea in an alcoholic medium to form aminothiazoles **19a** and **19b**, respectively (Scheme 4). Conversely, chloroketone **4b** did not interact with thiourea, even when the reaction mixture was refluxed for an extended period. However, in the presence of KI (10 mol%) the target aminothiazole is nevertheless formed, but with a low yield (42%). The reaction of bromoketone **4a** with selenourea proceeded smoothly with the formation of aminoselenazole **20a**. However, chloroaldehyde **11** only interacted with selenourea when catalytic amounts of KI (10 mol%) were added.

**Scheme 5:**
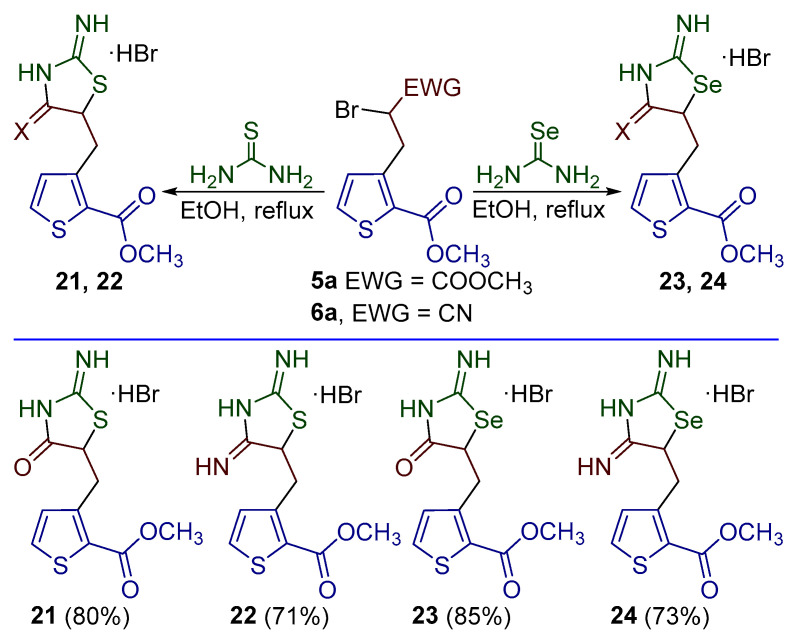
Syntheses of 3-thienyl derived thia(selena)zolidines.

We also obtained in a similar way thiazolidines and selenazolidines, by the reaction of α-bromoester **5a** and α-bromonitrile **6a** with thiourea or selenourea, respectively. Thus, we obtained compounds **21–24** as hydrobromides, which crystallized from the reaction mixture when cooled (Scheme 5).

It should be noted that the structure of many practically useful compounds contains a combination of thiophene and other heterocyclic rings. For example, substances whose molecules contain thiophene and thiazole rings are being studied as photoswitches [77], photovoltaic materials [78], angiotensin AT2 receptor ligands [79], and exhibit anticancer activity [80,81]. Compounds containing 3-thienyl and thiazolone fragments are antitumor [82] and anti-neuroinflammatory agents [83].

Since propionitrile **7a** and propanoate **8a** are synthetic equivalents of the 3C synthon, which are suitable for building a thiophene ring and thus for the synthesis of bithiophenes, we carried out further reactions. This was also driven by the fact that substituted 2,3′-bithiophene compounds have been developed as antibacterial [84] and antitumor compounds [85], pan-ERR agonists [86], and protein tyrosine phosphatase 1B inhibitors [87]. These are mainly derivatives of 4-amino- and 4-hydroxy-2,3′-bithiophene compounds [85,87]. 2,3′-Bithiophene derivatives are usually obtained using halothiophene and thienylboronic acids in the Suzuki reaction [84,86,87]. Acrylonitrile derivatives obtained from acetylthiophene are only used occasionally [85]. This severely limits the range of available functionalized 2,3′-bithiophenes. Therefore, the search for new substrates for the construction of such compounds remains an important task.

**Scheme 6:**
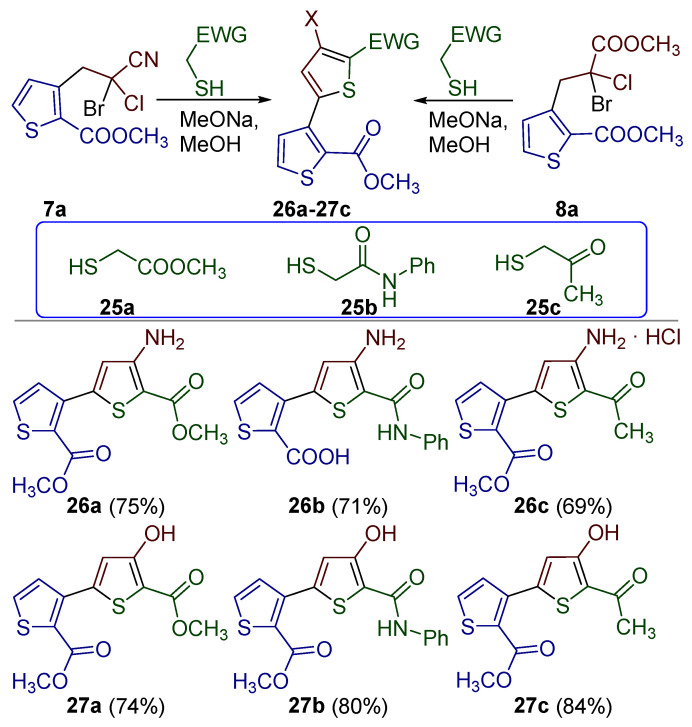
Syntheses of 4-amino- and 4-hydroxy-2,3′-bithiophenes.

For the compounds **7a** and **8a**, the reaction with methylthioglycolate **25a**, mercaptoacetanilide **25b** or mercaptoacetone **25c** in methanol in the presence of sodium methylate as a base provided access to substituted 4-amino-2,3′-bithiophenes **26** and 4-hydroxy-2,3′-bithiophenes **27** (Scheme 6). In all cases, the reaction proceeded smoothly within two hours and yielded the corresponding 2,3′-bithiophenes. However, when nitrile **7** interacted with mercaptoacetanilide **25b**, only acid **26b** was obtained as a result of the hydrolysis of the target ester during separation from the reaction mixture. Amine **26c** did not crystallize when separated from the reaction mixture, so we obtained it as a hydrochloride. Therefore, the 2,3′-bithiophenes **26–27** were prepared as stable compounds in moderate to good yields, differing in their substituents at the C-5 position and bearing a methoxycarbonyl group at the C-2′ position of the 2,3′-bithiophene core.

In summary, we describe the first general method for using 2-(methoxycarbonyl)thiophene-3-diazonium salts in the Meerwein reaction for the synthesis of bifunctional compounds that are suitable building blocks for the development of thiophene-containing compounds. A wide range of starting materials, including unsaturated compounds and binucleophiles, allowed us to achieve structural diversity in the 3-position of the thiophene ring that had previously been difficult to achieve by other routes. Thus, our method provides a smooth and straightforward route to 5-(thiophen-3-yl)methylene-substituted 2-aminothiazoles, 2-aminoselenazoles, thiazolidines and selenazolidines, as well as derivatives of thiophene-3-ylacrylic acids, thieno [2,3-*c*]pyrane, 4-amino- and 4-hydroxy-2,3′-bithiophenes.

## 3. Materials and Methods

Commercially available reagents and solvents were obtained from TCI (TCI, Haven 1063, 2070 Zwijndrecht, Belgium), J&K (J&K, Marbach, Germany), and Aldrich (Merck KGaA, Darmstadt, Germany) and used without further purification unless otherwise specified.

Reported yields are isolated and have not been optimized. Melting points were determined using a Büchi apparatus (Büchi, Uster, Switzerland) and are reported uncorrected.

Thin-layer chromatography (TLC) was carried out on Merck (Merck KGaA, Darmstadt, Germany) silica gel 60 F_254_ aluminum plates, with visualization under UV light.

^1^H and ^13^C NMR spectra were obtained on a Bruker 170 Avance 500 spectrometer (at 500 MHz for ^1^H, 126 MHz for ^13^C), and a Bruker Avance 400 MHz spectrometer (at 400 MHz for ^1^H, 101 MHz for ^13^C) (Bruker, Bremen, Germany). Chemical shifts (δ) are reported in parts per million (ppm) relative to internal standard or solvent signal. Signal multiplicities are denoted as follows: s = singlet, br.s = broad singlet, d = doublet, dd = doublet of doublets, t = triplet, q = quartet, m = multiplet. Signal orientations in DEPT experiments are indicated as: o = no signal; + = positive (CH, CH_3_); − = negative (CH_2_).

Electrospray ionization mass spectra (ESI-MS) were recorded on a Bruker Impact II mass spectrometer using MeCN as the solvent.

ATR-IR spectra were recorded on a Shimadzu IRSpirit-T spectrometer (Shimadzu, Kyoto, Japan) over the range of 400–4000 cm^−1^.

**General Procedure for the Preparation of the Methyl 3-(2-bromo- 3-oxobutyl)thiophene-2-carboxylate 4a, Methyl 3-(2-bromo-3-methoxy-3-oxopropyl)-thiophene-2-carboxylate 5a, Methyl 3-(2-bromo-2-cyanoethyl)thiophene-2-carboxylate 6a, Methyl 3-(2-bromo-2-chloro-2-cyanoethyl)thiophene-2-carboxylate 7a, Methyl 3-(2-bromo-2-chloro-3-methoxy-3-oxopropyl)thiophene-2-carboxylate 8a, and Methyl (*E*)-3-styrylthiophene-2-carboxylate 9.**

The methyl 3-aminothiophene-2-carboxylate **1** (3.14 g, 20 mmol, 1.0 equiv.) was added to 46% aqueous hydrobromic acid (5.1 mL, 42 mmol, 1.1 equiv.). The resulting mixture was cooled to −5 °C, followed by the dropwise addition of a saturated solution of sodium nitrite (1.45 g, 21 mmol, 1.05 equiv.), ensuring that the temperature remained below 5 °C throughout the addition. The freshly obtained cold diazonium salt solution **2a** was then added dropwise to a solution containing the corresponding functionalized alkene **3** (20 mmol, 1.0 equiv.) and CuBr_2_ (20 mg, 0.09 mmol) in acetone (20 mL) under vigorous stirring throughout. The reaction mixture was stirred at room temperature (20 °C) until the evolution of nitrogen gas ceased to evolve. Subsequently, 100 mL of water was added, and the organic layer was separated. The aqueous phase was extracted with CH_2_Cl_2_ (5 × 10 mL), and the combined organic extracts were dried over anhydrous MgSO_4_. The solvent was then removed under reduced pressure, and the product was purified by distillation under vacuum.

**Methyl 3-(2-bromo-3-oxobutyl)thiophene-2-carboxylate 4a**.

Yield 4.543 g (78%), colorless oil or white solid, bp 138–141 °C/0.25 torr, mp 52–53 °C.

^1^H NMR (400 MHz, [D_6_]DMSO): δ = 7.83 (d, *J* = 5.1 Hz, 1H, H-thiophene), 7.17 (d, *J* = 5.1 Hz, 1H, H-thiophene), 4.96 (dd, *J* = 8.6, 6.1 Hz, 1H, CH), 3.81 (s, 3H, CH_3_), 3.64 (dd, *J* = 14.7, 6.1 Hz, 1H, CH_2_), 3.57 (dd, *J* = 14.7, 8.6 Hz, 1H, CH_2_), 2.32 (s, 3H, CH_3_).

^13^C NMR (101 MHz, [D_6_]DMSO): δ = 201.3, 162.1, 145.3, 131.7, 131.3, 127.2, 53.0, 52.0, 32.2, 26.7.

MS (ESI): *m*/*z* = 291.0 [M + H]^+^.

IR (ATR) ν_max_ = 1718 (C=O), 1701 (C=O) cm^−1^.

Anal. Calcd for C_10_H_11_BrO_3_S: C, 41.25; H, 3.81. Found: C, 41.14; H, 3.73.

**Methyl 3-(2-bromo-3-methoxy-3-oxopropyl)thiophene-2-carboxylate 5a**.

Yield 4.670 g (76%), light-yellow oil, bp 143–146 °C/0.5 torr.

^1^H NMR (400 MHz, [D_6_]DMSO): δ = 7.83 (d, *J* = 5.1 Hz, 1H, H-thiophene), 7.16 (d, *J* = 5.1 Hz, 1H, H-thiophene), 4.81 (t, *J* = 7.6 Hz, 1H, CH), 3.81 (s, 3H, CH_3_), 3.84–3.78 (m, 1H, CH_2_), 3.69 (s, 3H, CH_3_), 3.67 (d, *J* = 7.6 Hz, 1H, CH_2_).

^1^H NMR (500 MHz, CDCl_3_): δ = 7.42 (d, *J* = 5.0 Hz, 1H, H-thiophene), 7.00 (d, *J* = 5.0 Hz, 1H, H-thiophene), 4.62 (t, *J* = 7.6 Hz, 1H, CH), 3.87 (s, 3H, CH_3_), 3.79–3.74 (m, 1H, CH_2_), 3.73 (s, 3H, CH_3_), 3.63 (dd, *J* = 13.8, 7.6 Hz, 1H, CH_2_).

^13^C NMR (101 MHz, [D_6_]DMSO): δ = 169.4, 162.0, 144.6, 131.8, 131.2, 127.5, 52.9, 52.1, 44.5, 33.8.

^13^C NMR (126 MHz, CDCl_3_): δ = 170.1, 162.7, 144.8, 131.8, 130.6, 128.5, 53.0, 52.2, 43.8, 34.9.

MS (ESI): *m*/*z* = 307.0 [M + H]^+^.

IR (ATR) ν_max_ = 1737 (C=O), 1702 (C=O) cm^−1^.

Anal. Calcd for C_10_H_11_BrO_4_S: C, 39.10; H, 3.61. Found: C, 39.01; H, 3.55.

**Methyl 3-(2-bromo-2-cyanoethyl)thiophene-2-carboxylate 6a**.

Yield 4.659 g (85%), light-yellow crystalline solid, bp 149–151 °C/0.5 torr, mp 51–52 °C.

^1^H NMR (400 MHz, [D_6_]DMSO): δ = 7.90 (d, *J* = 5.1 Hz, 1H, H-thiophene), 7.27 (d, *J* = 5.1 Hz, 1H, H-thiophene), 5.31 (dd, *J* = 8.1, 7.1 Hz, 1H, CH), 3.84 (dd, *J* = 13.9, 8.1 Hz, 1H, CH_2_), 3.82 (s, 3H, CH_3_), 3.77 (dd, *J* = 13.9, 7.1 Hz, 1H, CH_2_).

^13^C NMR (101 MHz, [D_6_]DMSO): δ = 162.0, 142.7, 132.2, 131.2, 128.5, 117.9, 52.2, 34.7, 26.9.

MS (ESI): *m*/*z* = 274.0 [M + H]^+^.

IR (ATR) ν_max_ = 2243 (CN), 1687 (C=O) cm^−1^.

Anal. Calcd for C_9_H_8_BrNO_2_S: C, 39.43; H, 2.94. Found: C, 39.35; H, 2.85.

**Methyl 3-(2-bromo-2-chloro-2-cyanoethyl)thiophene-2-carboxylate 7a**.

Yield 4.451 g (72%), white crystalline solid, bp 128–130 °C/0.2 torr, mp 74–75 °C.

^1^H NMR (400 MHz, [D_6_]DMSO): δ = 7.96 (d, *J* = 5.1 Hz, 1H, H-thiophene), 7.35 (d, *J* = 5.1 Hz, 1H, H-thiophene), 4.53 (d, *J* = 14.3 Hz, 1H, CH_2_), 4.46 (d, *J* = 14.3 Hz, 1H, CH_2_), 3.83 (s, 3H, CH_3_).

^1^H NMR (400 MHz, MeOD): δ = 7.74 (d, *J* = 5.2 Hz, 1H, H-thiophene), 7.35 (d, *J* = 5.1 Hz, 1H, H-thiophene), 4.53 (d, *J* = 14.3 Hz, 1H, CH_2_), 4.45 (d, *J* = 14.3 Hz, 1H, CH_2_), 3.88 (s, 3H, CH_3_).

^13^C NMR (101 MHz, [D_6_]DMSO): δ = 162.0, 139.4, 132.1, 131.6, 130.3, 116.2, 52.3, 49.3, 44.5.

^13^C NMR (101 MHz, MeOD): δ = 163.9 (o), 140.6 (o), 132.6 (+), 132.3 (o), 132.2 (+), 117.2 (o), 52.7 (+), 50.9 (o), 46.7 (+).

MS (ESI): *m*/*z* = 307.9 [M + H]^+^.

IR (ATR) ν_max_ = 2229 (CN), 1702 (C=O) cm^−1^.

Anal. Calcd for C_9_H_7_BrClNO_2_S: C, 35.03; H, 2.29; N, 4.54. Found: C, 34.92; H, 2.23; N, 4.50.

**Methyl 3-(2-bromo-2-chloro-3-methoxy-3-oxopropyl)thiophene-2-carboxylate 8a**.

Yield 5.260 g (77%), light-yellow oil, bp 150–153 °C/0.2 torr.

^1^H NMR (400 MHz, [D_6_]DMSO): δ = 7.87 (d, *J* = 5.1 Hz, 1H, H-thiophene), 7.18 (d, *J* = 5.1 Hz, 1H, H-thiophene), 4.50 (d, *J* = 14.7 Hz, 1H, CH_2_), 4.26 (d, *J* = 14.7 Hz, 1H, CH_2_), 3.87 (s, 3H, CH_3_), 3.81 (s, 3H, CH_3_).

^13^C NMR (126 MHz, [D_6_]DMSO): δ = 166.2, 162.1, 140.9, 131.5, 130.9, 129.8, 70.7, 54.8, 52.1, 42.5.

MS (ESI): *m*/*z* = 340.9 [M + H]^+^.

IR (ATR) ν_max_ = 1741 (C=O), 1708 (C=O) cm^−1^.

Anal. Calcd for C_10_H_10_BrClO_4_S: C, 35.16; H, 2.95. Found: C, 35.05; H, 2.88.

**Methyl (*E*)-3-styrylthiophene-2-carboxylate 9**.

Yield 3.524g (72%), white crystalline solid, bp 145–149 °C/0.25 torr, mp 85–86 °C.

^1^H NMR (400 MHz, [D_6_]DMSO): δ = 8.02 (d, *J* = 16.6 Hz, 1H, CH=), 7.87 (dd, *J* = 5.3, 0.5 Hz, 1H, 4H-thiophene), 7.72 (d, *J* = 5.3 Hz, 1H, 5H-thiophene), 7.58 (d, *J* = 7.3 Hz, 2H, C_6_H_5_), 7.44–7.35 (m, 3H, C_6_H_5_, CH=), 7.37–7.27 (m, 1H, C_6_H_5_), 3.85 (s, 3H, CH_3_).

^13^C NMR (101 MHz, [D_6_]DMSO): δ = 162.3, 145.1, 136.6, 133.3, 132.0, 128.9, 128.4, 126.7, 125.5, 121.2, 52.1.

MS (ESI): *m*/*z* = 245.1 [M + H]^+^.

IR (ATR) ν_max_ = 1722 (C=O) cm^−1^.

NMR data are in accordance with that reported in the literature [70,71].

**General Procedure for the Preparation of Methyl 3-(2-chloro-3-oxobutyl)thiophene-2-carboxylate 4b, Methyl 3-(2-chloro-3-methoxy-3-oxopropyl)thiophene-2-carboxylate 5b, Methyl 3-(2,2-dichloro-3-methoxy-3-oxopropyl)thiophene-2-carboxylate 8b, Methyl 3-(2-chloro-3-oxopropyl)thiophene-2-carboxylate 11, and Methyl 3-(2-chloro-3-methoxy-2-methyl-3-oxopropyl)thiophene-2-carboxylate 12.**

The methyl 3-aminothiophene-2-carboxylate **1** (3.14 g, 20 mmol, 1.0 equiv.) was added to 37% aqueous hydrochloric acid (3.54 mL, 42 mmol, 2.1 equiv.). The resulting mixture was cooled to −5 °C, followed by the dropwise addition of a saturated solution of sodium nitrite (1.45 g, 21 mmol, 1.05 equiv.), ensuring that the temperature remained below 5 °C throughout the addition. The freshly obtained cold diazonium salt solution **2a** was then added dropwise to a solution containing the corresponding functionalized alkene **3** (20 mmol, 1.0 equiv.) and CuCl_2_×2H_2_O (1.00 g, 5.8 mmol) in acetone (20 mL) under vigorous stirring throughout. The reaction mixture was stirred at room temperature (20 °C) until the evolution of nitrogen gas ceased to evolve. Subsequently, 100 mL of water was added, and the organic layer was separated. The aqueous phase was extracted with CH_2_Cl_2_ (5 × 10 mL), and the combined organic extracts were dried over anhydrous MgSO_4_. The solvent was then removed under reduced pressure, and the product was purified by distillation under reduced pressure.

**Methyl 3-(2-chloro-3-oxobutyl)thiophene-2-carboxylate 4b**.

Yield 2.830 g (57%), light-yellow oil, bp 137–140 °C/0.5 torr.

^1^H NMR (400 MHz, [D_6_]DMSO): δ = 7.82 (d, *J* = 5.1 Hz, 1H, H-thiophene), 7.17 (d, *J* = 5.1 Hz, 1H, H-thiophene), 4.89 (dd, *J* = 9.2, 5.2 Hz, 1H, CH), 3.81 (s, 3H, OCH_3_), 3.64 (dd, *J* = 14.4, 5.2 Hz, 1H, CH_2_), 3.43 (dd, *J* = 14.4, 9.2 Hz, 1H, CH_2_), 2.31 (s, 3H, CH_3_).

^13^C NMR (101 MHz, [D_6_]DMSO): δ = 201.8, 162.1, 144.7, 131.7, 131.6, 127.3, 62.9, 52.0, 32.4, 26.6.

MS (ESI): *m*/*z* = 247.0 [M + H]^+^.

IR (ATR) ν_max_ = 1704 (C=O) cm^−1^.

Anal. Calcd for C_10_H_11_ClO_3_S: C, 48.69; H, 4.49. Found: C, 48.60; H, 4.39.

**Methyl 3-(2-chloro-3-methoxy-3-oxopropyl)thiophene-2-carboxylate 5b**.

Yield 3.261 g (62%), colorless oil or crystalline solid, bp 130–133 °C/0.5 torr, mp 32–33 °C.

^1^H NMR (400 MHz, [D_6_]DMSO): δ = 7.84 (d, *J* = 5.1 Hz, 1H, H-thiophene), 7.17 (d, *J* = 5.1 Hz, 1H, H-thiophene), 4.87 (dd, *J* = 8.4, 6.3 Hz, 1H, CH), 3.81 (s, 3H, CH_3_), 3.70 (s, 3H, CH_3_), 3.69–3.65 (m, 1H, CH_2_), 3.61 (dd, *J* = 10.9, 7.3 Hz, 1H, CH_2_).

^13^C NMR (101 MHz, [D_6_]DMSO): δ = 169.1 (o), 162.1 (o), 143.8 (o), 131.8 (+), 131.4 (+), 127.6 (o), 56.1 (+), 52.9 (+), 52.1 (+), 33.7 (−).

MS (ESI): *m*/*z* = 263.0 [M + H]^+^.

IR (ATR) ν_max_ = 1742 (C=O), 1702 (C=O) cm^−1^.

Anal. Calcd for C_10_H_11_ClO_4_S: C, 45.72; H, 4.22. Found: C, 45.63; H, 4.13.

**Methyl 3-(2,2-dichloro-3-methoxy-3-oxopropyl)thiophene-2-carboxylate 8b**.

Yield 3.030 g (51%), colorless oil or crystalline solid, bp 139–142 °C/ 0.5 torr, mp 34–35 °C.

^1^H NMR (400 MHz, [D_6_]DMSO): δ = 7.86 (d, *J* = 5.1 Hz, 1H, H-thiophene), 7.19 (d, *J* = 5.2 Hz, 1H, H-thiophene), 4.29 (s, 2H, CH_2_), 3.87 (s, 3H, CH_3_), 3.80 (s, 3H, CH_3_).

^13^C NMR (126 MHz, [D_6_]DMSO): δ = 165.7, 162.2, 140.1, 131.5, 131.4, 129.9, 83.5, 54.9, 52.2, 41.8.

MS (ESI): *m*/*z* = 297.0 [M + H]^+^.

IR (ATR) ν_max_ = 1755 (C=O), 1707 (C=O) cm^−1^.

Anal. Calcd for C_10_H_10_Cl_2_O_4_S: C, 40.42; H, 3.39. Found: C, 40.34; H, 3.31.

**Methyl 3-(2-chloro-3-oxopropyl)thiophene-2-carboxylate 11**.

Yield 3.640 g (78%), light-yellow oil, bp 119–124 °C/0.25 torr.

^1^H NMR (400 MHz, CDCl_3_): δ = 9.52 (d, J = 1.7, 1H, CHO), 7.44 (d, *J* = 5.0 Hz, H-thiophene), 7.01 (d, *J* = 5.0 Hz, H-thiophene), 4.61–4.49 (m, 1H, CH), 3.87–3.75 (m, 4H, OCH_3_, CH_2_), 3.33 (dd, *J* = 14.1, 8.5 Hz, 1H, CH_2_).

^13^C NMR (101 MHz, CDCl_3_): δ = 193.7, 162.7, 143.7, 131.7, 130.8, 127.8, 62.7, 52.1, 32.0.

MS (ESI): *m*/*z* = 233.0 [M + H]^+^.

IR (ATR) ν_max_ = 1698 (C=O) cm^−1^.

Anal. Calcd for C_9_H_9_ClO_3_S: C, 46.46; H, 3.90. Found: C, 46.38; H, 3.80.

**Methyl 3-(2-chloro-3-methoxy-2-methyl-3-oxopropyl)thiophene-2-carboxylate 12**.

Yield 3.485 g (63%), light-yellow oil or crystalline solid, bp 130–134 °C/0.25 torr, mp 38–39 °C.

^1^H NMR (400 MHz, CDCl_3_): δ = 7.43 (d, *J* = 5.1 Hz, 1H, H-thiophene), 7.09 (d, *J* = 5.1 Hz, 1H, H-thiophene), 3.95 (d, *J* = 14.1 Hz, 1H, CH_2_), 3.86 (s, 3H, CH_3_), 3.82 (d, *J* = 14.1 Hz, 1H, CH_2_), 3.80 (s, 3H, CH_3_), 1.68 (s, 3H, CH_3_).

^13^C NMR (101 MHz, CDCl_3_): δ = 171.6 (o), 162.9 (o), 143.1 (o), 131.8 (+), 130.2 (+), 129.6 (o), 68.3 (o), 53.4 (+), 52.1 (+), 39.4 (−), 26.7 (+).

MS (ESI): *m*/*z* = 277.0 [M + H]^+^.

IR (ATR) ν_max_ = 1738 (C=O), 1701 (C=O) cm^−1^.

Anal. Calcd for C_11_H_13_ClO_4_S: C, 47.74; H, 4.74. Found: C, 47.66; H, 4.67.

**General Procedure for the Preparation of (*E*)-3-(2-carboxyvinyl)thiophene- 2-carboxylic acid 13, Methyl (*E*)-3-(2-cyanovinyl)thiophene-2-carboxylate 14b, and 3-(2-Carboxy-2-chlorovinyl)thiophene-2-carboxylic acid 15**

The solution of the corresponding methyl 3-(2-bromo-3-methoxy-3-oxopropyl)thiophene-2-carboxylate **5a**, or methyl 3-(2-bromo-2-cyanoethyl)thiophene-2-carboxylate **6a**, or methyl 3-(2-bromo-2-chloro-3-methoxy-3-oxopropyl)thiophene-2-carboxylate **8a** (5 mmol, 1.0 equiv.) in 5 mL methanol was added dropwise under vigorous stirring to the mixture of KOH (0.98 g, 17.5 mmol, 3.5 equiv.), methanol (5 mL), and water (10 mL) at 0 °C. Then the mixture was stirred 1.5 h. After that, the reaction mixture was cooled and poured into a mixture of 10 mL of 37% HCl and 20 g of ice. The product was filtered off and recrystallized from methanol or methanol-H_2_O.

**(*E*)-3-(2-Carboxyvinyl)thiophene-2-carboxylic acid 13**.

Yield 0.945 g (95%), white crystalline solid, mp > 300 °C.

^1^H NMR (400 MHz, [D_6_]DMSO): δ = 13.30–12.75 (m, 2H, 2COOH), 8.39 (d, *J* = 16.1 Hz, 1H, =CH), 7.83 (dd, *J* = 5.3, 0.6 Hz, 1H, 4H-thiophene), 7.69 (d, *J* = 5.3 Hz, 1H, 5H-thiophene), 6.54 (d, *J* = 16.1 Hz, 1H, =CH).

^13^C NMR (101 MHz, [D_6_]DMSO): δ = 167.7 (o), 162.9 (o), 141.1 (o), 135.9 (+), 132.2 (o), 131.7 (+), 127.5 (+), 122.7 (+).

MS (ESI): *m*/*z* = 199.0 [M + H]^+^.

IR (ATR) ν_max_ = 1685 (C=O), 1648 (C=O) cm^−1^.

Anal. Calcd for C_8_H_6_O_4_S: C, 48.48; H, 3.05. Found: C, 48.43; H, 2.95.

Although the literature states that compound **13** is synthesized, the Supporting Information describes the diester (methyl (*E*)-3-(3-methoxy-3-oxoprop-1-en-1-yl)thiophene-2-carboxylate) [73].

**Methyl (*E*)-3-(2-cyanovinyl)thiophene-2-carboxylate 14b**.

Yield 0.781 g (81%), white crystalline solid, mp 126–127 °C.

^1^H NMR (400 MHz, [D_6_]DMSO): δ = 8.14 (d, *J* = 16.8 Hz, 1H, =CH), 7.94 (d, *J* = 4.9 Hz, 1H, H-thiophene), 7.66 (d, *J* = 4.8 Hz, 1H, H-thiophene), 6.55 (d, *J* = 16.8 Hz, 1H, =CH), 3.84 (s, 3H, CH_3_).

^13^C NMR (101 MHz, [D_6_]DMSO): δ = 161.6, 141.7, 140.6, 132.8, 130.6, 126.8, 118.4, 101.0, 52.5.

MS (ESI): *m*/*z* = 194.0 [M + H]^+^.

IR (ATR) ν_max_ = 2217 (CN), 1697 (C=O) cm^−1^.

Anal. Calcd for C_9_H_7_NO_2_S: C, 55.95; H, 3.65; N, 7.25. Found: C, 55.88; H, 3.61; N, 7.15.

Melting point and NMR data are in accordance with that reported in the literature [75].

**3-(2-Carboxy-2-chlorovinyl)thiophene-2-carboxylic acid 15**.

Yield 1.060 g (91%), white crystalline solid, mp 240–241 °C.

^1^H NMR (400 MHz, [D_6_]DMSO): δ = 14.00–12.94 (m, 2H, 2COOH), 8.73 (s, 1H, =CH), 7.92 (d, *J* = 5.3 Hz, 1H, H-thiophene), 7.90 (d, *J* = 5.3 Hz, 1H, H-thiophene).

^13^C NMR (101 MHz, [D_6_]DMSO): δ = 163.9, 162.7, 138.0, 134.2, 131.6, 129.7, 129.0, 124.0.

MS (ESI): *m*/*z* = 233.0 [M + H]^+^.

IR (ATR) ν_max_ = 1674 (C=O) cm^−1^.

Anal. Calcd for C_8_H_5_ClO_4_S: C, 41.30; H, 2.17. Found: C, 41.23; H, 2.13.

**Procedure for the Preparation of (*E*)-3-(2-Cyanovinyl)thiophene-2-carboxylic acid 14a**

The solution of methyl 3-(2-bromo-2-cyanoethyl)thiophene-2-carboxylate **6a** (0.548 g, 2 mmol, 1.0 equiv.) in 3 mL of methanol was added dropwise under vigorous stirring to the mixture of KOH (0.39 g, 7 mmol, 3.5 equiv.), methanole (2 mL), and water (5 mL) at 60 °C. Then the mixture was stirred under reflux 1 h. After that, the reaction mixture was cooled and poured into a mixture of 4 mL of 37% HCl and 20 g of ice. The product was filtered off and recrystallized from methanol.

**(*E*)-3-(2-Cyanovinyl)thiophene-2-carboxylic acid 14a**.

Yield 0.325 g (90%), white crystalline solid, mp 200–201 °C.

^1^H NMR (400 MHz, [D_6_]DMSO): δ = 14.29–12.93 (br.s, 1H, COOH), 8.22 (d, *J* = 16.7 Hz, 1H, =CH), 7.87 (d, *J* = 4.4 Hz, 1H, H-thiophene), 7.63 (d, *J* = 4.5 Hz, 1H, H-thiophene), 6.50 (d, *J* = 16.8 Hz, 1H, =CH).

^13^C NMR (101 MHz, [D_6_]DMSO): δ = 162.7, 142.2, 140.0, 132.8, 132.1, 126.7, 118.6, 100.3.

MS (ESI): *m*/*z* = 180.0 [M + H]^+^.

IR (ATR) ν_max_ = 2216 (CN), 1655 (C=O) cm^−1^.

Anal. Calcd for C_8_H_5_NO_2_S: C, 53.62; H, 2.81; N, 7.82. Found: C, 53.55; H, 2.78; N, 7.76.

NMR data are in accordance with that reported in the literature [75].

**General Procedure for the Preparation of 7-Oxo-4,7-dihydro- 5H-thieno [2,3-*c*]pyran-5-carboxylic acid 16, 2,2-Dichloro-3-(2-(methoxy- carbonyl)thiophen-3-yl)propanoic acid 17, and 3-(2-Carboxy-2-hydroxypropyl)-thiophene- 2-carboxylic acid 18** **.**

The solution of the corresponding methyl 3-(2-chloro-3-methoxy-3-oxopropyl)thiophene-2-carboxylate **5b**, methyl 3-(2,2-dichloro-3-methoxy-3-oxopropyl)thiophene-2-carboxylate **8b**, or methyl 3-(2-chloro-3-methoxy-2-methyl-3-oxopropyl)thiophene-2-carboxylate **12** (5 mmol, 1.0 equiv.) in 5 mL of methanol was added dropwise under vigorous stirring to the mixture of KOH (0.98 g, 17.5 mmol, 3.5 equiv.), methanol (5 mL), and water (10 mL) at 0 °C. Then the mixture was stirred 1.5 h. After that, the reaction mixture was cooled and poured into a mixture of 10 mL of 37% HCl and 20 g of ice. The product was filtered off and recrystallized from methanol or methanol-DMF.

In the case of the reaction of methyl 3-(2-chloro-3-methoxy-2-methyl-3-oxopropyl)thiophene-2-carboxylate **12**, after acidification the water was evaporated under reduced pressure from the reaction mixture. The resulting precipitate was extracted with dichloromethane (5 × 10 mL). The combined organic extracts were dried over MgSO_4_, evaporated, and the residue was recrystallized from hexane to obtain **18**.

**7-Oxo-4,7-dihydro-5H-thieno [2,3-*c*]pyran-5-carboxylic acid 16**.

Yield 0.915 g (92%), white solid, mp 190–191 °C.

^1^H NMR (400 MHz, [D_6_]DMSO): δ = 13.96–12.96 (br.s, 1H, COOH), 8.03 (d, *J* = 5.0 Hz, 1H, H-thiophene), 7.18 (d, *J* = 5.0 Hz, 1H, H-thiophene), 5.39 (dd, *J* = 6.1, 4.2 Hz, 1H, CH), 3.39 (dd, *J* = 17.3, 6.1 Hz, 1H, CH_2_), 3.28 (dd, *J* = 17.3, 4.2 Hz, 1H, CH_2_).

^13^C NMR (101 MHz, [D_6_]DMSO): δ = 170.9 (o), 159.5 (o), 145.8 (+), 135.6 (+), 127.6 (+), 125.6 (o), 75.6 (+), 27.1 (−).

MS (ESI): *m*/*z* = 199.0 [M + H]^+^.

IR (ATR) ν_max_ = 1715 (C=O) cm^−1^.

Anal. Calcd for C_8_H_6_O_4_S: C, 48.48; H, 3.05. Found: C, 48.40; H, 2.97.

**3-(2,2-Dichloro-3-methoxy-3-oxopropyl)thiophene-2-carboxylic acid 17**.

Yield 1.067 g (75%), white crystalline solid, mp 97–98 °C.

^1^H NMR (400 MHz, [D_6_]DMSO): δ = 7.85 (d, *J* = 5.1 Hz, 1H, H-thiophene), 7.22 (d, *J* = 5.2 Hz, 1H, H-thiophene), 4.25 (s, 2H, CH_2_), 3.80 (s, 3H, CH_3_).

^13^C NMR (101 MHz, [D_6_]DMSO): δ = 166.6, 162.2, 140.7, 131.3, 131.3, 129.9, 84.6, 52.2, 41.8.

MS (ESI): *m*/*z* = 283.0 [M + H]^+^.

IR (ATR) ν_max_ = 1737 (C=O), 1698 (C=O) cm^−1^.

Anal. Calcd for C_9_H_8_Cl_2_O_4_S: C, 38.18; H, 2.85. Found: C, 38.07; H, 2.80.

**3-(2-Carboxy-2-hydroxypropyl)thiophene-2-carboxylic acid 18**.

Yield 0.911 g (79%), white crystalline solid, mp 195–196 °C.

^1^H NMR (400 MHz, [D_6_]DMSO): δ = 12.95–12.50 (m, 2H, 2COOH), 7.67 (d, *J* = 5.0 Hz, 1H, H-thiophene), 7.15 (d, *J* = 5.0 Hz, 1H, H-thiophene), 5.50–4.90 (br.s, 1H, OH), 3.55 (d, *J* = 13.8 Hz, 1H, CH_2_), 3.21 (d, *J* = 13.8 Hz, 1H, CH_2_), 1.17 (s, 3H, CH_3_).

^13^C NMR (101 MHz, [D_6_]DMSO): δ = 177.3, 163.7, 144.4, 132.3, 129.7, 129.3, 73.7, 37.6, 24.6.

MS (ESI): *m*/*z* = 231.0 [M + H]^+^.

IR (ATR) ν_max_ = 3404 (OH), 1708 (C=O), 1672 (C=O) cm^−1^.

Anal. Calcd for C_9_H_10_O_5_S: C, 46.95; H, 4.38. Found: C, 46.86; H, 4.31.

**General Procedure for the Preparation of Methyl 3-((2-amino-4-methylthiazol-5-yl)methyl)thiophene-2-carboxylate 19a, Methyl 3-((2-aminothiazol-5-yl)methyl)thiophene-2-carboxylate 19b, and Methyl 3-((2-amino-4-methyl-1,3-selenazol-5-yl)methyl)thiophene-2-carboxylate 20a**

**Method A.** A mixture of the corresponding methyl 3-(2-bromo-3-oxobutyl)thiophene-2-carboxylate **4a** or methyl 3-(2-chloro-3-oxopropyl)thiophene-2-carboxylate **11** (3 mmol, 1.0 equiv.), thiourea (0.228 g, 3 mmol, 1.0 equiv.) or selenourea (0.369 g, 3 mmol, 1.0 equiv.) and 5 mL of methanol was stirred under reflux temperature over a period of 2 h. Then 40 mL of hot water was added to the mixture, the aqueous layer was separated and alkalified by a 20% solution of NH_3_ to pH ≈ 11. The mixture was allowed to cool to 20 °C whereupon a precipitate formed which was filtered off, dried on air and recrystallized from hexane–toluene.

**Methyl 3-((2-amino-4-methylthiazol-5-yl)methyl)thiophene-2-carboxylate 19a**.

Yield 0.648 g (80%), light-yellow solid, mp 178–179 °C.

^1^H NMR (400 MHz, CDCl_3_): δ = 7.38 (d, *J* = 5.1 Hz, 1H, H-thiophene), 6.89 (d, *J* = 5.1 Hz, 1H, H-thiophene), 5.14–4.59 (m, 2H, NH_2_), 4.29 (s, 2H, CH_2_), 3.88 (s, 3H, CH_3_), 2.17 (s, 3H, CH_3_).

^1^H NMR (500 MHz, [D_6_]DMSO): δ = 7.78 (d, *J* = 5.0 Hz, 1H, H-thiophene), 7.00 (d, *J* = 5.0 Hz, 1H, H-thiophene), 6.57 (s, 2H, NH_2_), 4.21 (s, 2H, CH_2_), 3.82 (s, 3H, CH_3_), 2.06 (s, 3H, CH_3_).

^13^C NMR (101 MHz, CDCl_3_): δ = 165.1 (o), 163.1 (o), 148.4 (o), 143.8 (o), 130.8 (+), 130.4 (+), 126.5 (o), 118.7 (o), 52.1 (+), 26.2 (−), 14.9 (+).

MS (ESI): *m*/*z* = 269.0 [M + H]^+^.

IR (ATR) ν_max_ = 3441 (NH), 3267 (NH), 1694 (C=O) cm^−1^.

Anal. Calcd for C_11_H_12_N_2_O_2_S_2_: C, 49.23; H, 4.51; N, 10.44. Found: C, 49.14; H, 4.45; N, 10.36.

**Methyl 3-((2-aminothiazol-5-yl)methyl)thiophene-2-carboxylate 19b**.

Yield 0.545 g (71%), light-yellow solid, mp 130–131 °C.

^1^H NMR (400 MHz, [D_6_]DMSO): δ = 7.80 (d, *J* = 5.1 Hz, 1H, H-thiophene), 7.07 (d, *J* = 5.1 Hz, 1H, H-thiophene), 6.70 (s, 1H, H-thiazole), 6.73–6.67 (br.s, 2H, NH_2_), 4.25 (s, 2H, CH_2_), 3.81 (s, 3H, CH_3_).

^13^C NMR (101 MHz, [D_6_]DMSO): δ = 168.1 (o), 162.2 (o), 148.6 (o), 135.7 (+), 132.1 (+), 131.1 (+), 125.6 (o), 123.0 (o), 51.9 (+), 26.2 (−).

MS (ESI): *m*/*z* = 255.0 [M + H]^+^.

IR (ATR) ν_max_ = 3407 (NH), 3295 (NH), 1633 (C=O) cm^−1^.

Anal. Calcd for C_10_H_10_N_2_O_2_S_2_: C, 47.23; H, 3.96; N, 11.02. Found: C, 47.16; H, 3.91; N, 10.93.

**Methyl 3-((2-amino-4-methyl-1,3-selenazol-5-yl)methyl)thiophene-2-carboxylate 20a**.

Yield 0.803 g (85%), light-yellow solid, mp 199–200 °C.

^1^H NMR (400 MHz, [D_6_]DMSO): δ = 7.78 (d, *J* = 5.1 Hz, 1H, H-thiophene), 7.03 (d, *J* = 5.1 Hz, 1H, H-thiophene), 6.87 (s, 2H, NH_2_), 4.23 (s, 2H, CH_2_), 3.81 (s, 3H, CH_3_), 2.04 (s, 3H, CH_3_).

^13^C NMR (101 MHz, [D_6_]DMSO): δ = 166.8 (o), 162.3 (o), 149.6 (o), 143.5 (o), 132.0 (+), 130.8 (+), 125.2 (o), 120.5 (o), 51.9 (+), 27.5 (−), 15.2 (+).

^77^Se NMR (76 MHz, [D_6_]DMSO): δ = 571.1.

MS (ESI): *m*/*z* = 317.0 [M + H]^+^.

IR (ATR) ν_max_ = 3435 (NH), 3263 (NH), 1690 (C=O) cm^−1^.

Anal. Calcd for C_11_H_12_N_2_O_2_SSe: C, 41.91; H, 3.84; N, 8.89. Found: C, 41.88; H, 3.76; N, 8.81.

**Procedure for the Preparation of Methyl 3-((2-amino-1,3-selenazol-5-yl)methyl)thiophene-2-carboxylate 20b**

**Method B.** A mixture of the methyl 3-(2-chloro-3-oxopropyl)thiophene-2-carboxylate **11** (0.699 g, 3 mmol, 1.0 equiv.), selenourea (0.369 g, 3 mmol, 1.0 equiv.), KI (48 mg, 0.3 mmol, 0.1 equiv.) and 5 mL of methanol was stirred under reflux temperature over a period of 2 h. Then 50 mL of hot water was added to the mixture, the aqueous layer was separated and alkalified by a 20% solution of NH_3_ to pH ≈ 11. The mixture was allowed to cool to 20 °C whereupon a precipitate formed which was filtered off, dried on air and recrystallized from hexane–toluene.

**Methyl 3-((2-amino-1,3-selenazol-5-yl)methyl)thiophene-2-carboxylate 20b**.

Yield 0.637 g (70%), light-yellow solid, mp 110–112 °C (decomp.).

^1^H NMR (400 MHz, [D_6_]DMSO): δ = 7.77 (d, *J* = 5.0 Hz, 1H, H-thiophene), 7.09 (d, *J* = 5.0 Hz, 1H, H-thiophene), 6.98–6.94 (br.s, 2H, NH_2_), 4.28 (s, 2H, CH_2_), 3.81 (s, 3H, CH_3_).

^13^C NMR (101 MHz, [D_6_]DMSO): δ = 170.4, 162.3, 149.3, 136.6, 132.0, 131.1, 128.4, 125.5, 52.0, 26.4.

MS (ESI): *m*/*z* = 303.0 [M + H]^+^.

IR (ATR) ν_max_ = 3103 (NH), 2949 (NH), 1698 (C=O) cm^−1^.

Anal. Calcd for C_10_H_10_N_2_O_2_SSe: C, 39.87; H, 3.35; N, 9.30. Found: C, 39.77; H, 3.30; N, 9.21.

**General Procedure for the Preparation of the Hydrobromides of Methyl 3-((2-imino-4-oxothiazolidin-5-yl)methyl)thiophene-2-carboxylate 21, Methyl 3-((2,4-diiminothiazolidin-5-yl)methyl)thiophene-2-carboxylate 22, Methyl 3-((2-imino-4-oxo-1,3-selenazolidin-5-yl)methyl)thiophene-2-carboxylate 23, and Methyl 3-((2,4-diimino-1,3-selenazolidin-5-yl)methyl)thiophene-2-carboxylate 24**.

A mixture of the corresponding methyl 3-(2-bromo-3-methoxy-3-oxopropyl)thiophene-2-carboxylate **5a** or methyl 3-(2-bromo-2-cyanoethyl)thiophene-2-carboxylate **6a** (3 mmol, 1.0 equiv.), thiourea (0.228 g, 3 mmol, 1.0 equiv.) or selenourea (0.369 g, 3 mmol, 1.0 equiv.) and 3 mL of methanol was stirred under reflux temperature over a period of 3 h. The mixture was allowed to cool to 20 °C whereupon a precipitate of product was formed which was filtered off and recrystallized from methanol-MTBE.

**Methyl 3-((2-imino-4-oxothiazolidin-5-yl)methyl)thiophene-2-carboxylate hydrobromide 21**.

Yield 0.846 g (80%), white crystalline solid, mp 162–163 °C.

^1^H NMR (400 MHz, [D_6_]DMSO): δ = 9.31–9.02 (m, 2H, NH), 7.81 (d, *J* = 5.1 Hz, 1H, H-thiophene), 7.08 (d, *J* = 5.1 Hz, 1H, H-thiophene), 4.69 (dd, *J* = 8.8, 5.0 Hz, 1H, CH), 3.80–3.73 (m, 4H, CH_3_, CH_2_), 3.35 (dd, *J* = 14.1, 8.9 Hz, 1H, CH_2_).

^13^C NMR (101 MHz, [D_6_]DMSO): δ = 186.5, 180.2, 162.3, 145.9, 132.1, 130.9, 127.4, 55.5, 52.1, 31.7.

MS (ESI): *m*/*z* = 271.0 [M + H]^+^.

IR (ATR) ν_max_ = 3368 (C=O), 3266 (C=O), 1604 (C=O) cm^−1^.

Anal. Calcd for C_10_H_11_BrN_2_O_3_S_2_: C, 34.20; H, 3.16; N, 7.98. Found: C, 34.11; H, 3.07; N, 7.91.

**Methyl 3-((2,4-diiminothiazolidin-5-yl)methyl)thiophene-2-carboxylate hydrobromide 22**.

Yield 0.749 g (71%), white crystalline solid, mp 245–246 °C.

^1^H NMR (400 MHz, [D_6_]DMSO): δ = 10.41–10.19 (br.s, 2H, NH), 10.16–10.04 (br.s, 1H, NH), 10.01–9.83 (br.s, 1H, NH), 7.91 (d, *J* = 5.0 Hz, 1H, H-thiophene), 7.18 (d, *J* = 5.0 Hz, 1H, H-thiophene), 5.59 (dd, *J* = 8.9, 4.5 Hz, 1H, CH), 3.92 (dd, *J* = 14.4, 4.3 Hz, 1H, CH_2_), 3.80 (s, 3H, CH_3_), 3.70 (dd, *J* = 14.3, 9.3 Hz, 2H, CH_2_).

^13^C NMR (101 MHz, [D_6_]DMSO): δ = 184.2, 162.1, 143.9, 132.6, 130.2, 127.9, 56.6, 52.2, 31.9.

MS (ESI): *m*/*z* = 270.0 [M + H]^+^.

IR (ATR) ν_max_ = 3198 (NH), 2989 (NH), 1641 (C=O) cm^−1^.

Anal. Calcd for C_10_H_12_BrN_3_O_2_S_2_: C, 34.29; H, 3.45; N, 12.00. Found: C, 34.18; H, 3.40; N, 11.91.

**Methyl 3-((2-imino-4-oxo-1,3-selenazolidin-5-yl)methyl)thiophene-2-carboxylate hydrobromide 23**.

Yield 1.015 g (85%), white crystalline solid, mp 212–213 °C.

^1^H NMR (400 MHz, [D_6_]DMSO): δ = 9.13–8.83 (m, 2H, NH), 7.81 (d, *J* = 5.1 Hz, 1H, H-thiophene), 7.10 (d, *J* = 5.1 Hz, 1H, H-thiophene), 4.95 (dd, *J* = 9.2, 4.9 Hz, 1H, CH), 3.87 (dd, *J* = 14.3, 5.0 Hz, 1H, CH_2_), 3.79 (s, 3H, CH_3_), 3.45 (dd, *J* = 14.3, 9.2 Hz, 1H, CH_2_).

^13^C NMR (101 MHz, [D_6_]DMSO): δ = 188.9, 175.7, 162.2, 147.2, 132.0, 130.6, 126.9, 55.3, 52.0, 32.3.

MS (ESI): *m*/*z* = 319.0 [M + H]^+^.

IR (ATR) ν_max_ = 3293 (NH), 2928 (NH), 1701 (C=O), 1648 (C=O) cm^−1^.

Anal. Calcd for C_10_H_11_BrN_2_O_3_SSe: C, 30.17; H, 2.78; N, 7.04. Found: C, 30.08; H, 2.75; N, 6.93.

**Methyl 3-((2,4-diimino-1,3-selenazolidin-5-yl)methyl)thiophene-2-carboxylate hydrobromide 24**.

Yield 0.695 g (73%), white crystalline solid, mp 246–247 °C.

^1^H NMR (400 MHz, [D_6_]DMSO): δ = 10.37–9.88 (br.s, 4H), 7.89 (d, *J* = 5.1 Hz, 1H, H-thiophene), 7.17 (d, *J* = 5.1 Hz, 1H, H-thiophene), 5.69 (dd, *J* = 9.3, 4.7 Hz, 1H, CH), 3.97 (dd, *J* = 14.6, 4.8 Hz, 1H, CH_2_), 3.80 (s, 3H, CH_3_), 3.8–3.73 (m, 1H, CH_2_).

^13^C NMR (101 MHz, [D_6_]DMSO): δ = 185.1, 182.0, 162.2, 145.2, 132.6, 130.1, 127.7, 54.1, 52.2, 32.8.

MS (ESI): *m*/*z* = 318.0 [M + H]^+^.

IR (ATR) ν_max_ = 3200 (NH), 1710 (C=O)cm^−1^.

Anal. Calcd for C_10_H_12_BrN_3_O_2_SSe: C, 30.24; H, 3.05; N, 10.58. Found: C, 30.15; H, 2.97; N, 10.51.

**General Procedure for the Preparation of Dimethyl 4-amino-[2,3′-bithiophene]-2′,5-dicarboxylate 26a and Methyl 4-amino-5-(phenylcarbamoyl)-[2,3′-bithiophene]-2′-carboxylate 26b**.

A solution of methyl 3-(2-bromo-2-chloro-2-cyanoethyl)thiophene-2-carboxylate **7a** (3 mmol, 1.0 equiv.) in methanol (5 mL) was added dropwise under stirring to a mixture of the appropriate thiol **25** (methyl thioglycolate **25a**, mercaptoacetanilide **25b**, or mercaptoacetone **25c**) (3.03 mmol, 1.01 equiv.) with a solution of MeONa (9 mL, 1M) in methanol at ambient temperature. The resulting mixture was stirred for 2 h at ambient temperature, then under reflux over 1 h. The solvent was evaporated under reduced pressure. Acetic acid (20 mL, ω = 2%) was added to residue. The product was filtered off and recrystallized from methanol.

In the case of the reaction with mercaptoacetone **25c**, the reaction mixture was extracted after acidification with MTBE (3 × 5 mL). The organic phase was separated, dried over Na_2_SO_4_, and then 37% aqueous HCl (0.25 mL, 3 mmol, 1 equiv.) was added. The resulting precipitate of **26c** was filtered off and recrystallized from methanol-MTBE.

**Dimethyl 4-amino-[2,3′-bithiophene]-2′,5-dicarboxylate 26a**.

Yield 0.670 g (75%), white solid, mp 128–129 °C.

^1^H NMR (400 MHz, [D_6_]DMSO): δ = 7.94 (d, *J* = 5.2 Hz, 1H, H-thiophene’), 7.35 (d, *J* = 5.2 Hz, 1H, H-thiophene’), 7.12 (s, 1H, 4H-thiophene), 6.58 (s, 2H, NH_2_), 3.80 (s, 3H, CH_3_), 3.73 (s, 3H, CH_3_).

^13^C NMR (101 MHz, [D_6_]DMSO): δ = 163.9, 161.4, 154.6, 139.9, 138.4, 132.4, 130.8, 126.9, 121.6, 98.0, 52.3, 51.0.

MS (ESI): *m*/*z* = 298.0 [M + H]^+^.

IR (ATR) ν_max_ = 1670 (C=O) cm^−1^.

Anal. Calcd for C_12_H_11_NO_4_S_2_: C, 48.47; H, 3.73; N, 4.71. Found: C, 48.42; H, 3.65; N, 4.62.

**Methyl 4-amino-5-(phenylcarbamoyl)-[2,3′-bithiophene]-2′-carboxylate 26b**.

Yield 0.736 g (71%), white solid, mp 218–219 °C.

^1^H NMR (400 MHz, [D_6_]DMSO): δ = 13.65–12.84 (m, 1H, COOH), 9.32 (s, 1H, NH), 7.89 (d, *J* = 5.2 Hz, 1H, H-thiophene’), 7.67 (dd, *J* = 8.6, 1.0 Hz, 2H, C_6_H_5_), 7.31 (d, *J* = 5.2 Hz, 1H, H-thiophene’), 7.32–7.27 (m, 2H, C_6_H_5_), 7.15 (s, 1H, H-thiophene), 7.03 (t, *J* = 7.4 Hz, 1H, C_6_H_5_), 6.76–6.57 (m, 2H, NH_2_).

^13^C NMR (101 MHz, [D_6_]DMSO): δ = 163.1, 162.5, 154.1, 139.2, 137.8, 137.4, 131.6, 130.7, 129.0, 128.4, 123.0, 122.2, 120.8, 101.4.

MS (ESI): *m*/*z* = 359.0 [M + H]^+^.

IR (ATR) ν_max_ = 3484 (NH), 3440 (NH), 1667 (C=O), 1648 (C=O) cm^−1^.

Anal. Calcd for C_17_H_14_N_2_O_3_S_2_: C, 56.97; H, 3.94; N, 7.82. Found: C, 56.89; H, 3.90; N, 7.73.

**Methyl 5-acetyl-4-amino-[2,3′-bithiophene]-2′-carboxylate hydrochloride 26c**.

Yield 0.660 g (69%), white crystalline solid, mp 115–116 °C.

^1^H NMR (400 MHz, [D_6_]DMSO): δ = 7.96 (d, *J* = 5.2 Hz, 1H, H-thiophene), 7.36 (d, *J* = 5.2 Hz, 1H, H-thiophene), 7.11 (s, 1H, H-thiophene’), 4.07–3.67 (br.s, 3H, NH_3_), 3.80 (s, 3H, CH_3_), 2.27 (s, 3H, CH_3_).

^13^C NMR (101 MHz, [D_6_]DMSO): δ = 189.6, 161.5, 154.5, 140.1, 138.3, 132.6, 130.9, 127.3, 121.8, 109.7, 52.4, 28.4.

MS (ESI): *m*/*z* = 282.0 [M + H]^+^.

IR (ATR) ν_max_ = 3437 (NH), 3344 (NH), 1727(C=O) cm^−1^.

Anal. Calcd for C_12_H_12_ClNO_3_S_2_: C, 45.35; H, 3.81; N, 4.41. Found: C, 45.24; H, 3.77; N, 4.33.

**General Procedure for the Preparation of Dimethyl 4-hydroxy-[2,3′-bithiophene]-2′,5-dicarboxylate 27a, Methyl 4-hydroxy-5-(phenyl- carbamoyl)-[2,3′-bithiophene]-2′-carboxylate 27b, and Methyl 5-acetyl-4-hydroxy- [2,3′-bithiophene]-2′-carboxylate 27c.**

A solution of methyl 3-(2-bromo-2-chloro-3-methoxy-3-oxopropyl)thiophene-2-carboxylate **8a** (3 mmol, 1.0 equiv.) in methanol (5 mL) was added dropwise under stirring to a mixture of appropriate thiol **25** (methyl thioglycolate **25a**, mercaptoacetanilide **25b**, or mercaptoacetone **25c**) (3.03 mmol, 1.01 equiv.) with a solution of MeONa (9 mL, 1 M) in methanol at ambient temperature. The resulting mixture was stirred for 2 h at ambient temperature, then under reflux over 1 h. The solvent was then evaporated under reduced pressure. Acetic acid (20 mL, ω = 2%) was added to the residue. The product was filtered off and recrystallized from methanol.

**Dimethyl 4-hydroxy-[2,3′-bithiophene]-2′,5-dicarboxylate 27a**.

Yield 0.670 g (74%), white crystalline solid, mp 113–114 °C.

^1^H NMR (400 MHz, [D_6_]DMSO): δ = 10.65–10.17 (br.s, 1H, OH), 7.96 (d, *J* = 5.1 Hz, 1H, H-thiophene’), 7.42 (d, *J* = 5.2 Hz, 1H, H-thiophene’), 7.30 (s, 1H, 3H-thiophene), 3.80 (s, 3H, CH_3_), 3.77 (s, 3H, CH_3_).

^13^C NMR (101 MHz, [D_6_]DMSO): δ = 162.7, 161.4, 160.1, 139.3, 138.0, 132.5, 130.7, 126.9, 122.0, 105.7, 52.4, 51.5.

MS (ESI): *m*/*z* = 299.0 [M + H]^+^.

IR (ATR) ν_max_ = 3331 (OH), 1715 (C=O), 1675 (C=O) cm^−1^.

Anal. Calcd for C_12_H_10_O_5_S_2_: C, 48.31; H, 3.38. Found: C, 48.21; H, 3.36.

**Methyl 4-hydroxy-5-(phenylcarbamoyl)-[2,3′-bithiophene]-2′-carboxylate 27b**.

Yield 0.862 g (80%), white solid, mp 213–214 °C.

^1^H NMR (400 MHz, [D_6_]DMSO): δ = 12.40–11.80 (br.s, 1H, OH), 9.55 (s, 1H, NH), 7.97 (d, *J* = 5.0 Hz, 1H, H-thiophene’), 7.66 (d, *J* = 7.4 Hz, 2H), 7.44–7.33 (m, 4H), 7.10 (t, *J* = 7.4 Hz, 1H, C_6_H_5_), 3.82 (s, 3H, CH_3_).

^13^C NMR (101 MHz, [D_6_]DMSO): δ = 161.5, 160.2, 155.7, 138.4, 138.3, 137.8, 132.6, 130.7, 128.9, 126.5, 123.6, 121.6, 119.8, 113.1, 52.4.

MS (ESI): *m*/*z* = 360.0 [M + H]^+^.

IR (ATR) ν_max_ = 3315 (NH), 1711 (C=O), 1640 (C=O) cm^−1^.

Anal. Calcd for C_17_H_13_NO_4_S_2_: C, 56.81; H, 3.65; N, 3.90. Found: C, 56.74; H, 3.60; N, 3.81.

**Methyl 5-acetyl-4-hydroxy-[2,3′-bithiophene]-2′-carboxylate 27c**.

Yield 0.716 g (84%), white crystalline solid, mp 122–123 °C.

^1^H NMR (400 MHz, [D_6_]DMSO): δ = 11.82–11.18 (m, 1H, OH), 7.96 (d, *J* = 5.0 Hz, 1H, H-thiophene), 7.39 (d, *J* = 4.9 Hz, 1H, H-thiophene), 7.33 (s, 1H, 1H, H-thiophene’), 3.81 (s, 3H, CH_3_), 2.47 (s, 3H, CH_3_).

^13^C NMR (101 MHz, [D_6_]DMSO): δ = 190.3, 161.4, 159.2, 140.7, 138.2, 132.6, 130.7, 127.1, 122.2, 119.4, 52.4, 28.7.

MS (ESI): *m*/*z* = 283.0 [M + H]^+^.

IR (ATR) ν_max_ = 3104 (OH), 1710 (C=O), 1614 (C=O) cm^−1^.

Anal. Calcd for C_12_H_10_O_4_S_2_: C, 51.05; H, 3.57. Found: C, 50.94; H, 3.51.

## Data Availability

The data are contained within the article; further inquiries can be directed to the corresponding authors.

## Supplementary Materials

The following supporting information can be downloaded at https://www.mdpi.com/article/10.3390/molecules30183758/s1; Figure S1: ^1^H-NMR spectrum of **4a**; Figure S2: ^13^C-NMR spectrum of **4a**; Figure S3: ^1^H-NMR spectrum of **5a**; Figure S4: ^1^H-NMR spectrum of **5a**; Figure S5: ^13^C-NMR spectrum of **5a**; Figure S6: ^13^C-NMR spectrum of **5a**; Figure S7: ^1^H-NMR spectrum of **6a**; Figure S8: ^13^C-NMR spectrum of **6a**; Figure S9: ^1^H-NMR spectrum of **7a**; Figure S10: ^1^H-NMR spectrum of **7a**; Figure S11: ^13^C-NMR spectrum of **7a**; Figure S12: ^13^C-NMR spectrum of **7a**; Figure S13: ^1^H-NMR spectrum of **8a**; Figure S14: ^13^C-NMR spectrum of **8a**; Figure S15: ^1^H-NMR spectrum of **9**; Figure S16: ^13^C-NMR spectrum of **9**; Figure S17: ^1^H-NMR spectrum of **4b**; Figure S18: ^13^C-NMR spectrum of **4b**; Figure S19: ^1^H-NMR spectrum of **5b**; Figure S20: ^13^C-NMR spectrum of **5b**; Figure S21: ^1^H-NMR spectrum of **8b**; Figure S22: ^13^C-NMR spectrum of **8b**; Figure S23: ^1^H-NMR spectrum of **11**; Figure S24: ^13^C-NMR spectrum of **11**; Figure S25: ^1^H-NMR spectrum of **12**; Figure S26: ^13^C-NMR spectrum of **12**; Figure S27: ^1^H-NMR spectrum of **13**; Figure S28: ^13^C-NMR spectrum of **13**; Figure S29: ^1^H-NMR spectrum of **14a**; Figure S30: ^13^C-NMR spectrum of **14a**; Figure S31: ^1^H-NMR spectrum of **14b**; Figure S32: ^13^C-NMR spectrum of **14b**; Figure S33: ^1^H-NMR spectrum of **15**; Figure S34: ^13^C-NMR spectrum of **15**; Figure S35: ^1^H-NMR spectrum of **16**; Figure S36: ^13^C-NMR spectrum of **16**; Figure S37: ^1^H-NMR spectrum of **17**; Figure S38: ^13^C-NMR spectrum of **17**; Figure S39: ^1^H-NMR spectrum of **18**; Figure S40: ^13^C-NMR spectrum of **18**; Figure S41: ^1^H-NMR spectrum of **19a**; Figure S42: ^1^H-NMR spectrum of **19a**; Figure S43: ^13^C-NMR spectrum of **19a**; Figure S44: ^1^H-NMR spectrum of **19b**; Figure S45: ^13^C-NMR spectrum of **19b**; Figure S46: ^1^H-NMR spectrum of **20a**; Figure S47: ^13^C-NMR spectrum of **20a**; Figure S48: ^77^Se-NMR spectrum of **20a**; Figure S49: ^1^H-NMR spectrum of **20b**; Figure S50: ^13^C-NMR spectrum of **20b**; Figure S51: ^1^H-NMR spectrum of **21**; Figure S52: ^13^C-NMR spectrum of **21**; Figure S53: ^1^H-NMR spectrum of **22**; Figure S54: ^13^C-NMR spectrum of **22**; Figure S55: ^1^H-NMR spectrum of **23**; Figure S56: ^13^C-NMR spectrum of **23**; Figure S57: ^1^H-NMR spectrum of **24**; Figure S58: ^13^C-NMR spectrum of **24**; Figure S59: ^1^H-NMR spectrum of **26a**; Figure S60: ^13^C-NMR spectrum of **26a**; Figure S61: ^1^H-NMR spectrum of **26b**; Figure S62: ^13^C-NMR spectrum of **26b**; Figure S63: ^1^H-NMR spectrum of **26c**; Figure S64: ^13^C-NMR spectrum of **26c**; Figure S65: ^1^H-NMR spectrum of **27a**; Figure S66: ^13^C-NMR spectrum of **27a**; Figure S67: ^1^H-NMR spectrum of **27b**; Figure S68: ^13^C-NMR spectrum of **27b**; Figure S69: ^1^H-NMR spectrum of **27c**; Figure S70: ^13^C-NMR spectrum of **27c.**
