# Supplementary material for: 2-(Methoxycarbonyl)thiophen-3-yl-diazonium Salts: Efficient Precursors for the Formation of C–C Bonds in Thiophene-Containing Heterocyclic Systems
Source: Molecules. 2025 Sep 16;30(18):3758. doi: 10.3390/molecules30183758 (PMC12472641; doi:10.3390/molecules30183758)

Supporting Information

2-(Methoxycarbonyl)thiophen-3-yl-diazonium salts: Efficient precursors for the formation of C-C bonds in thiophene-containing heterocyclic systems

Yurii V. Ostapiuk,[a]* Oksana V. Barabash,[a] Mary Y. Ostapiuk,[a] Mykola Kravets,[b] Andreas Schmidt[c]*, Mykola D. Obushak[a]

[a] Ivan Franko National University of Lviv, Department of Organic Chemistry, Kyryla i Mefodiya Str. 6, 79005 Lviv, Ukraine
E-mail: y.ostapiuk@gmail.com, yurii.ostapiuk@lnu.edu.ua

[b] Institute of Physical Chemistry of the Polish Academy of Sciences, Kasprzaka 44/52, 01-224 Warsaw, Poland.

[c] Clausthal University of Technology, Institute of Organic Chemistry, Leibnizstrasse 6, D-38678 Clausthal-Zellerfeld, Germany.
E-mail: schmidt@ioc.tu-clausthal.de

Contents

^{1}H NMR and ^{13}C NMR spectra of compounds……………………………………………………………………………………..S1

^{1}H NMR (400 MHz, [D$_6$]DMSO)

Methyl 3-(2-bromo-3-oxobutyl)thiophene-2-carboxylate 4a.

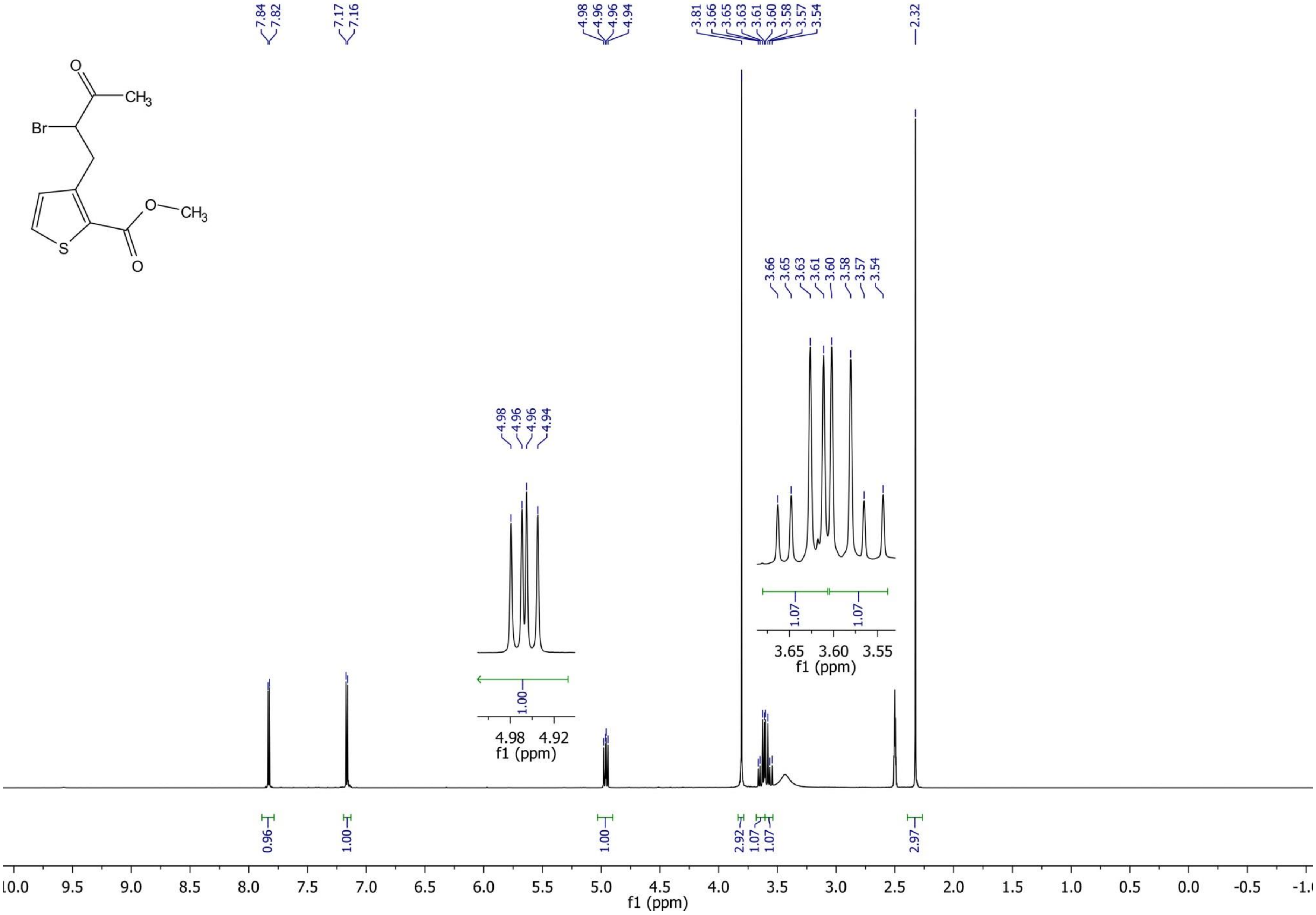

Methyl 3-(2-bromo-3-oxobutyl)thiophene-2-carboxylate 4a.

^{13}C NMR (101 MHz, [D_6]DMSO)

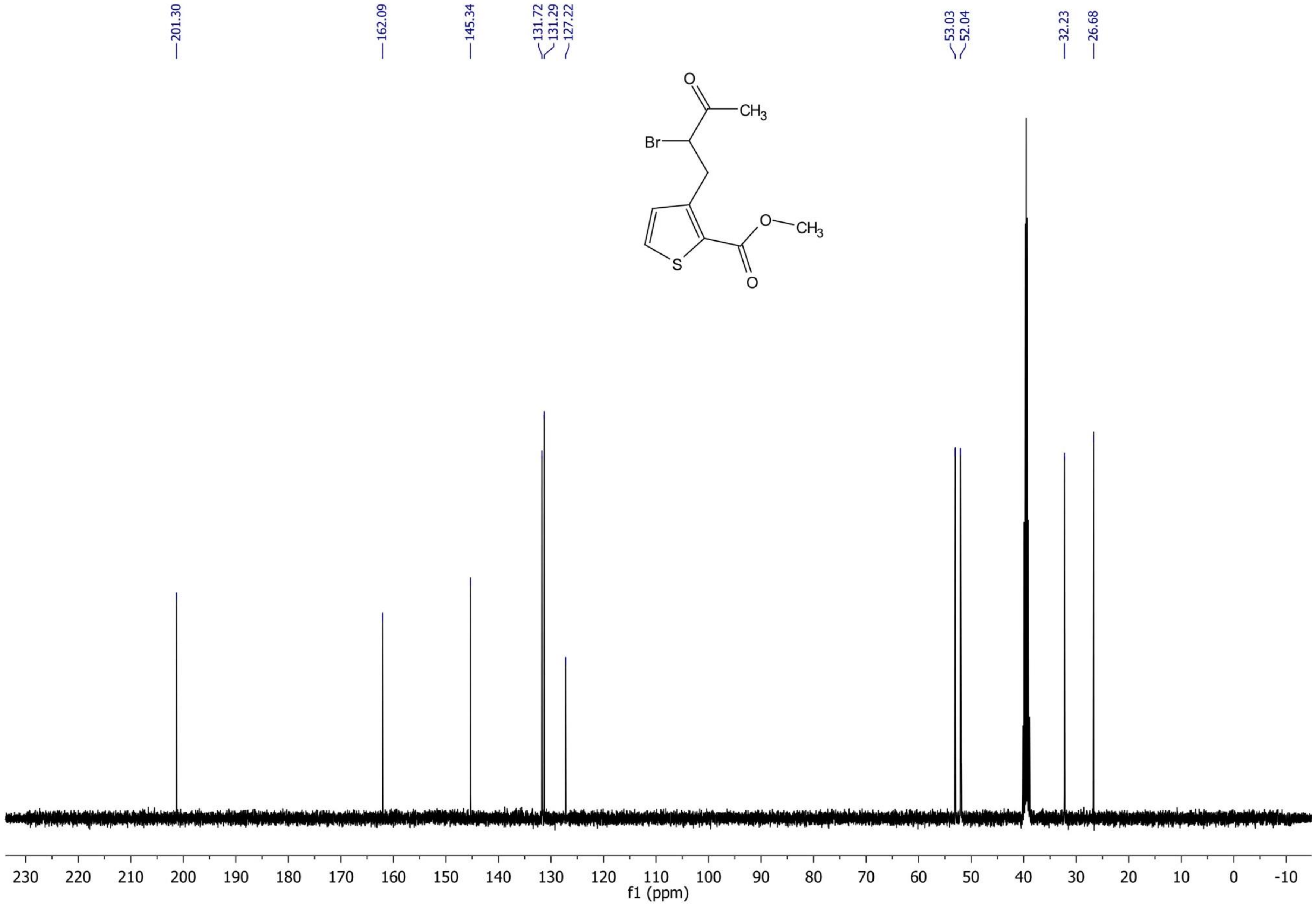

Methyl 3-(2-bromo-3-methoxy-3-oxopropyl)thiophene-2-carboxylate 5a.

^{1}H NMR (400 MHz, [D$_6$]DMSO)

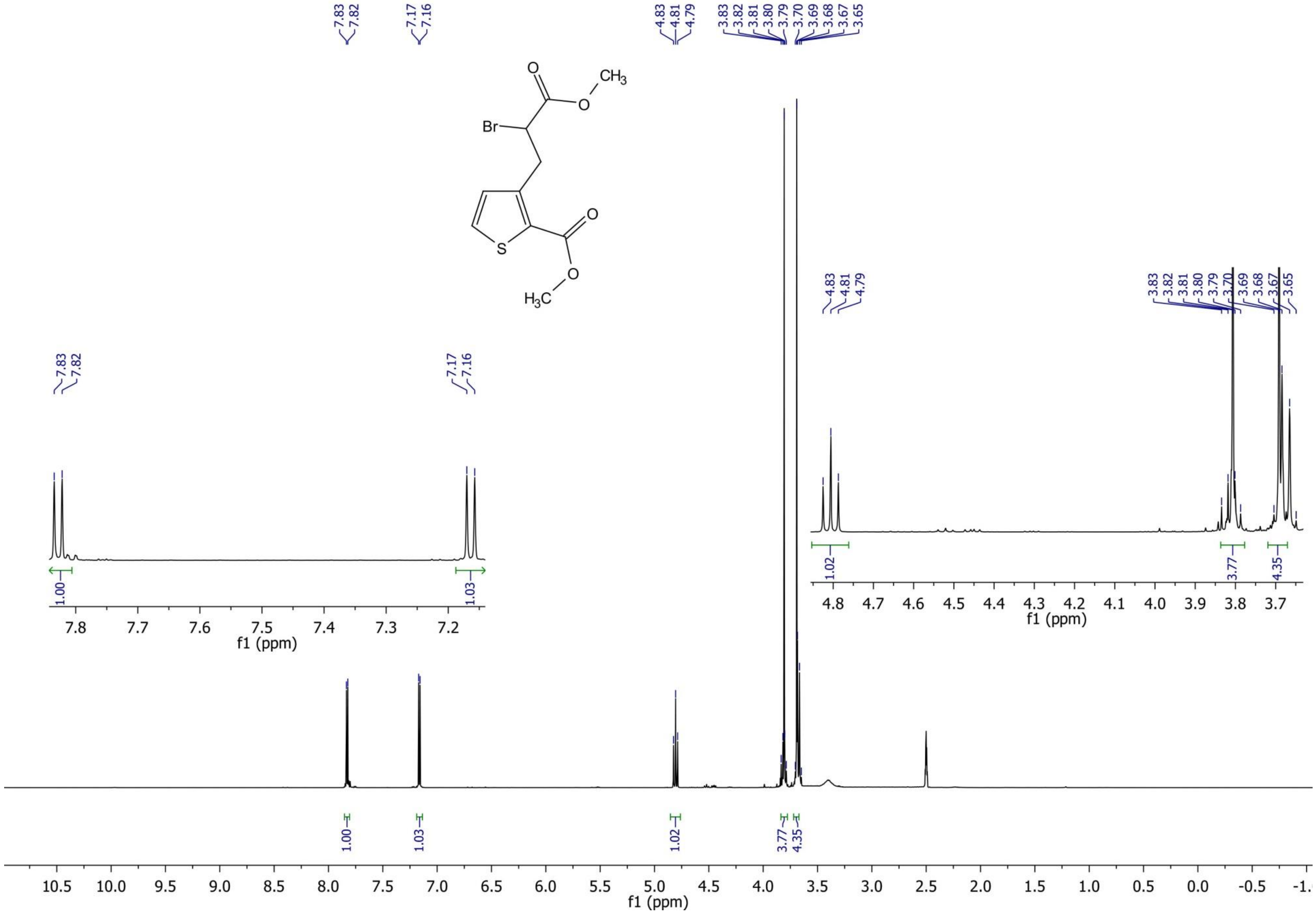

Methyl 3-(2-bromo-3-methoxy-3-oxopropyl)thiophene-2-carboxylate 5a.

^{1}H NMR (500 MHz, $CDCl_3$)

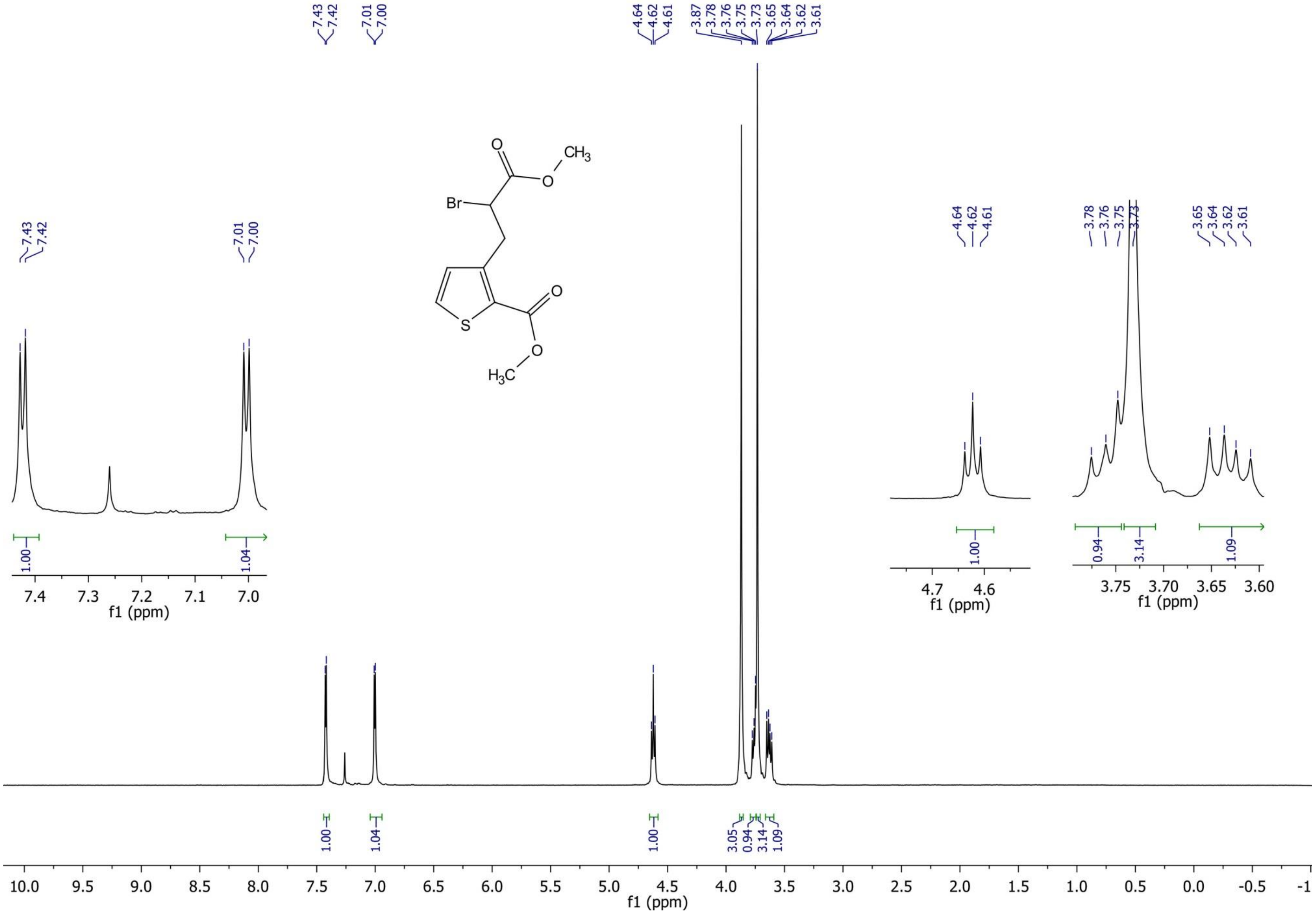

Methyl 3-(2-bromo-3-methoxy-3-oxopropyl)thiophene-2-carboxylate 5a.

^{13}C NMR (101 MHz, [D$_6$]DMSO)

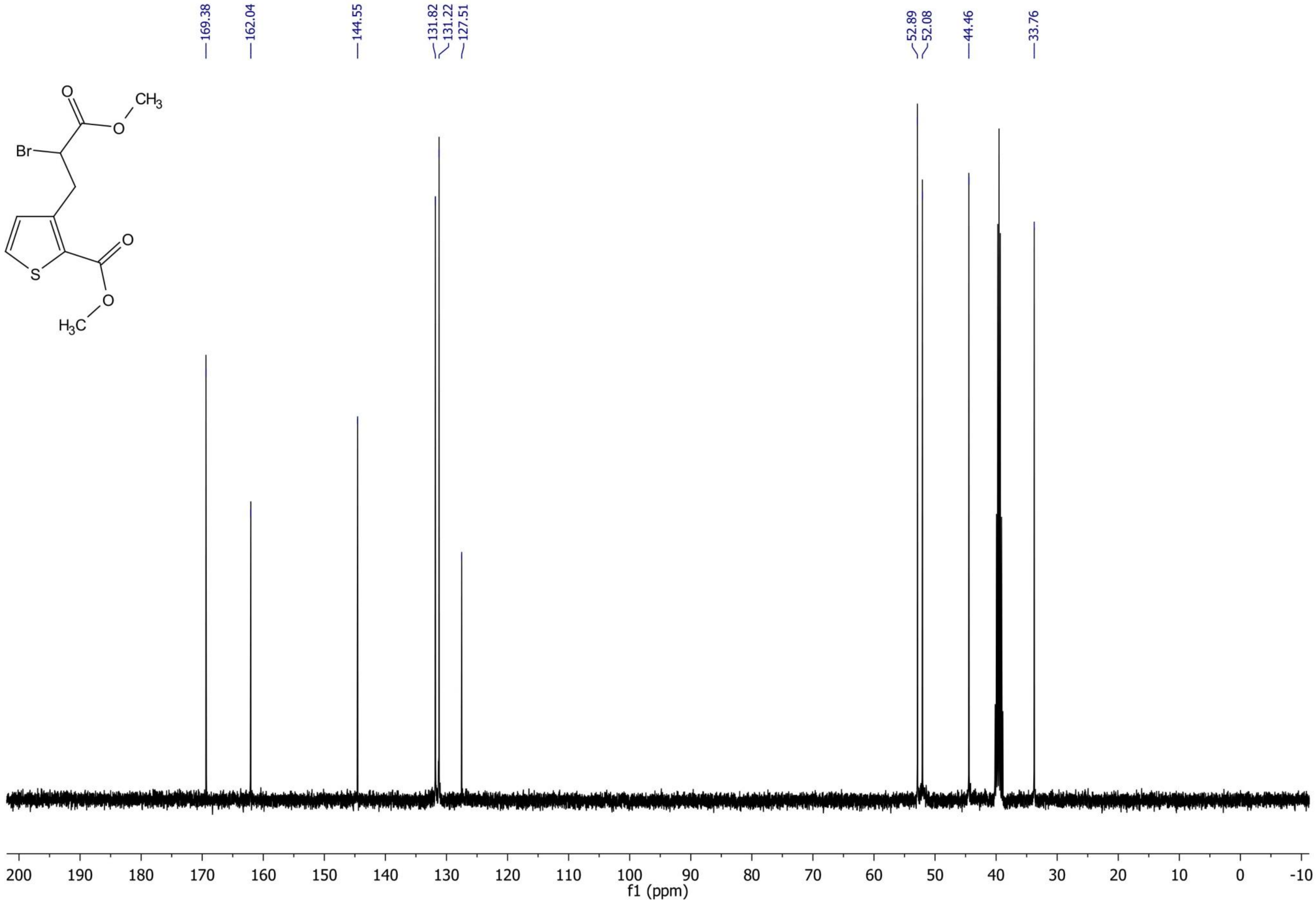

Methyl 3-(2-bromo-3-methoxy-3-oxopropyl)thiophene-2-carboxylate 5a.

^{13}C NMR (126 MHz, $CDCl_3$)

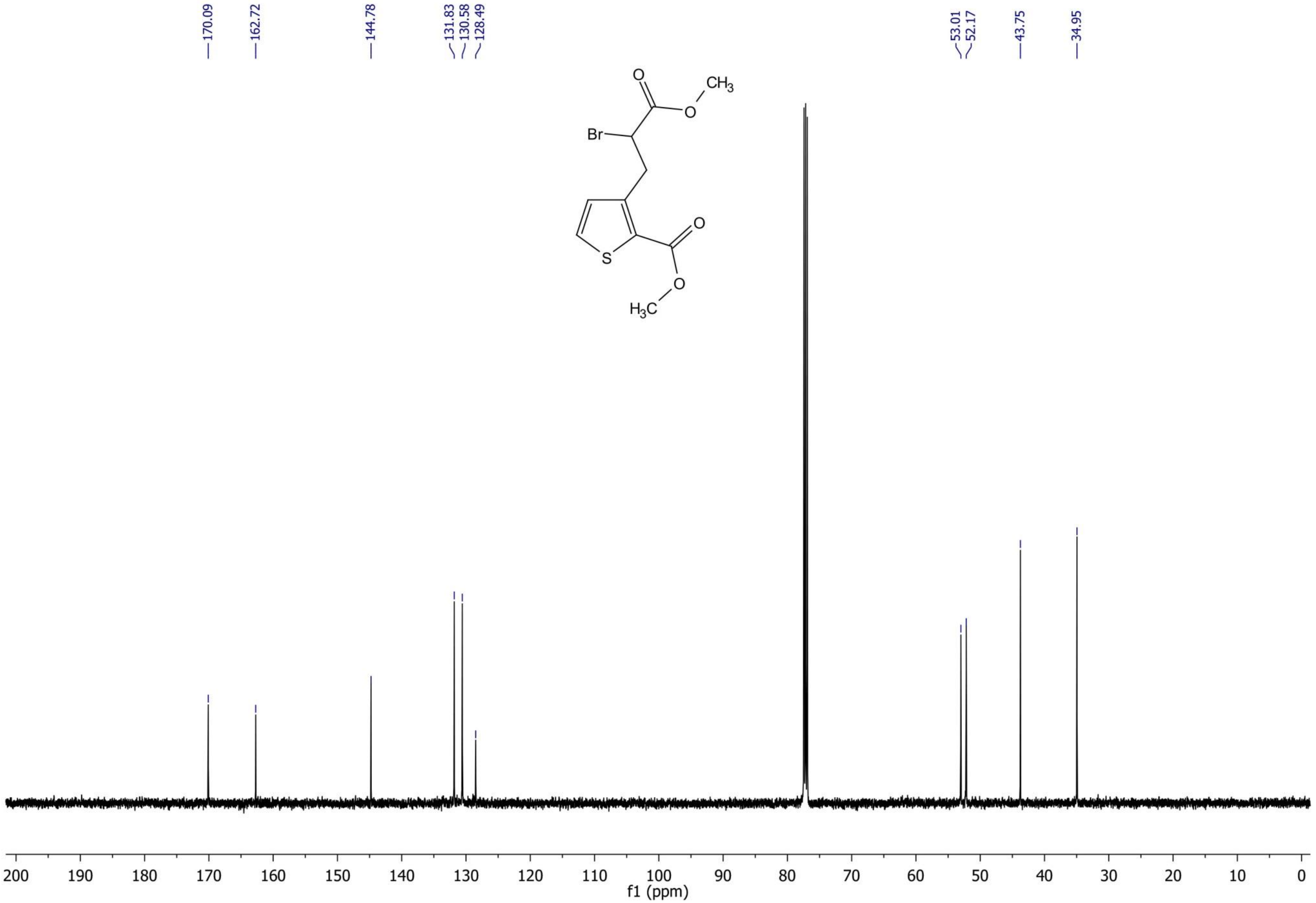

Methyl 3-(2-bromo-2-cyanoethyl)thiophene-2-carboxylate 6a.

^{1}H NMR (400 MHz, [D$_6$]DMSO)

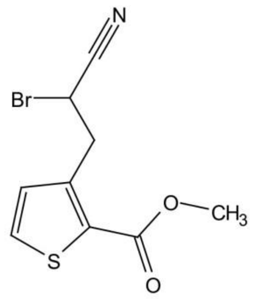

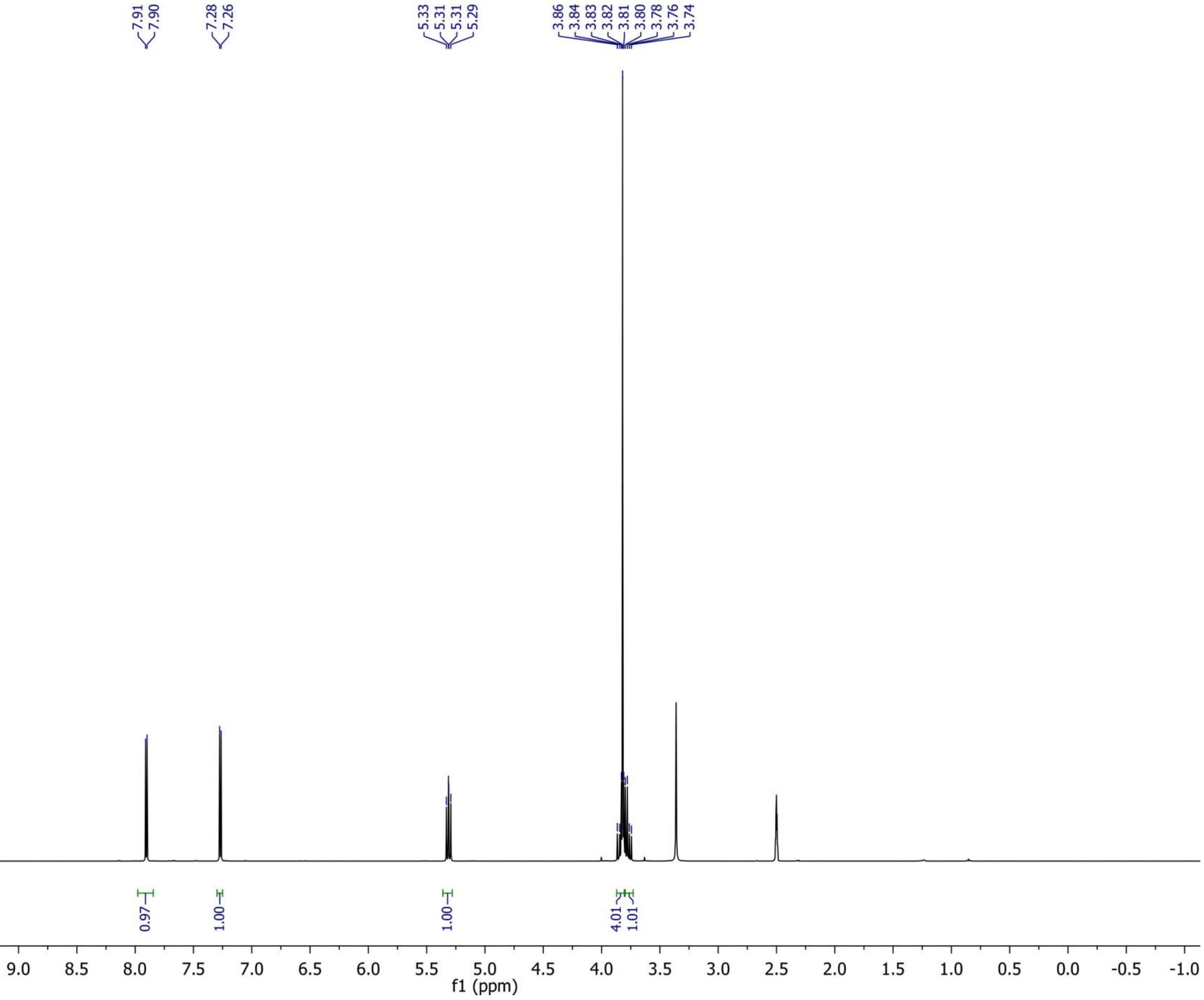

Methyl 3-(2-bromo-2-cyanoethyl)thiophene-2-carboxylate 6a.

^{13}C NMR (101 MHz, [D$_6$]DMSO)

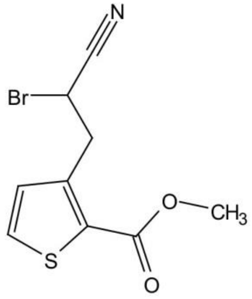

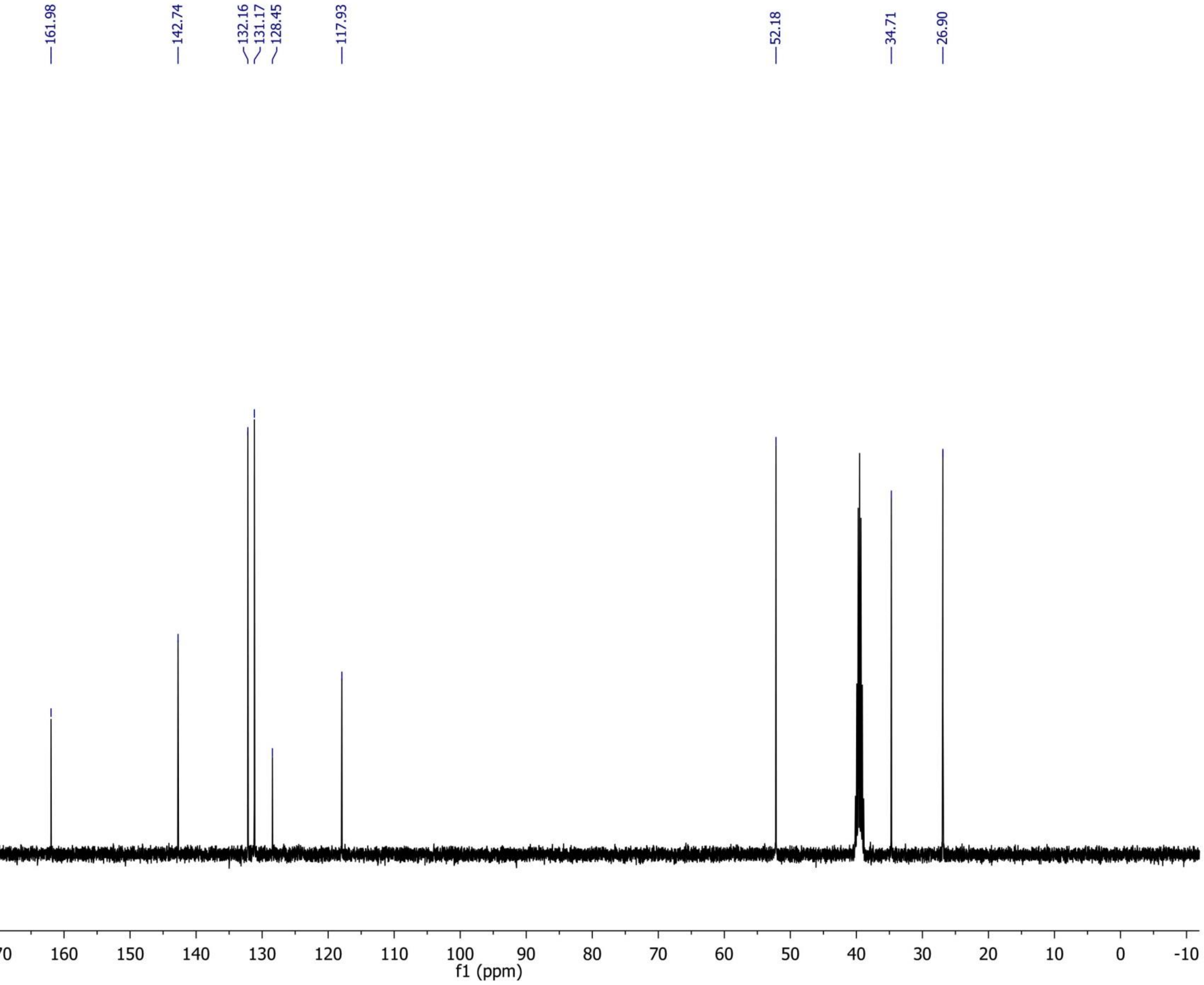

Methyl 3-(2-bromo-2-chloro-2-cyanoethyl)thiophene-2-carboxylate 7a.

^{1}H NMR (400 MHz, [D$_6$]DMSO)

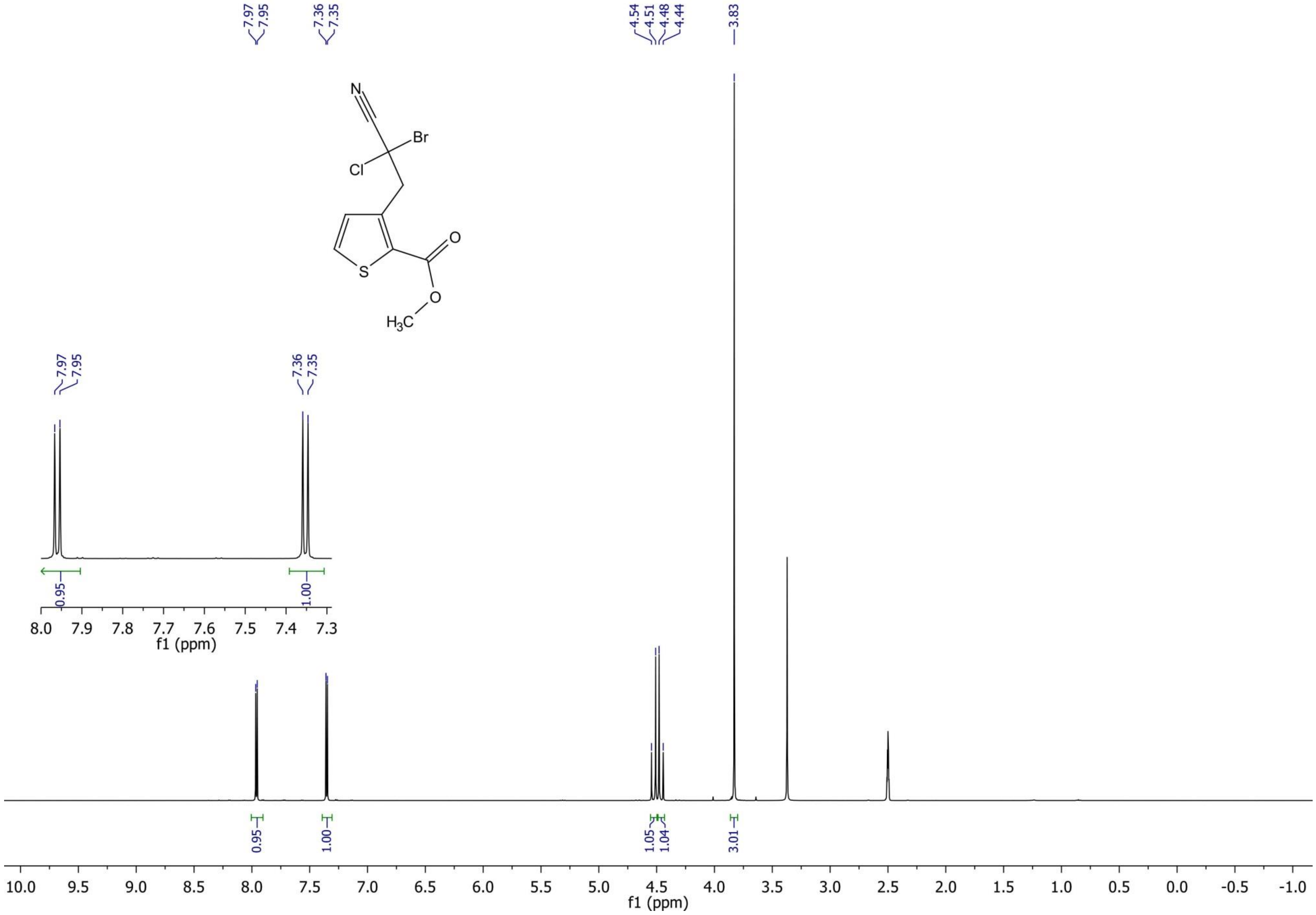

Methyl 3-(2-bromo-2-chloro-2-cyanoethyl)thiophene-2-carboxylate 7a.

^{1}H NMR (400 MHz, CD_3OD)

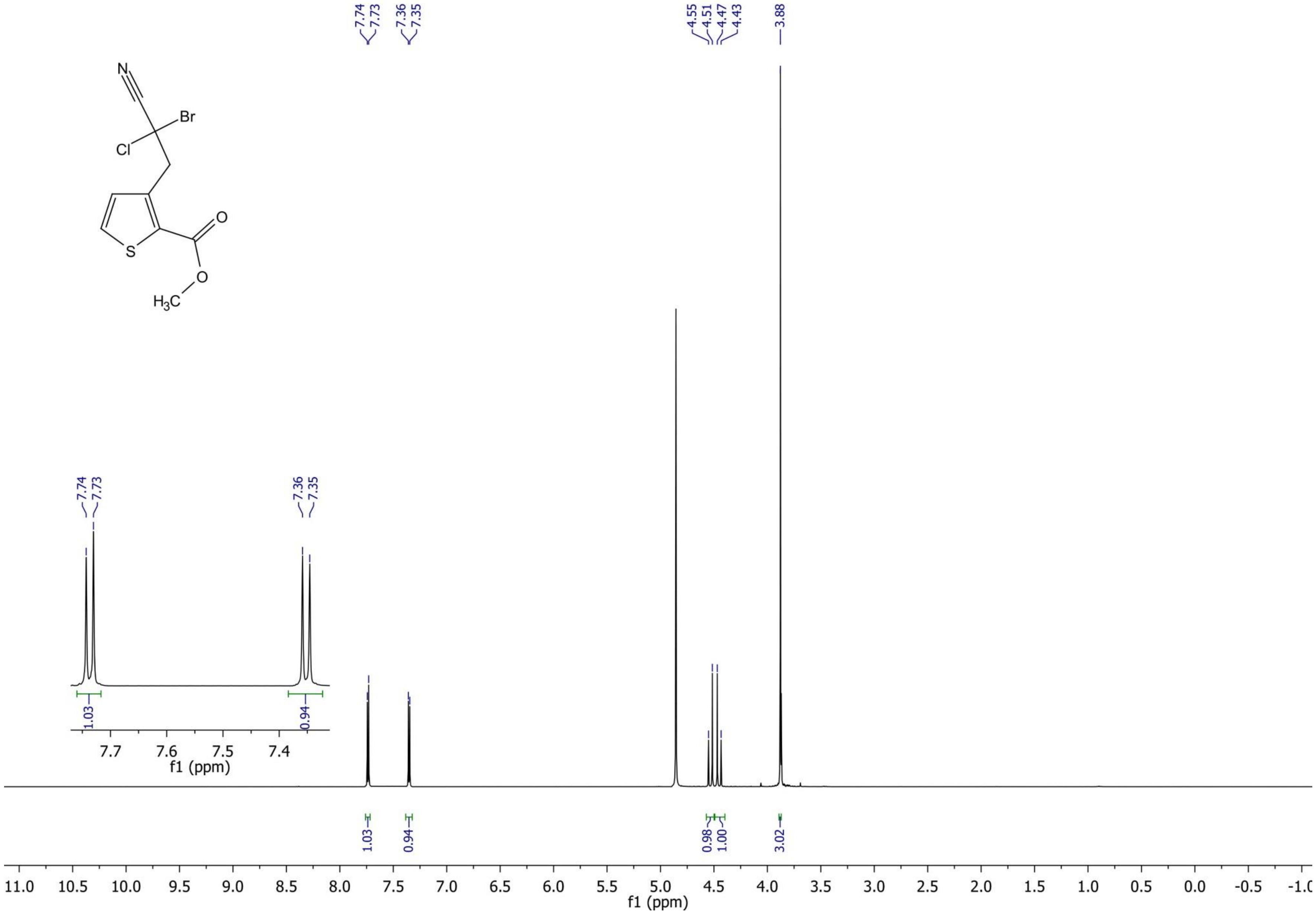

Methyl 3-(2-bromo-2-chloro-2-cyanoethyl)thiophene-2-carboxylate 7a.

^{13}C NMR (101 MHz, [D_6]DMSO)

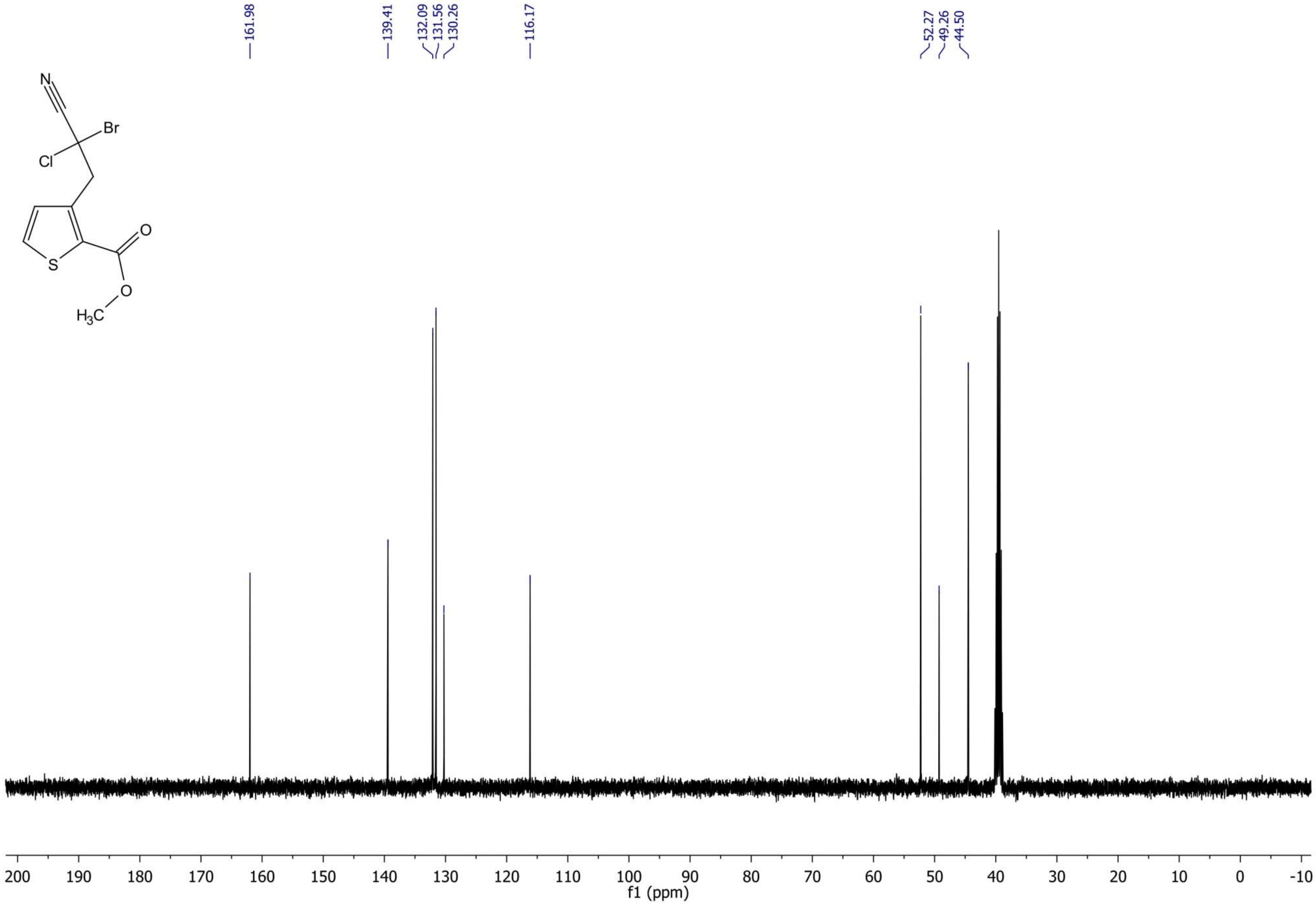

Methyl 3-(2-bromo-2-chloro-2-cyanoethyl)thiophene-2-carboxylate 7a.

^{13}C NMR (101 MHz, CD_3OD)

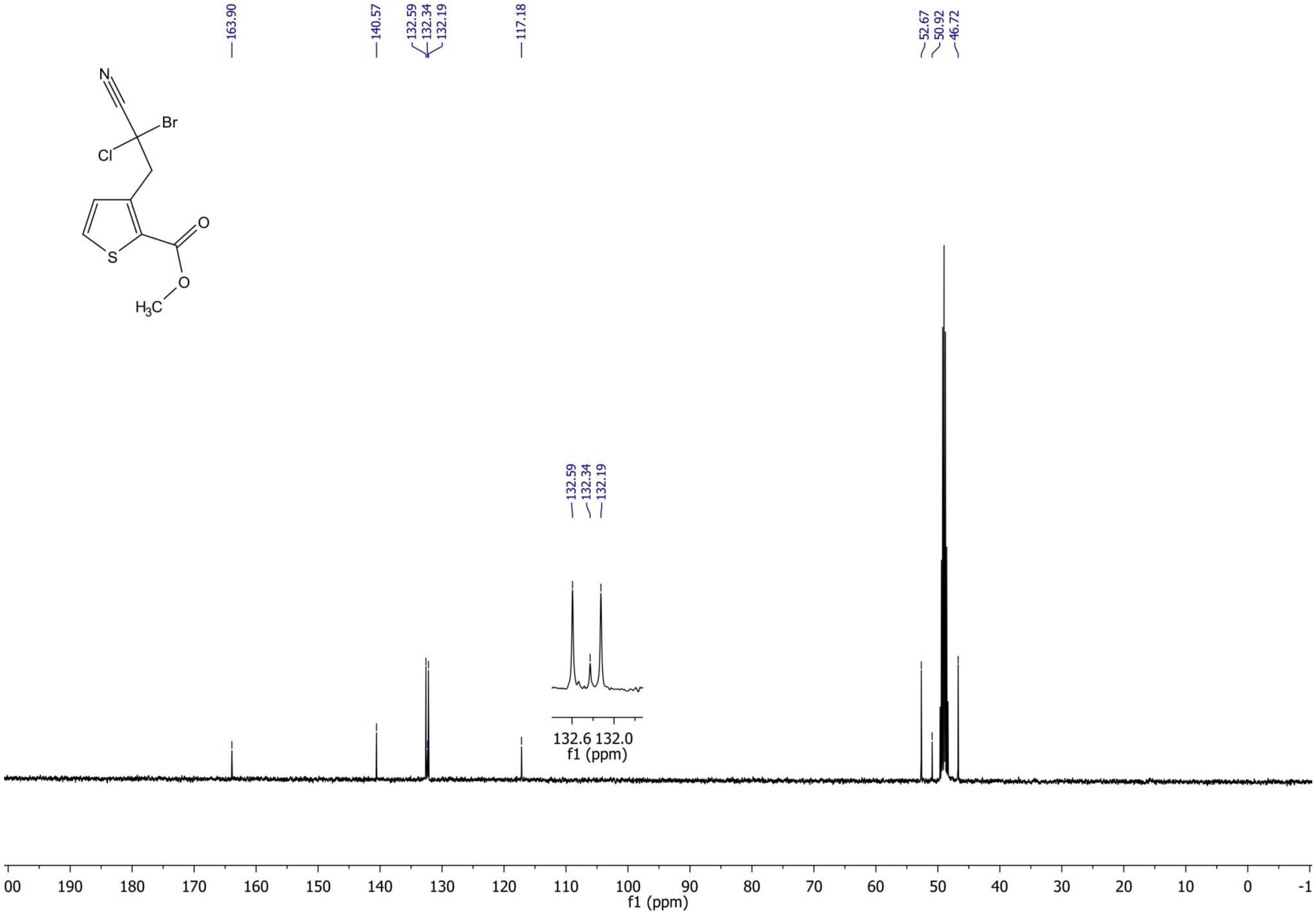

Methyl 3-(2-bromo-2-chloro-3-methoxy-3-oxopropyl)thiophene-2-carboxylate 8a.

^{1}H NMR (400 MHz, [D$_6$]DMSO)

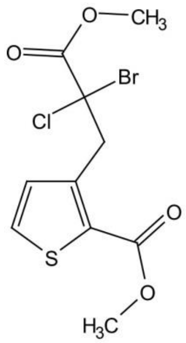

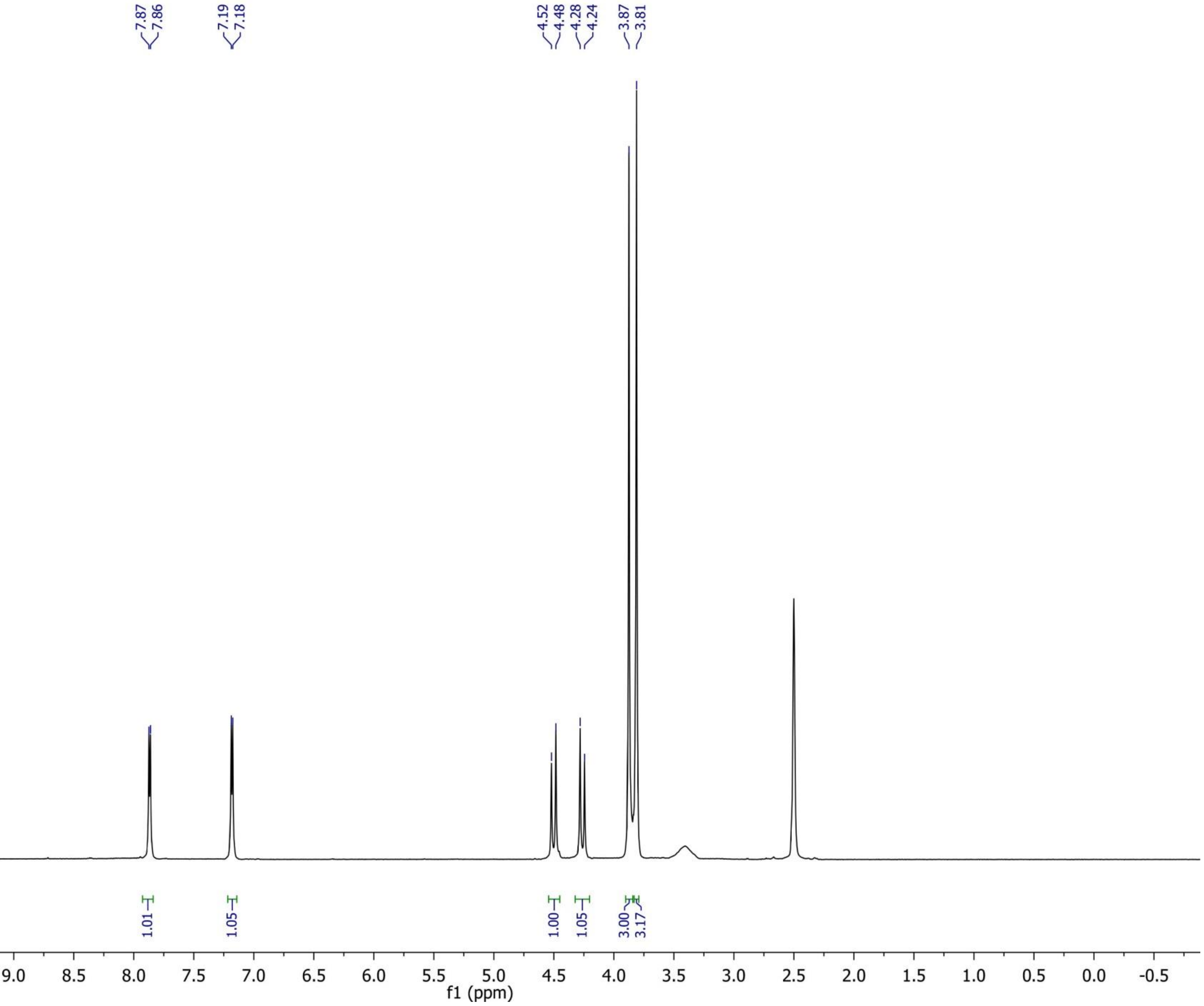

Methyl 3-(2-bromo-2-chloro-3-methoxy-3-oxopropyl)thiophene-2-carboxylate 8a.

^{13}C NMR (126 MHz, [D$_6$]DMSO)

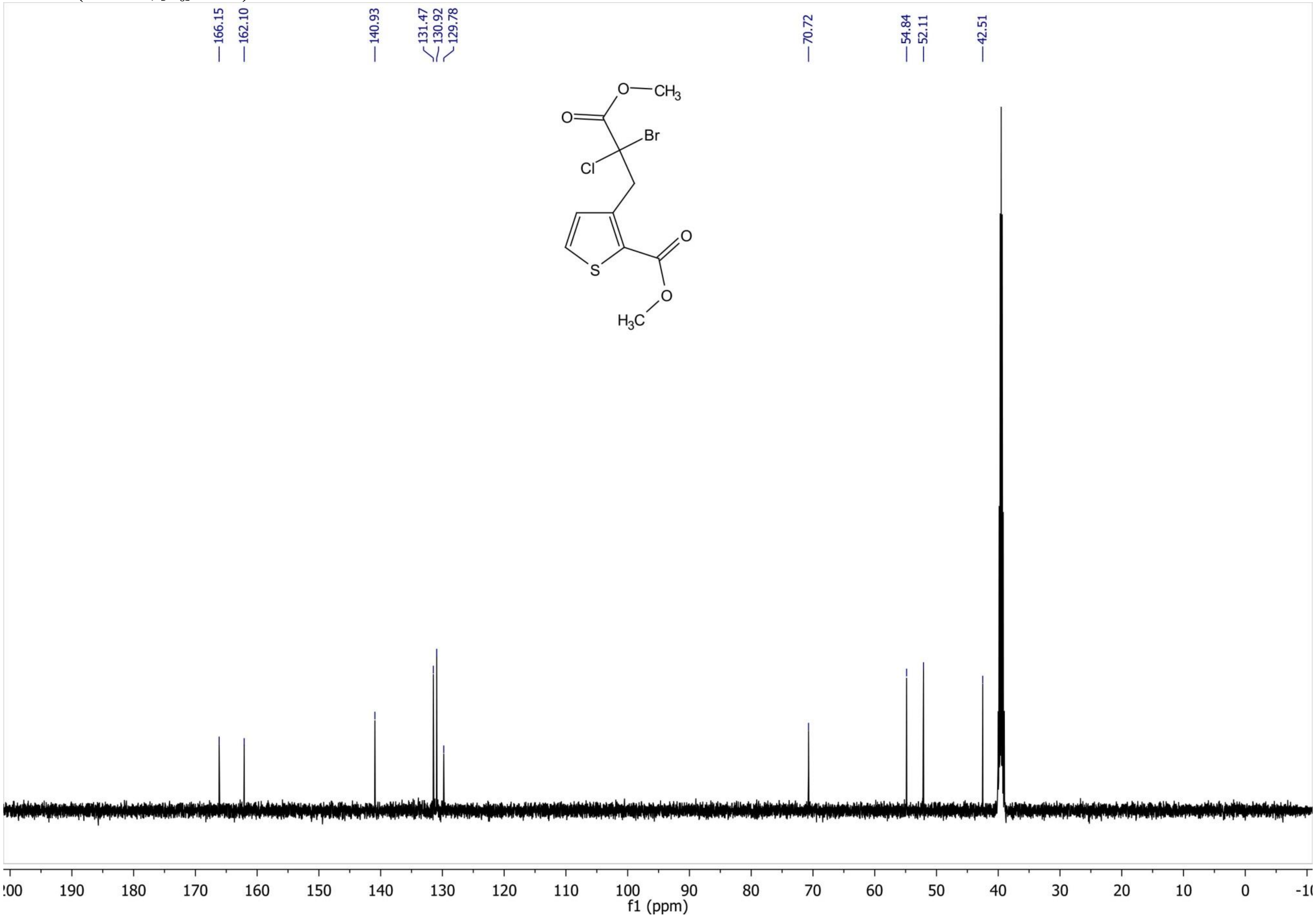

Methyl (*E*)-3-styrylthiophene-2-carboxylate 9.

^{1}H NMR (400 MHz, [D$_6$]DMSO)

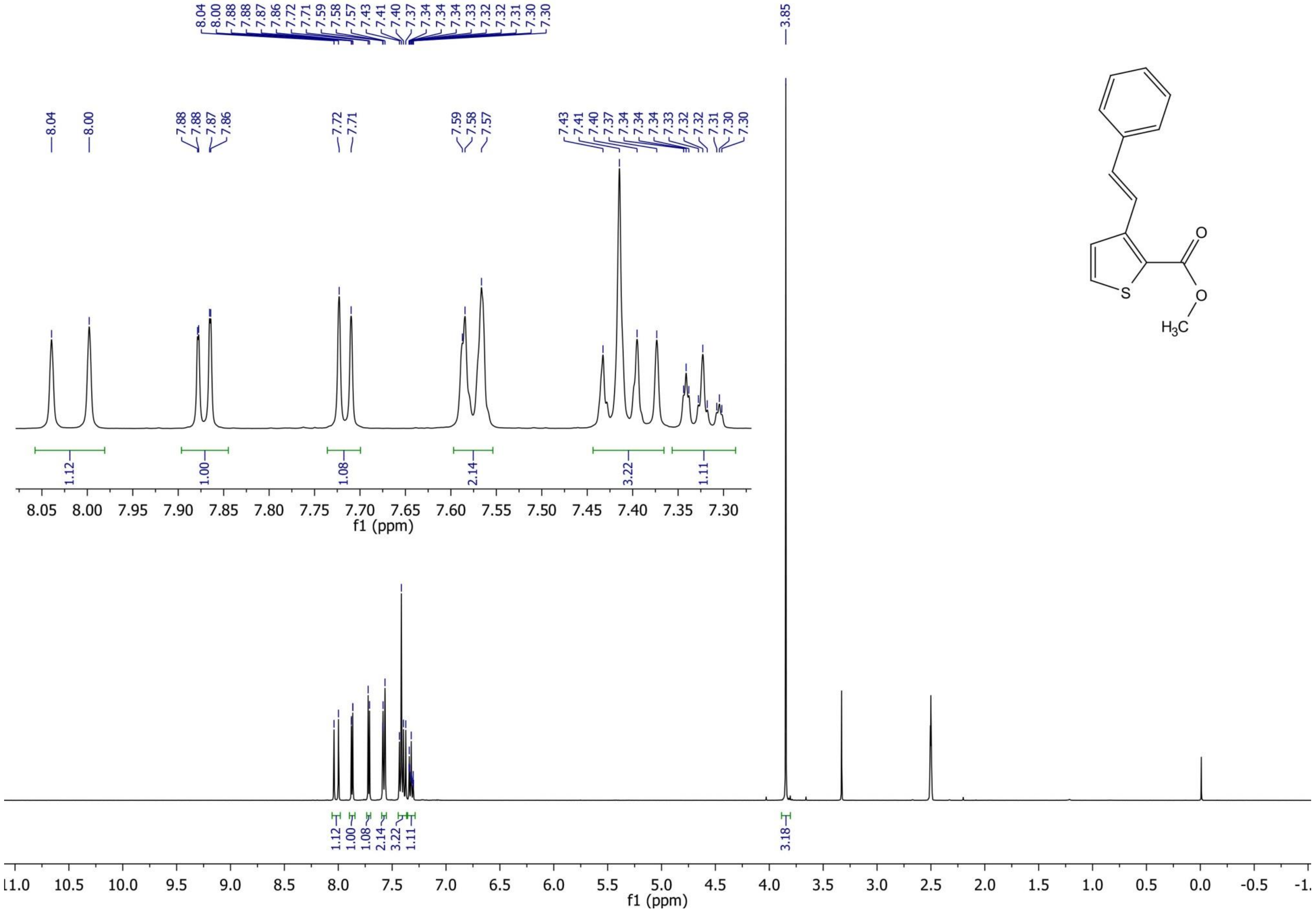

Methyl (*E*)-3-styrylthiophene-2-carboxylate 9.

^{13}C NMR (101 MHz, [D$_6$]DMSO)

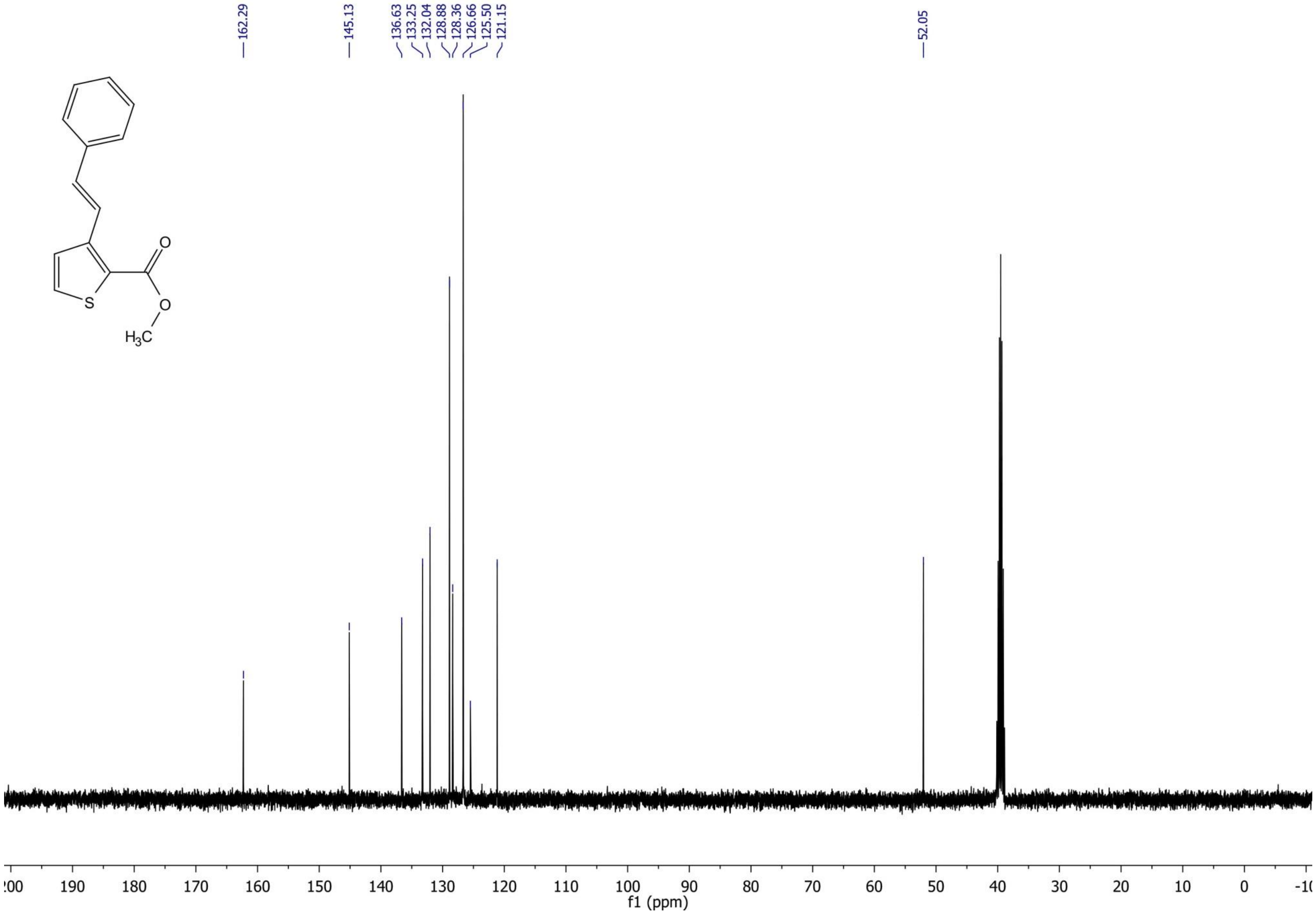

Methyl 3-(2-chloro-3-oxobutyl)thiophene-2-carboxylate 4b.

^{1}H NMR (400 MHz, [D$_6$]DMSO)

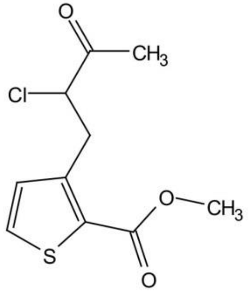

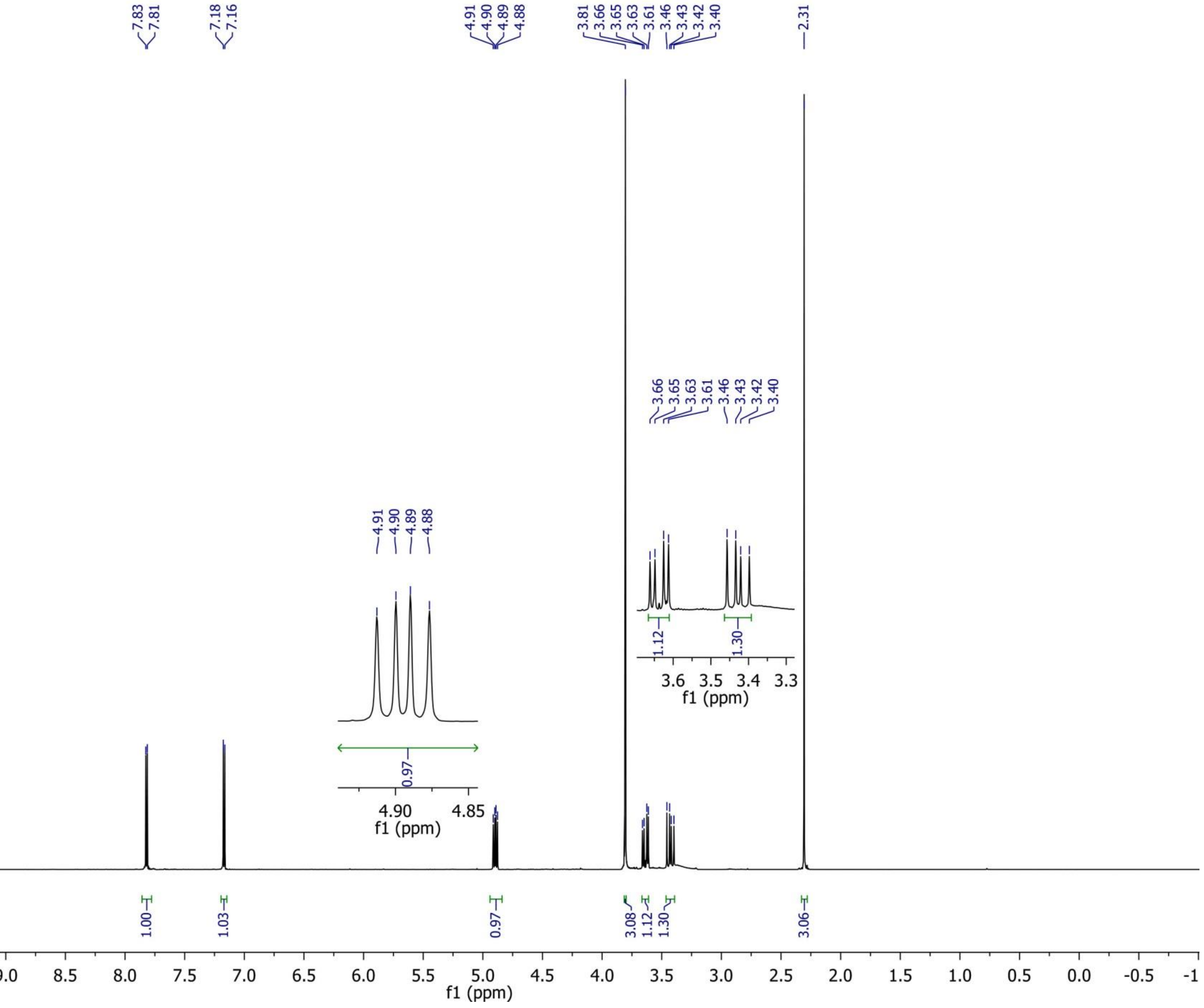

Methyl 3-(2-chloro-3-oxobutyl)thiophene-2-carboxylate 4b.

^{13}C NMR (101 MHz, [D$_6$]DMSO)

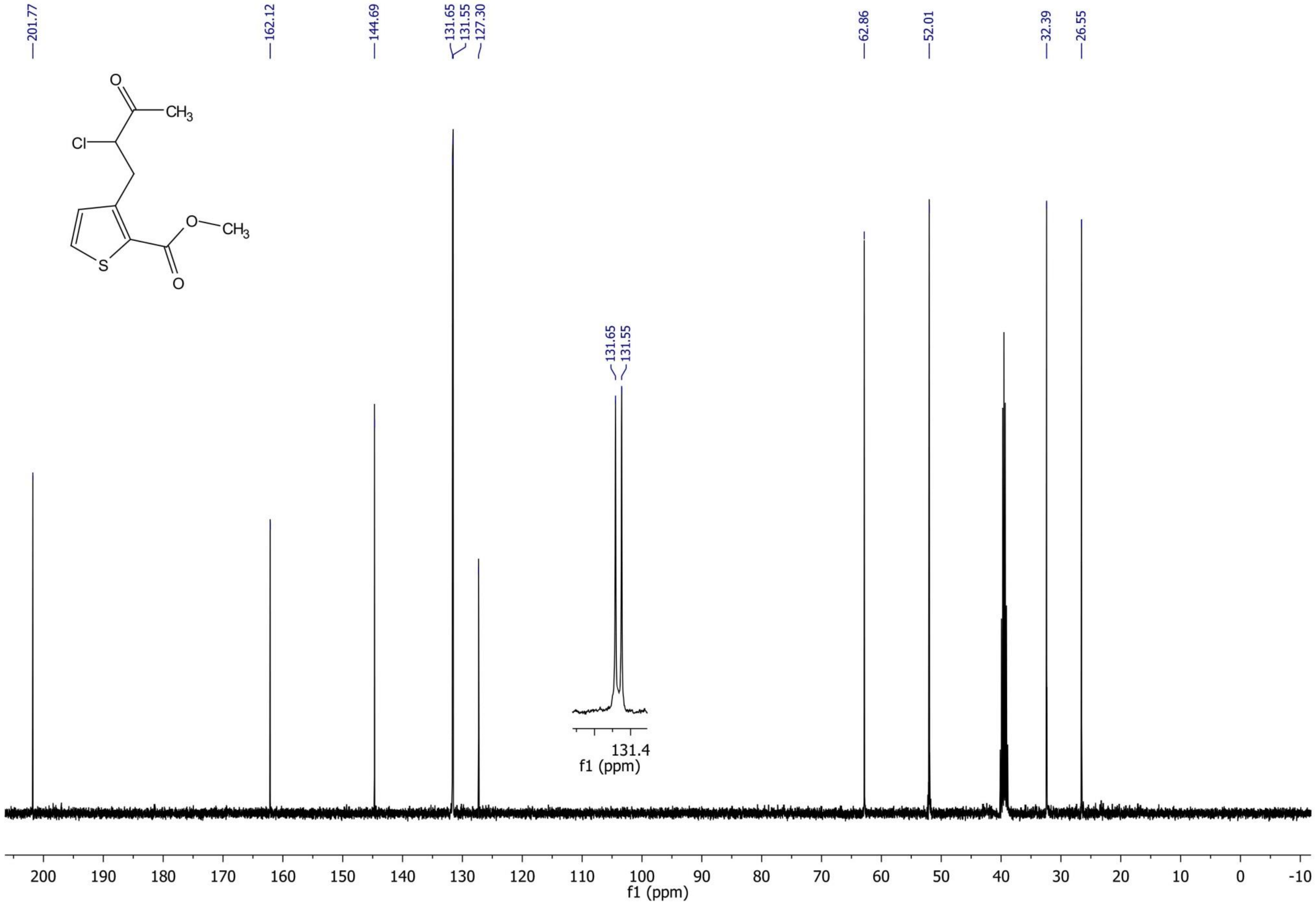

Methyl 3-(2-chloro-3-methoxy-3-oxopropyl)thiophene-2-carboxylate 5b.

^{1}H NMR (400 MHz, [D$_6$]DMSO)

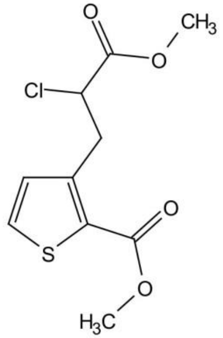

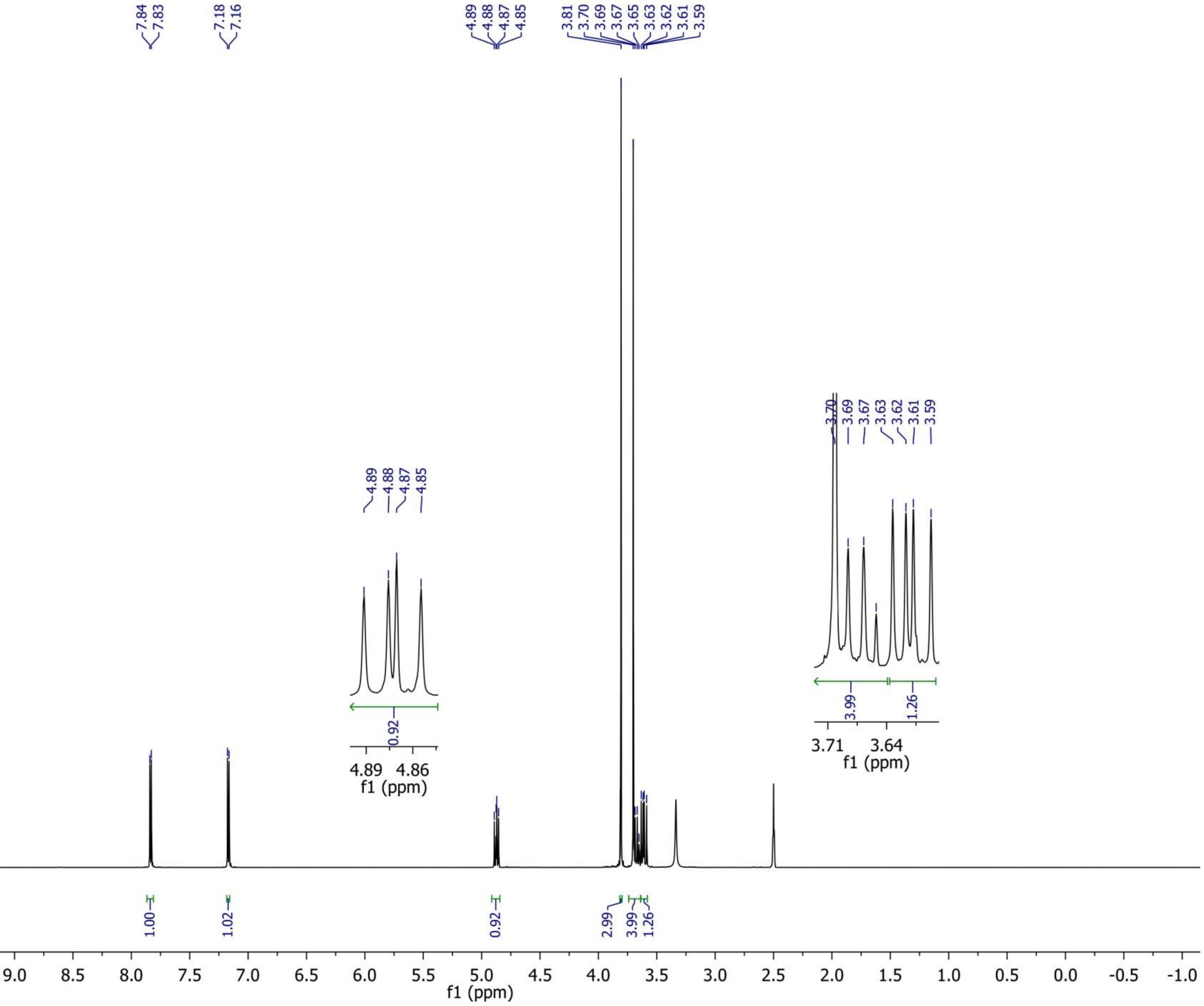

Methyl 3-(2-chloro-3-methoxy-3-oxopropyl)thiophene-2-carboxylate 5b.

^{13}C NMR (101 MHz, [D$_6$]DMSO)

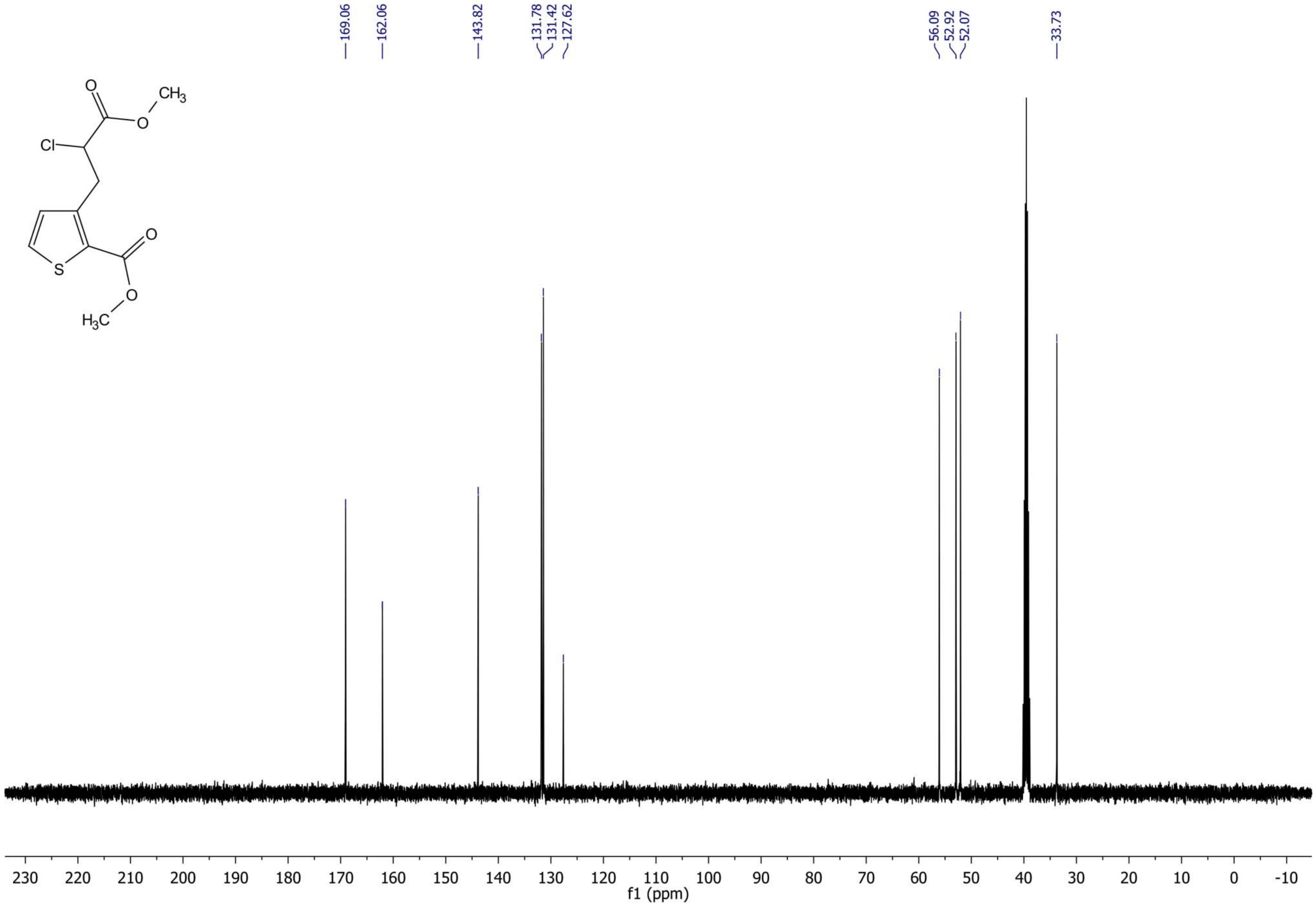

Methyl 3-(2,2-dichloro-3-methoxy-3-oxopropyl)thiophene-2-carboxylate 8b.

^{1}H NMR (400 MHz, [D_6]DMSO)

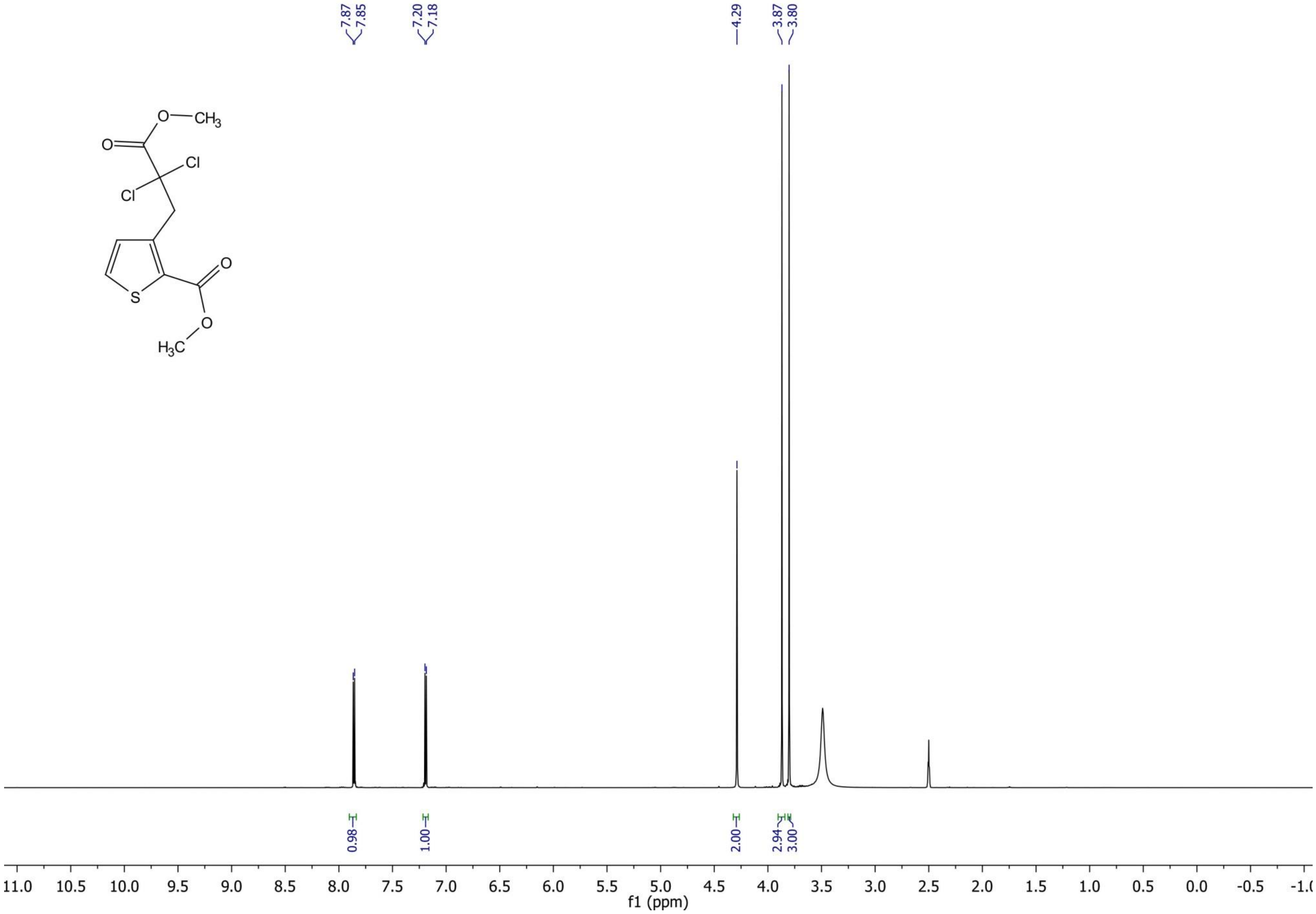

Methyl 3-(2,2-dichloro-3-methoxy-3-oxopropyl)thiophene-2-carboxylate 8b.

^{13}C NMR (101 MHz, [D$_6$]DMSO)

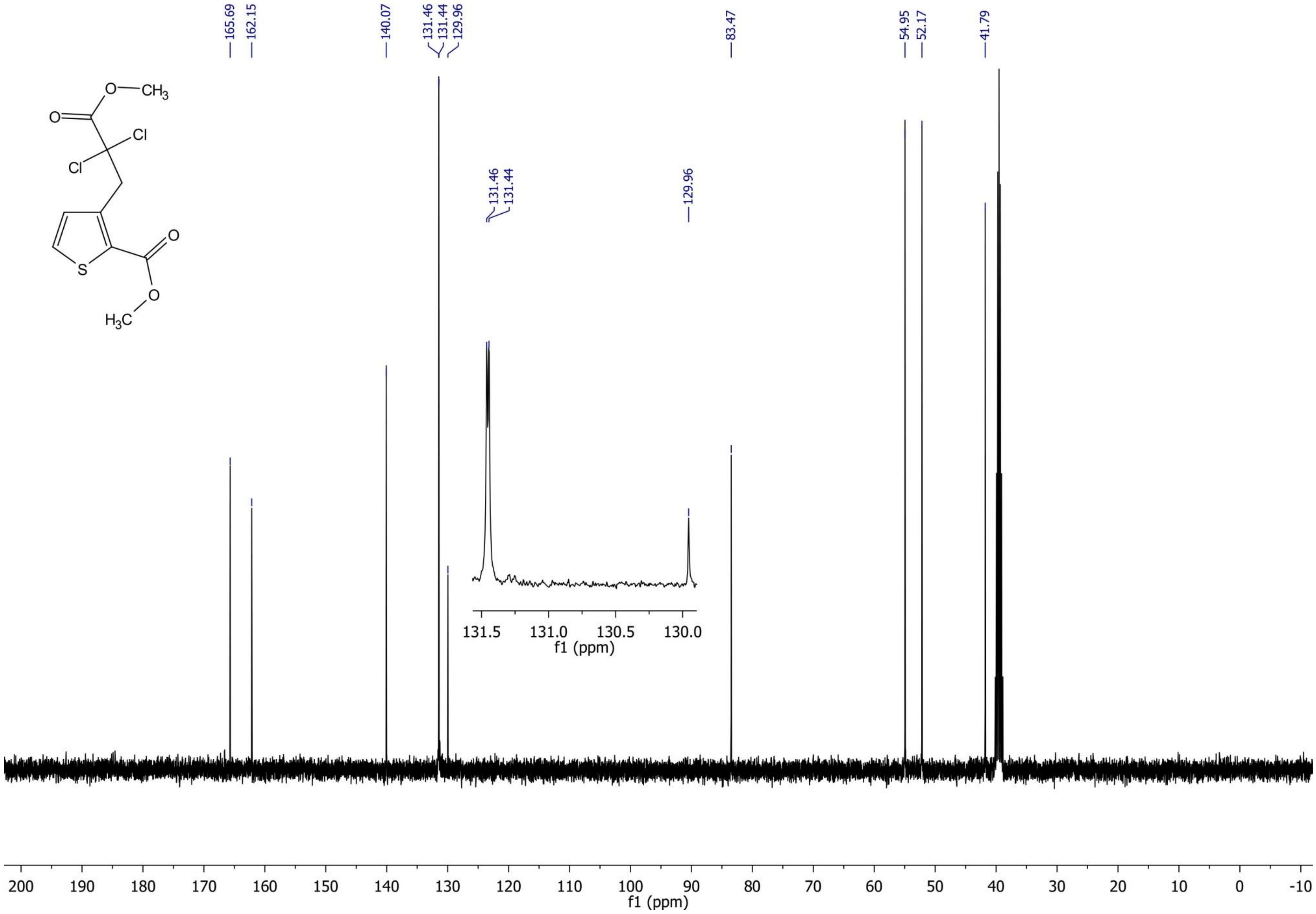

Methyl 3-(2-chloro-3-oxopropyl)thiophene-2-carboxylate 11.

^{1}H NMR (400 MHz, $CDCl_3$)

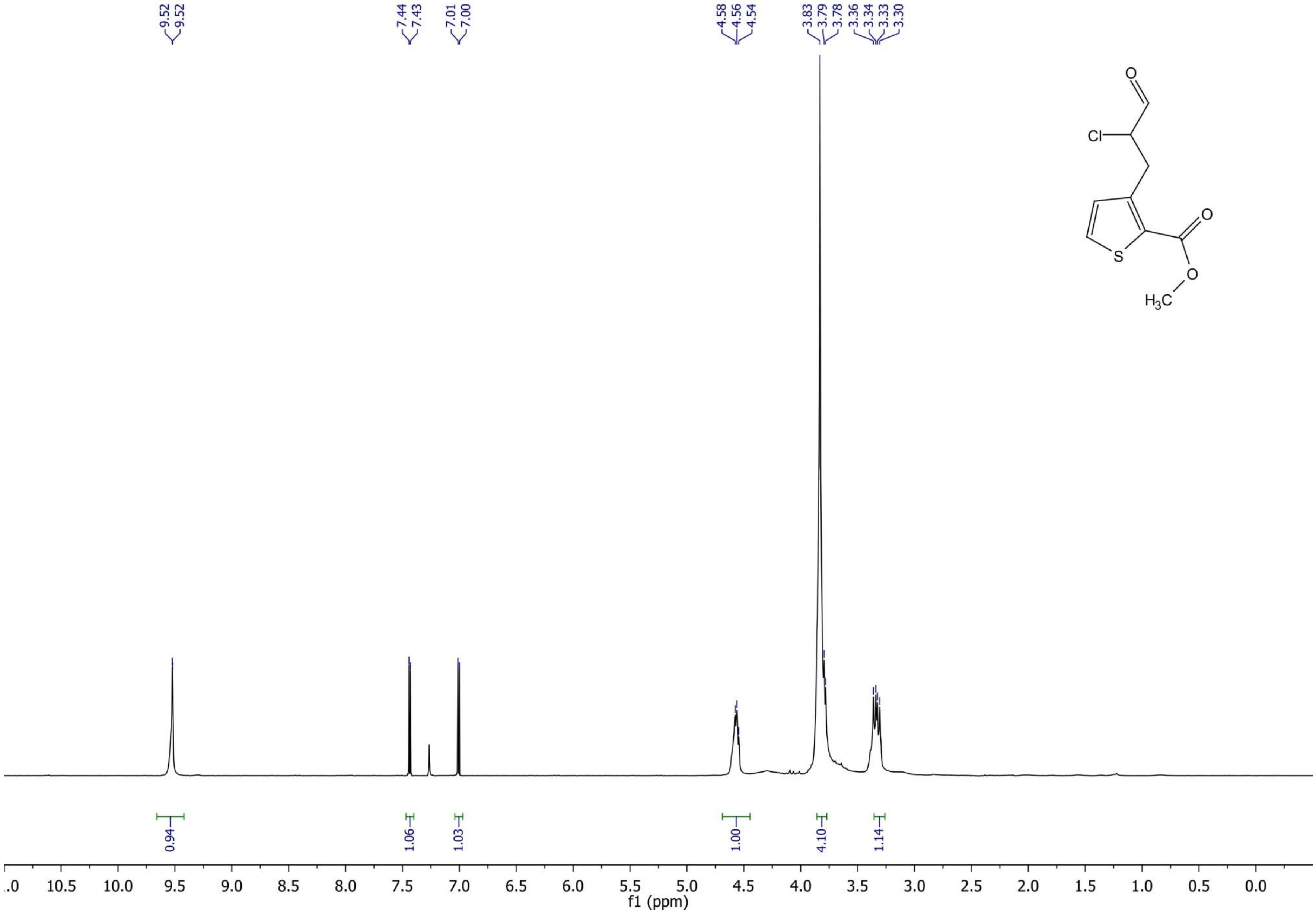

Methyl 3-(2-chloro-3-oxopropyl)thiophene-2-carboxylate 11.

^{13}C NMR (101 MHz, $CDCl_3$)

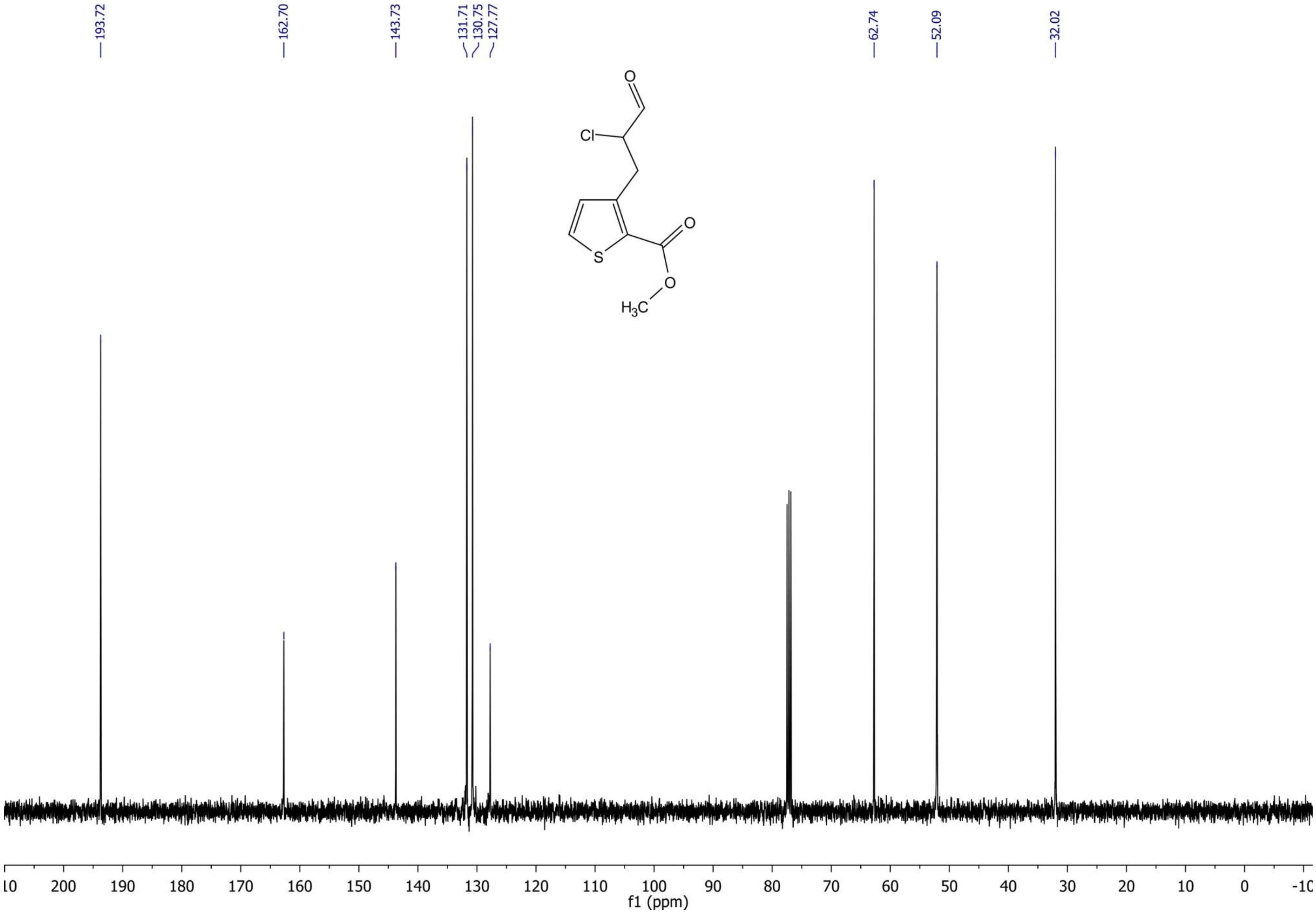

Methyl 3-(2-chloro-3-methoxy-2-methyl-3-oxopropyl)thiophene-2-carboxylate 12.

^{1}H NMR (400 MHz, $CDCl_3$)

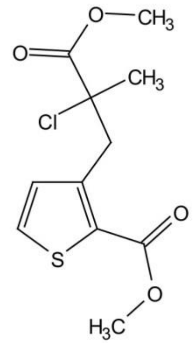

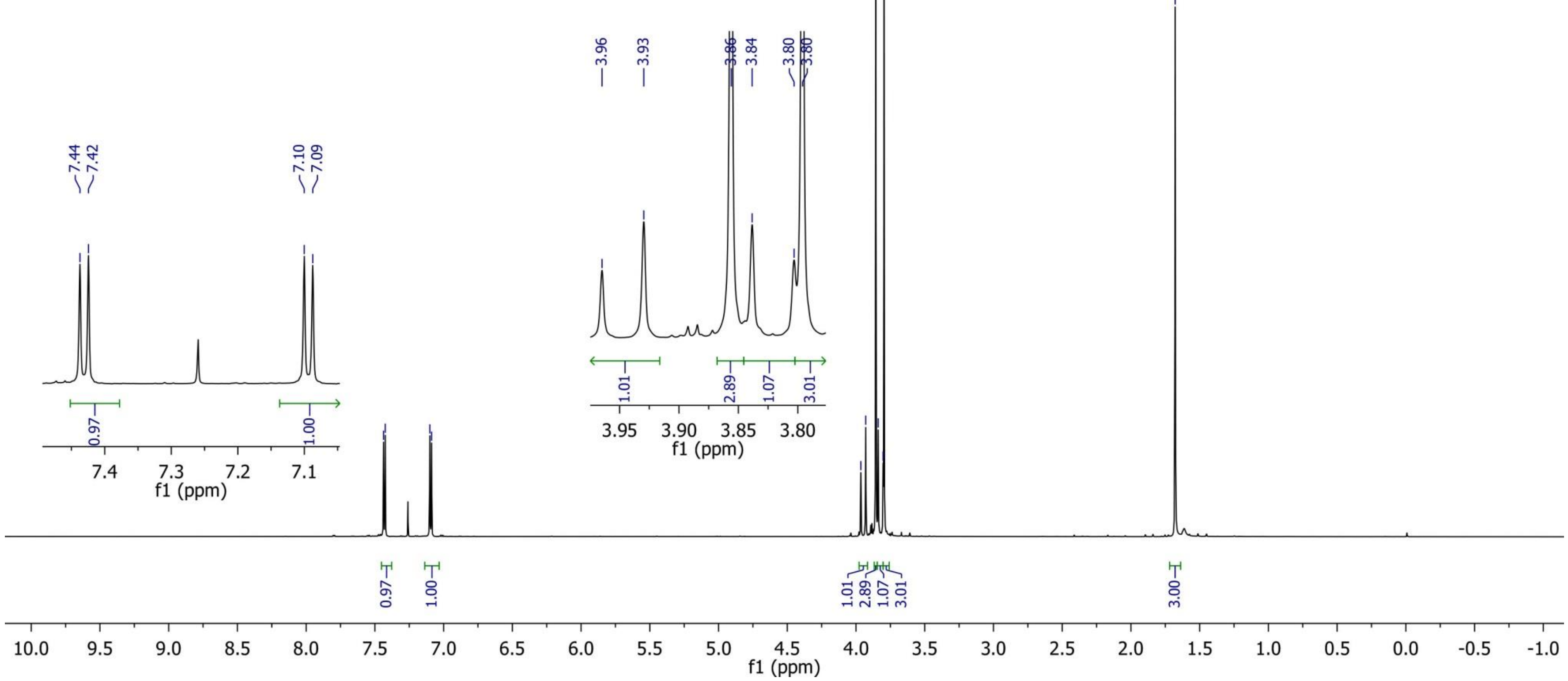

Methyl 3-(2-chloro-3-methoxy-2-methyl-3-oxopropyl)thiophene-2-carboxylate 12.

^{13}C NMR (101 MHz, $CDCl_3$)

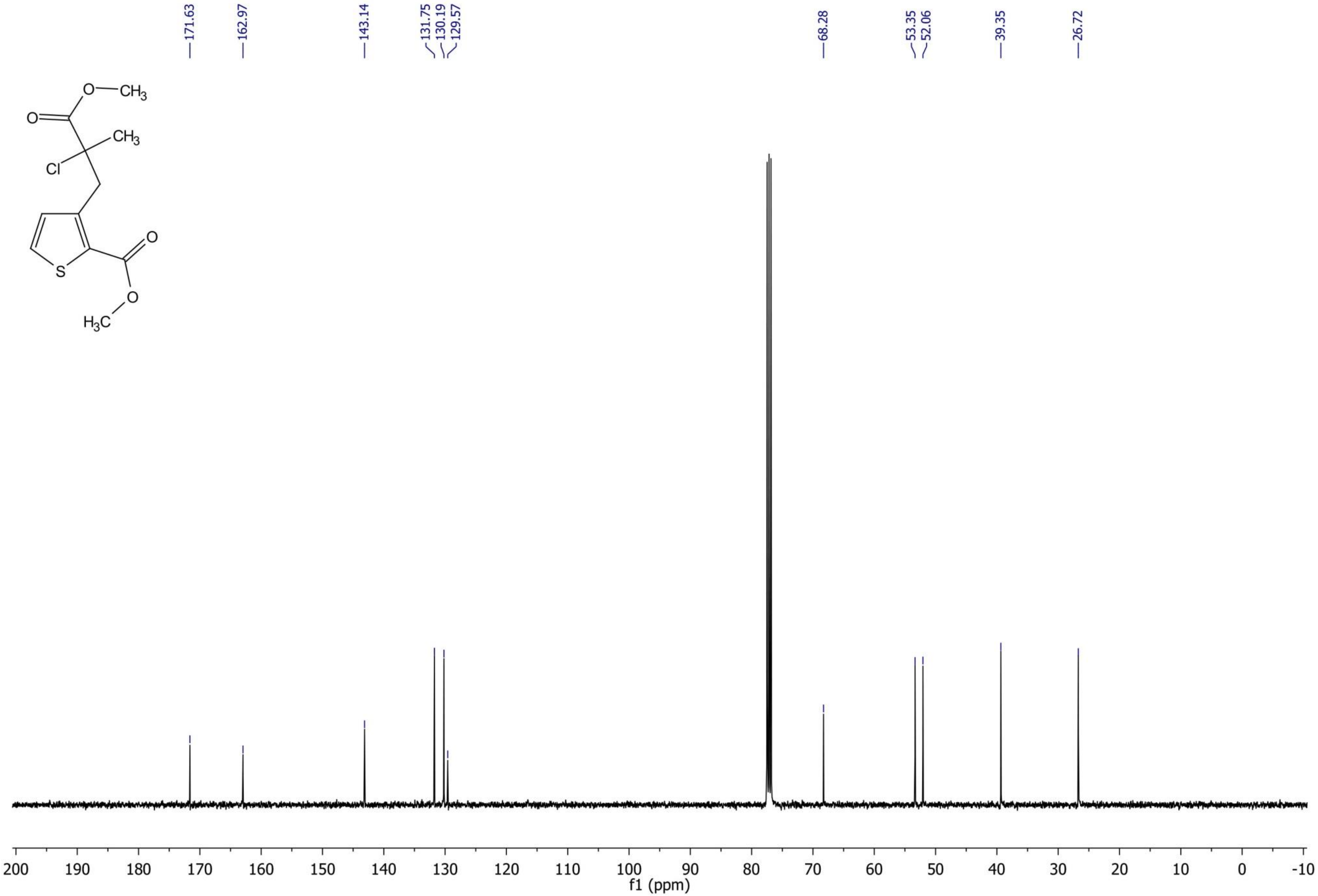

(E)-3-(2-Carboxyvinyl)thiophene-2-carboxylic acid 13.

^{1}H NMR (400 MHz, [D$_6$]DMSO)

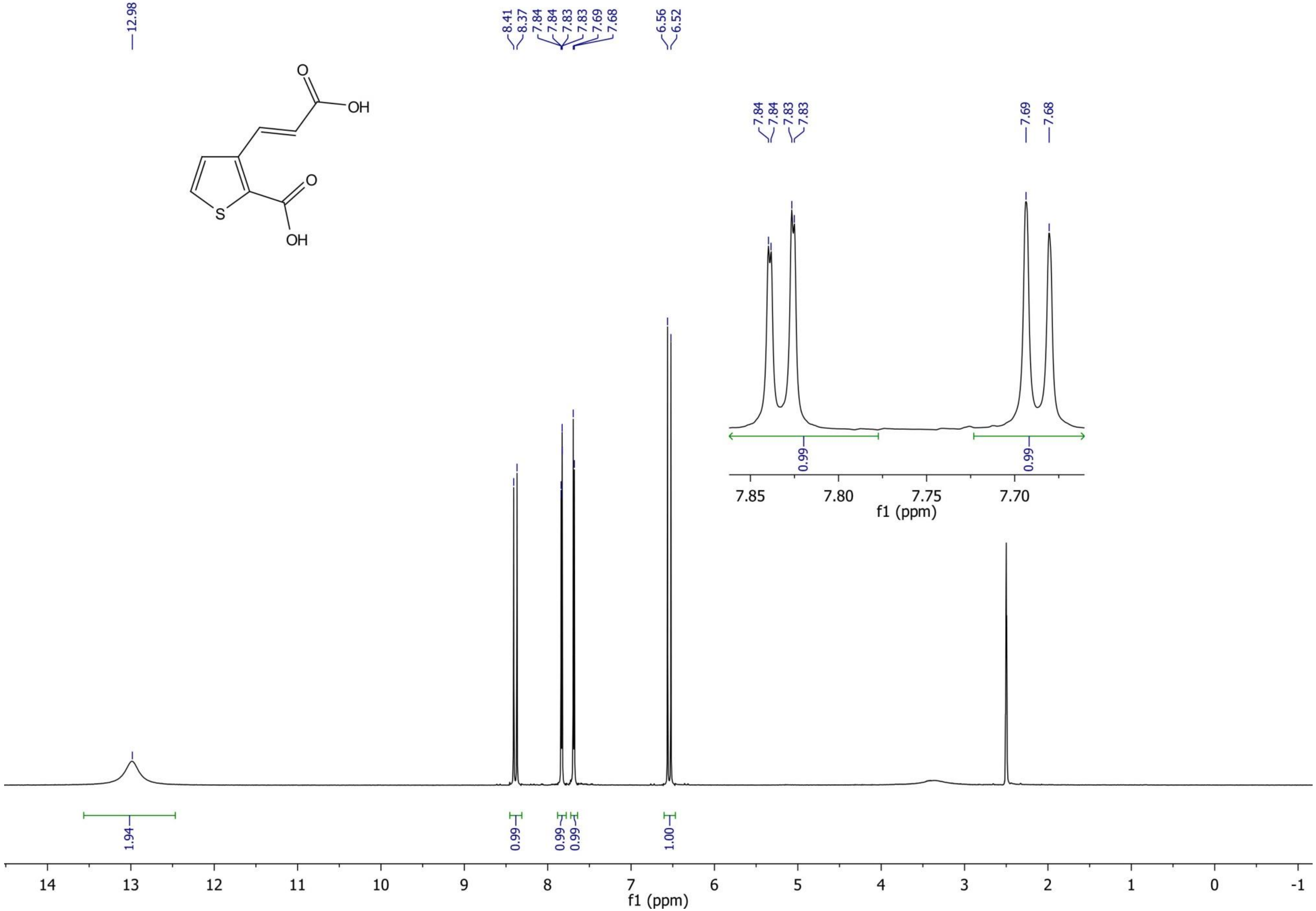

(*E*)-3-(2-Carboxyvinyl)thiophene-2-carboxylic acid 13.

^{13}C NMR (101 MHz, [D$_6$]DMSO)

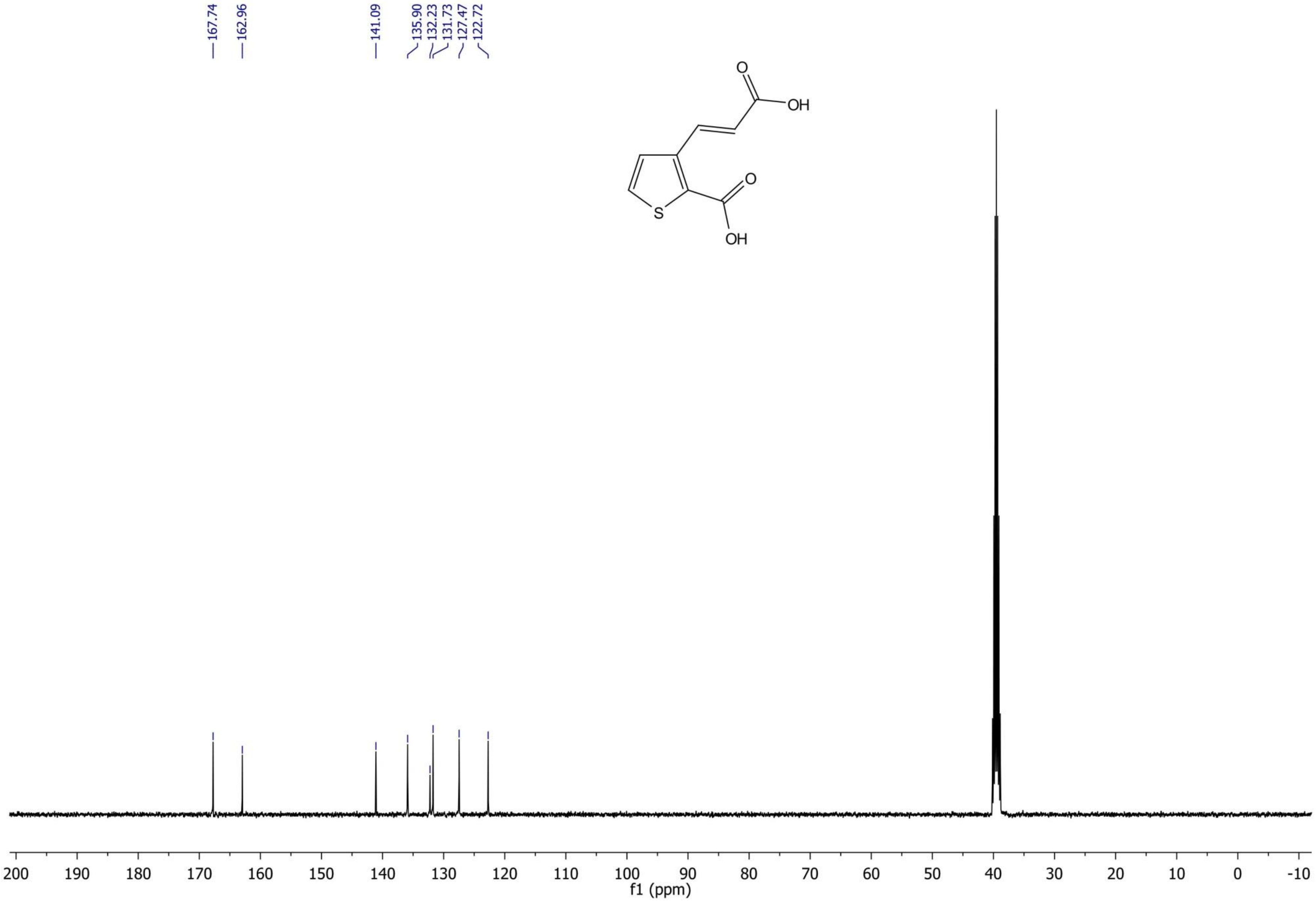

(*E*)-3-(2-Cyanovinyl)thiophene-2-carboxylic acid 14a.

^{1}H NMR (400 MHz, [D$_6$]DMSO)

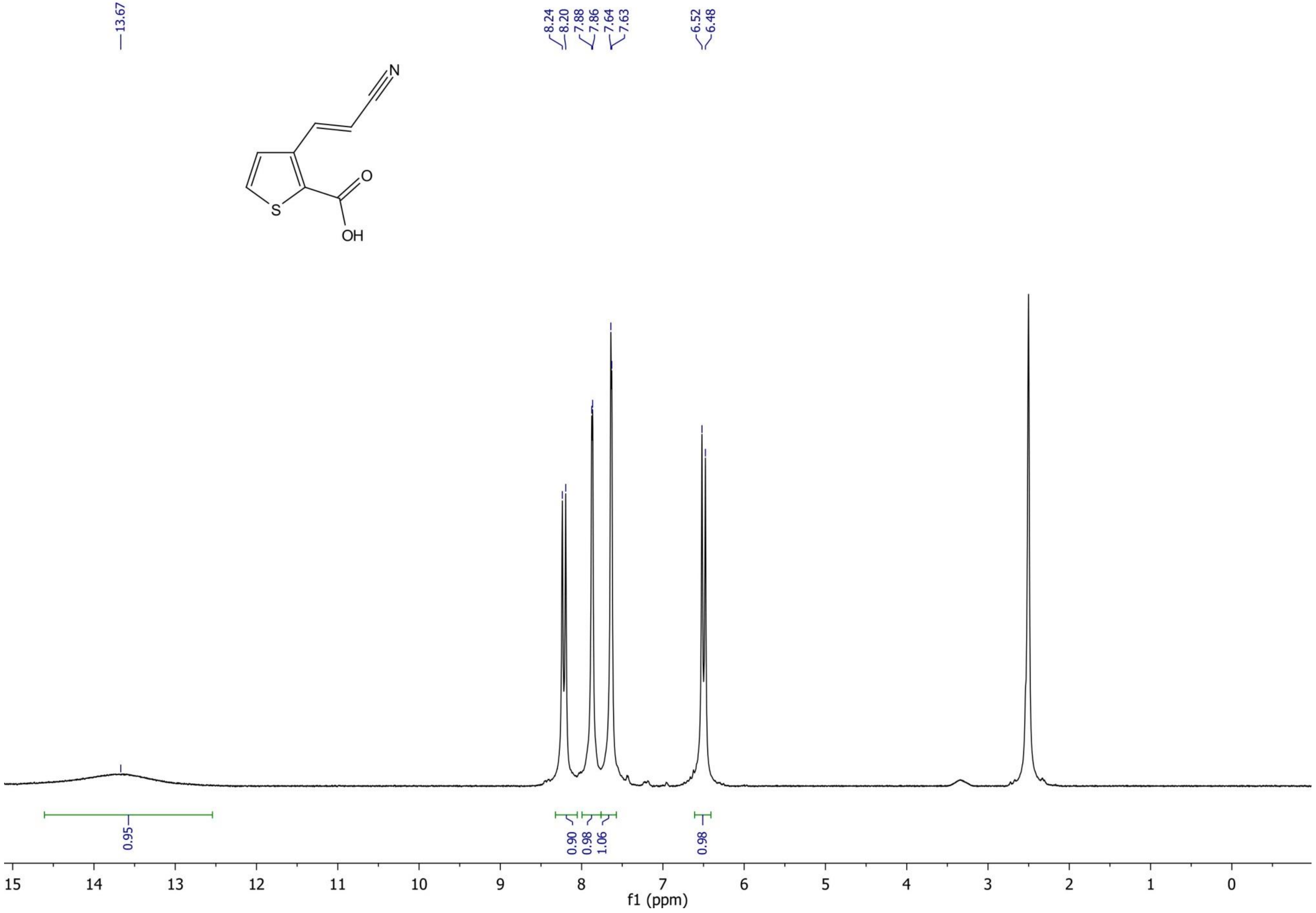

(*E*)-3-(2-Cyanovinyl)thiophene-2-carboxylic acid 14a.

^{13}C NMR (101 MHz, [D$_6$]DMSO)

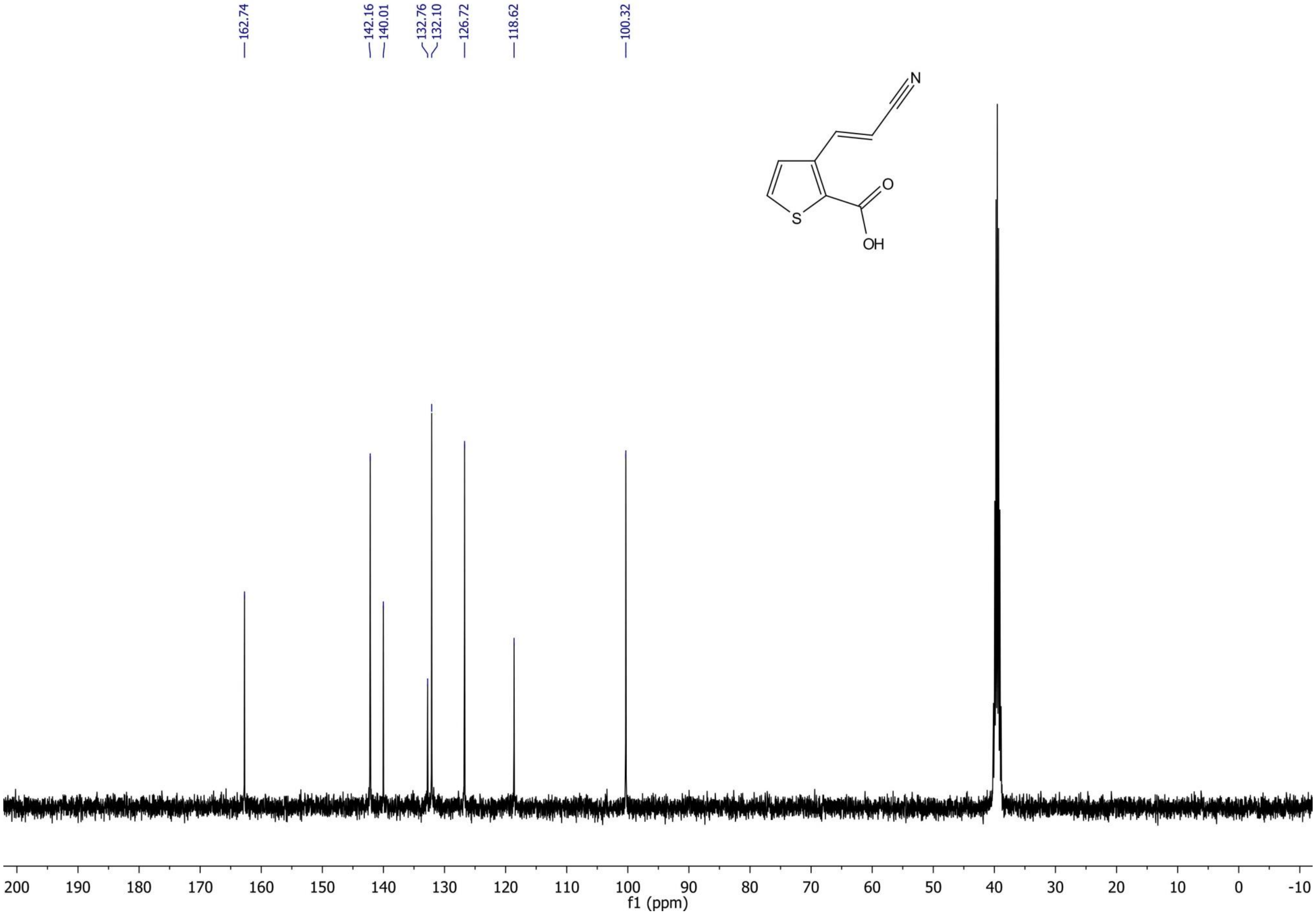

Methyl (*E*)-3-(2-cyanovinyl)thiophene-2-carboxylate 14b.

^{1}H NMR (400 MHz, [D$_6$]DMSO)

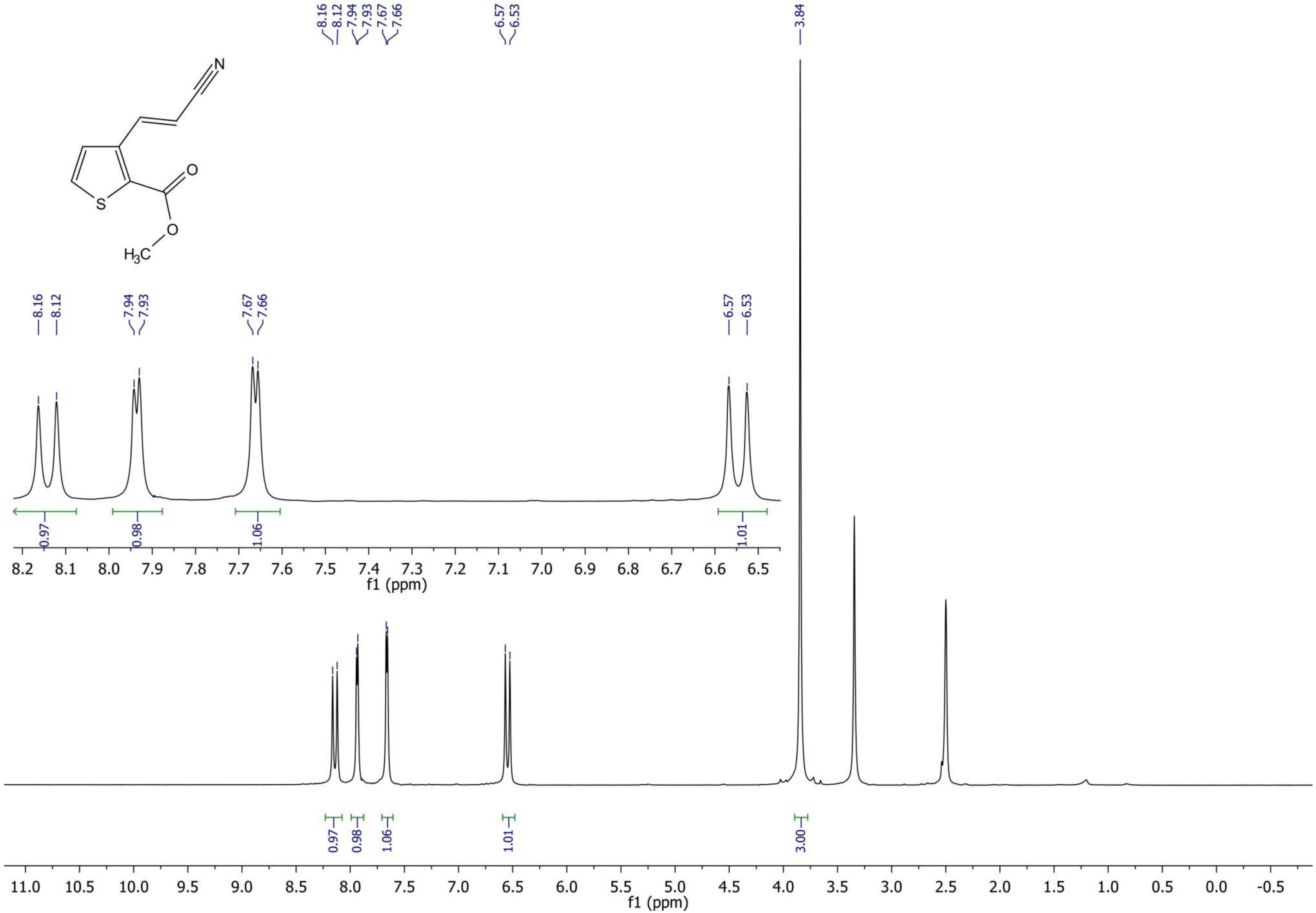

Methyl (*E*)-3-(2-cyanovinyl)thiophene-2-carboxylate 14b.

^{13}C NMR (101 MHz, [D$_6$]DMSO)

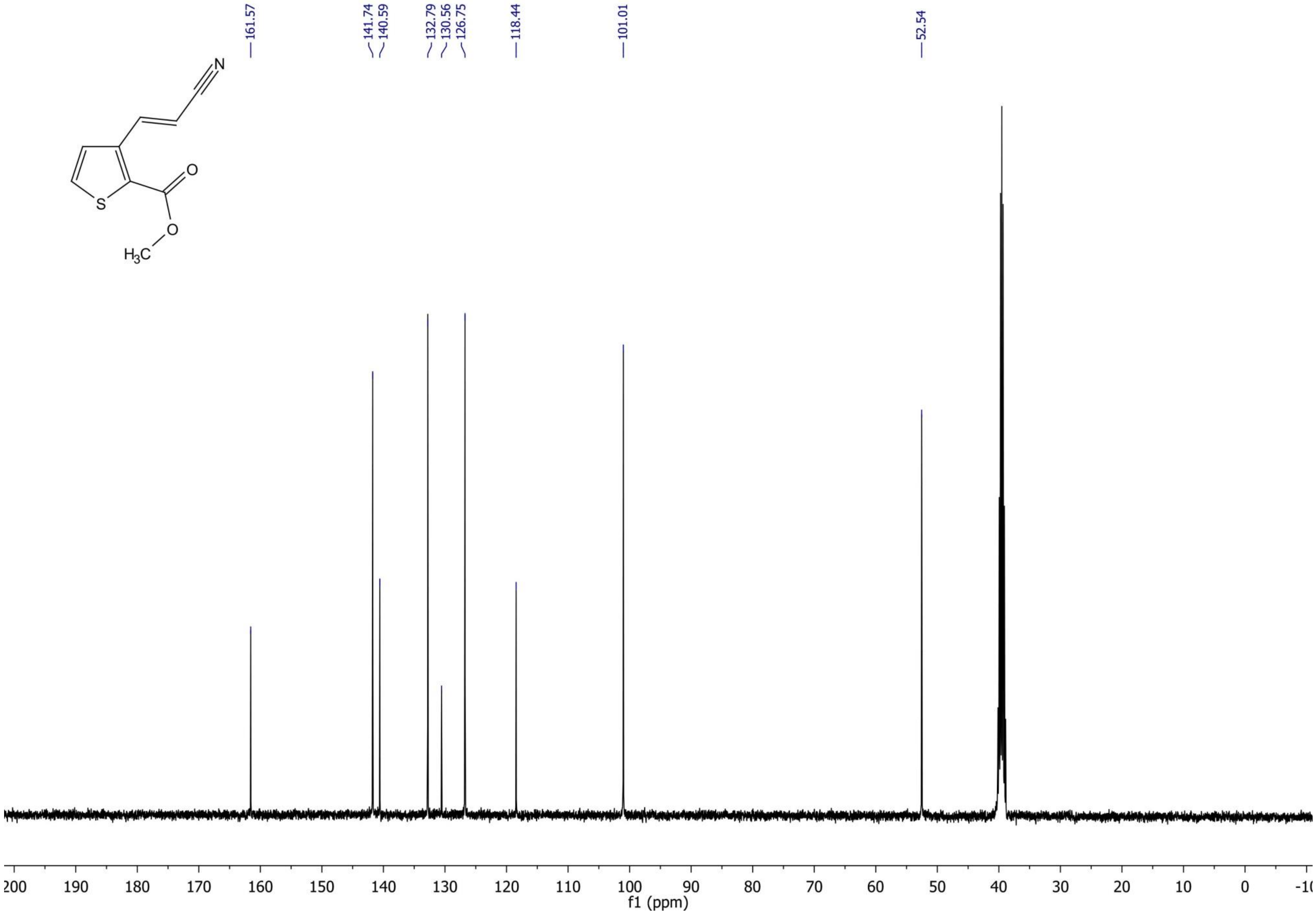

3-(2-Carboxy-2-chlorovinyl)thiophene-2-carboxylic acid 15.

^{1}H NMR (400 MHz, [D$_6$]DMSO)

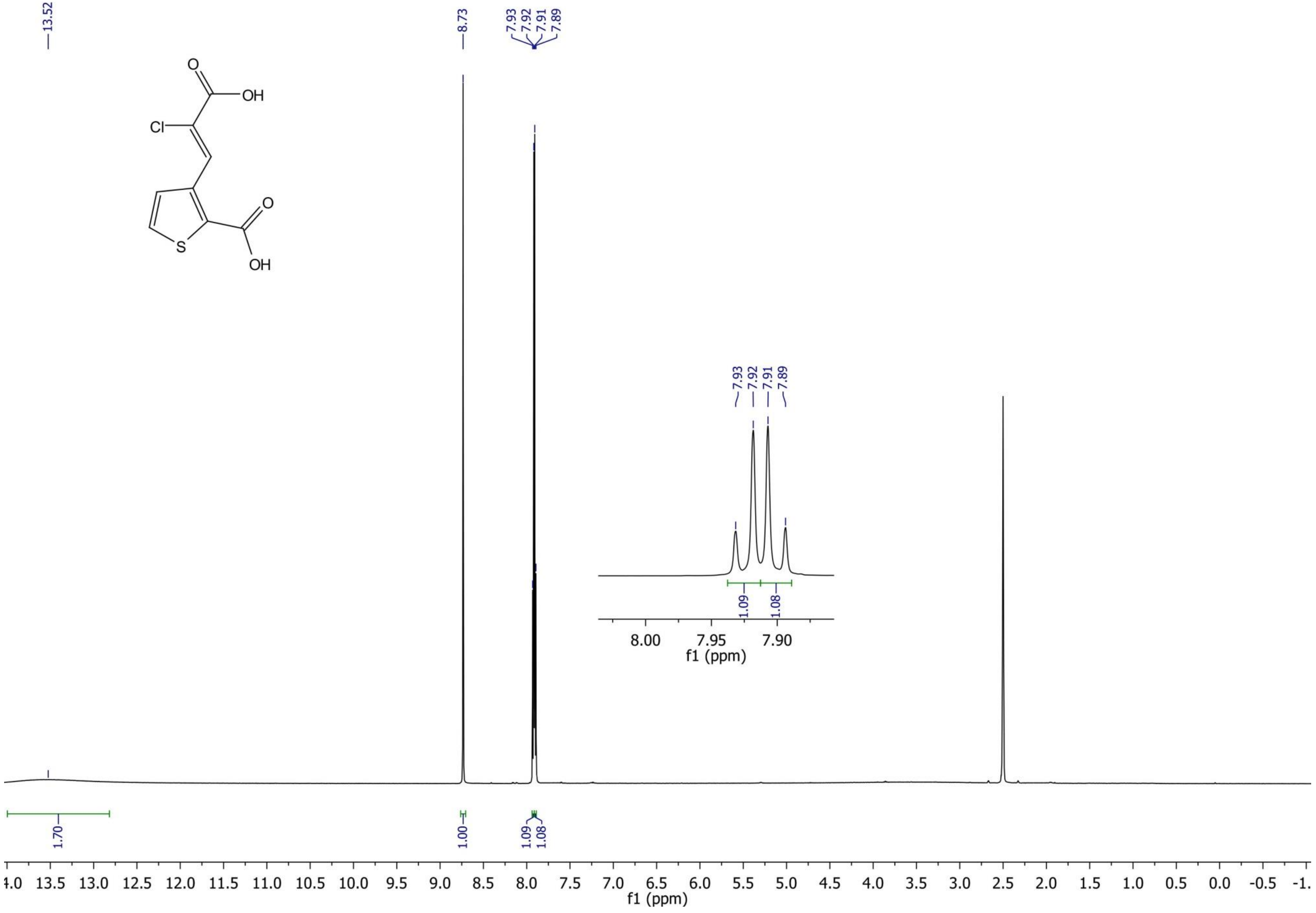

3-(2-Carboxy-2-chlorovinyl)thiophene-2-carboxylic acid 15.

^{13}C NMR (101 MHz, [D$_6$]DMSO)

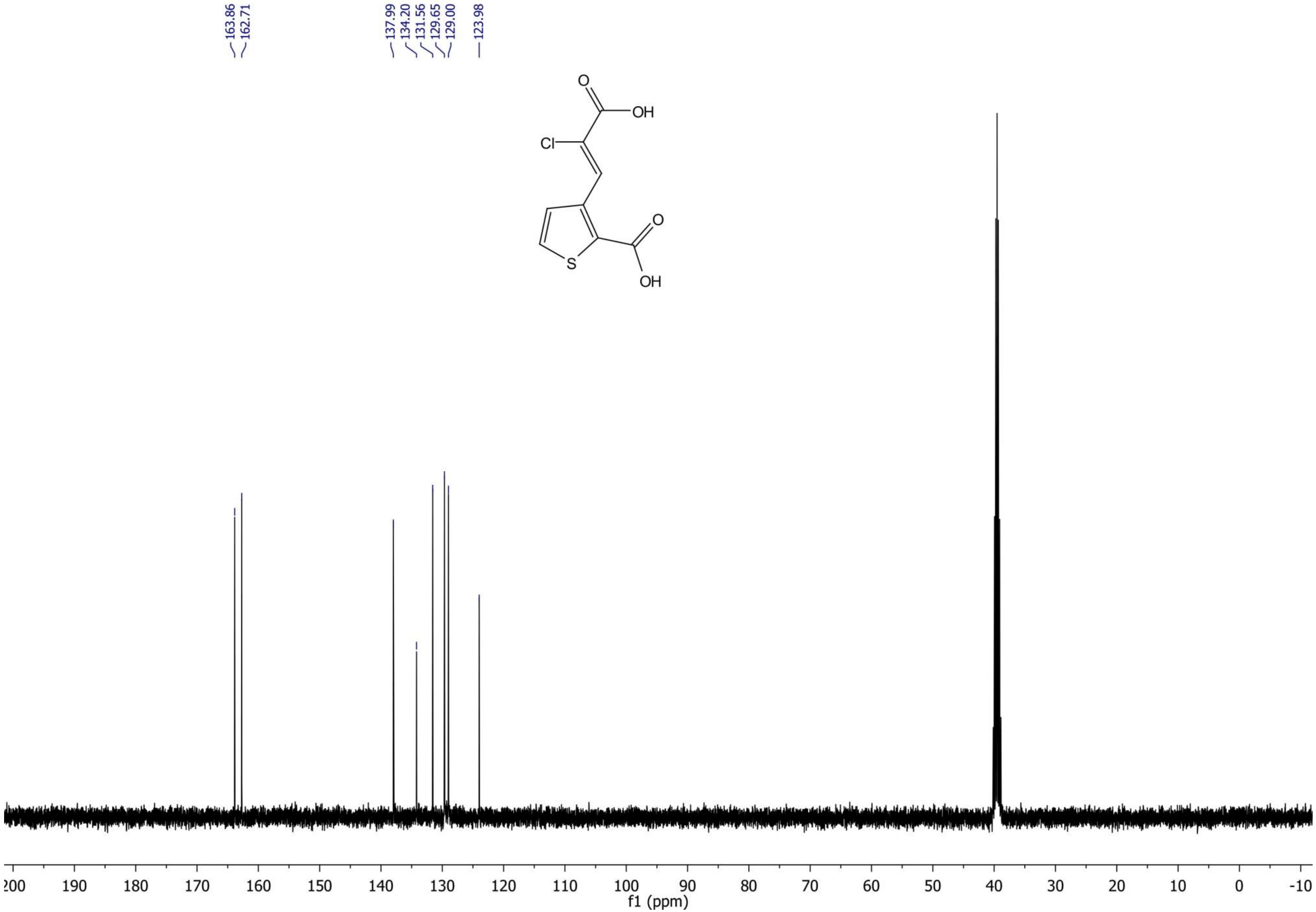

7-Oxo-4,7-dihydro-5H-thieno[2,3-*c*]pyran-5-carboxylic acid 16.

^{1}H NMR (400 MHz, [D$_6$]DMSO)

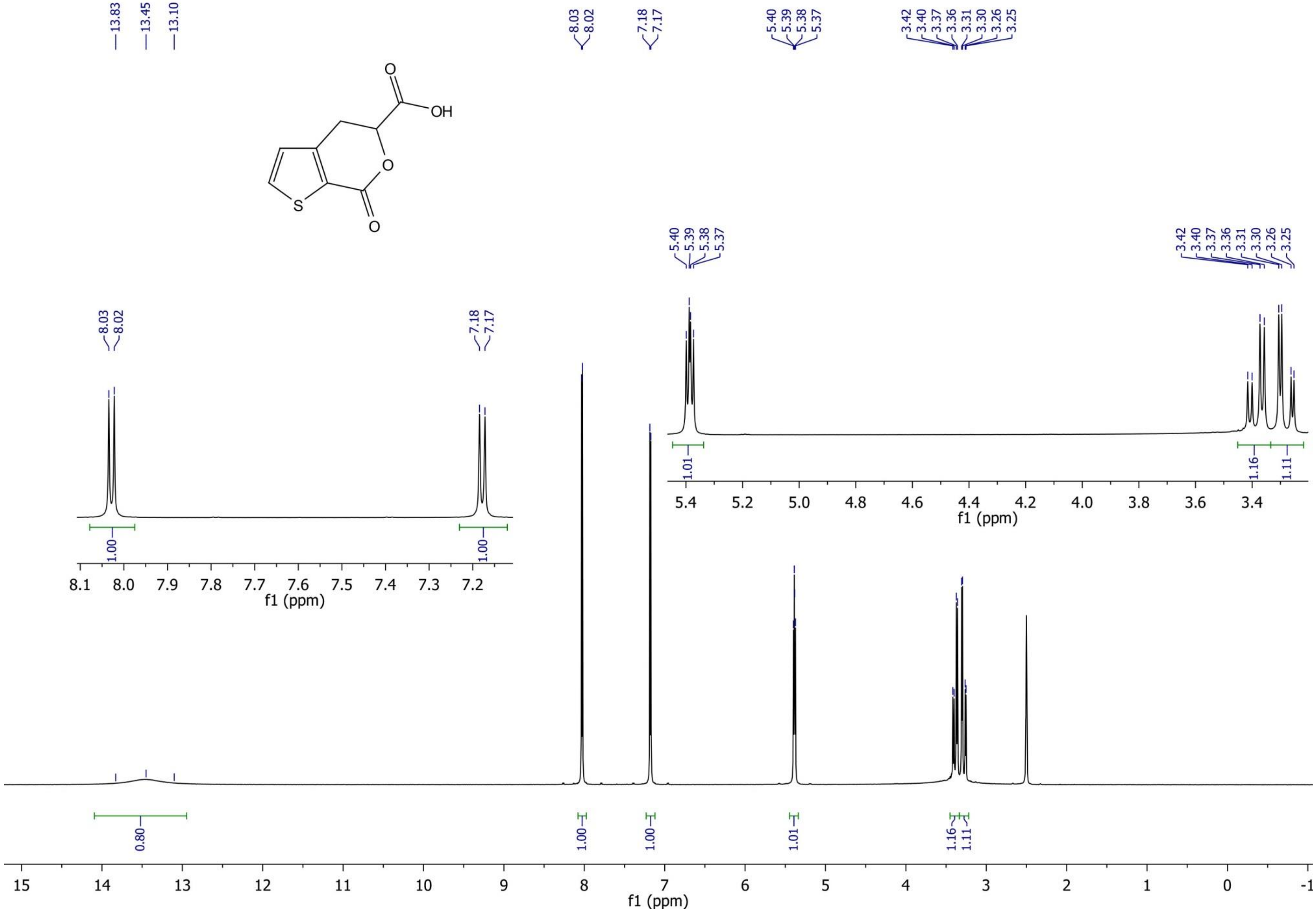

7-Oxo-4,7-dihydro-5H-thieno[2,3-*c*]pyran-5-carboxylic acid 16.

^{13}C NMR (101 MHz, [D$_6$]DMSO)

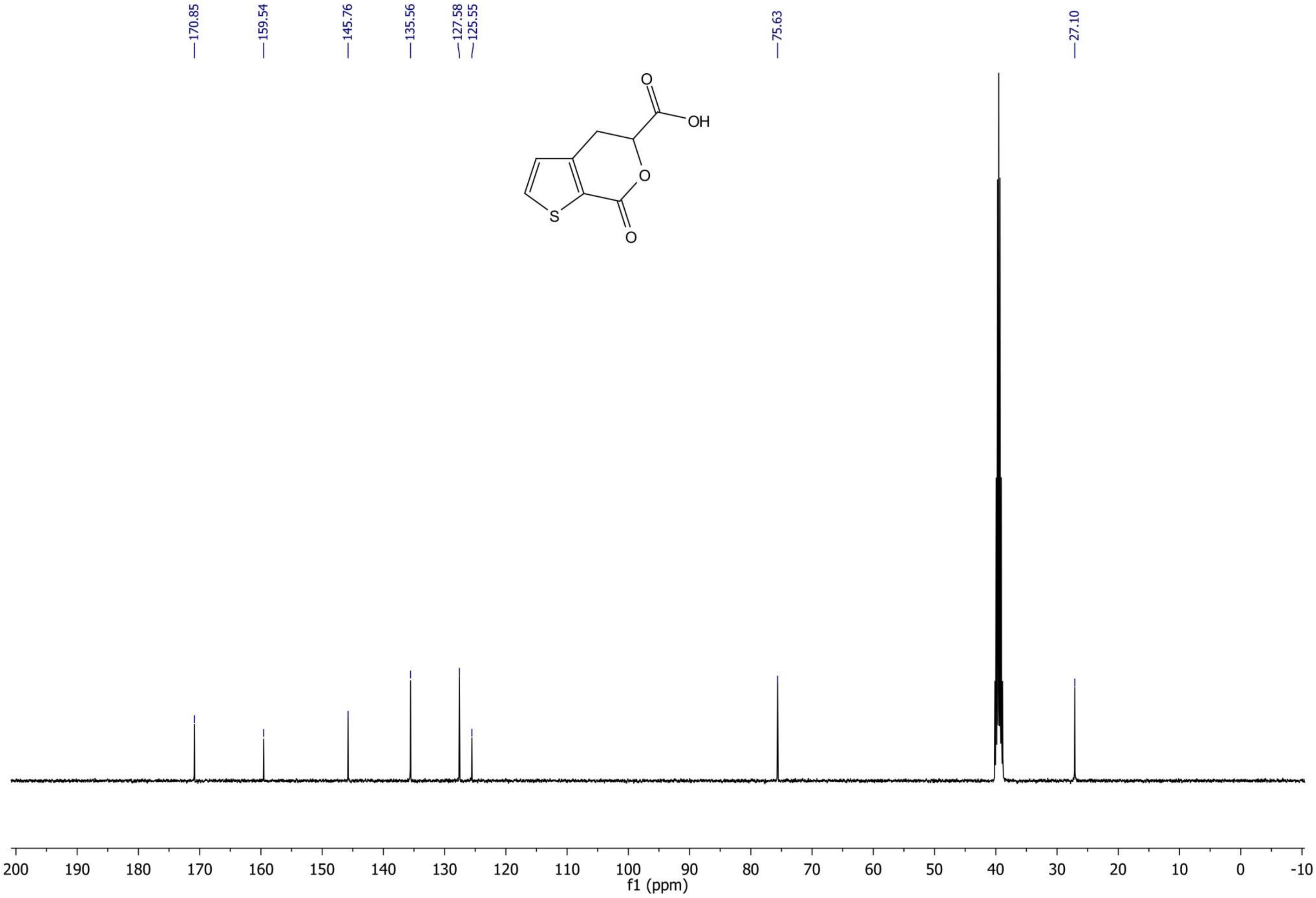

2,2-Dichloro-3-(2-(methoxycarbonyl)thiophen-3-yl)propanoic acid 17.

^{1}H NMR (400 MHz, [D_6]DMSO)

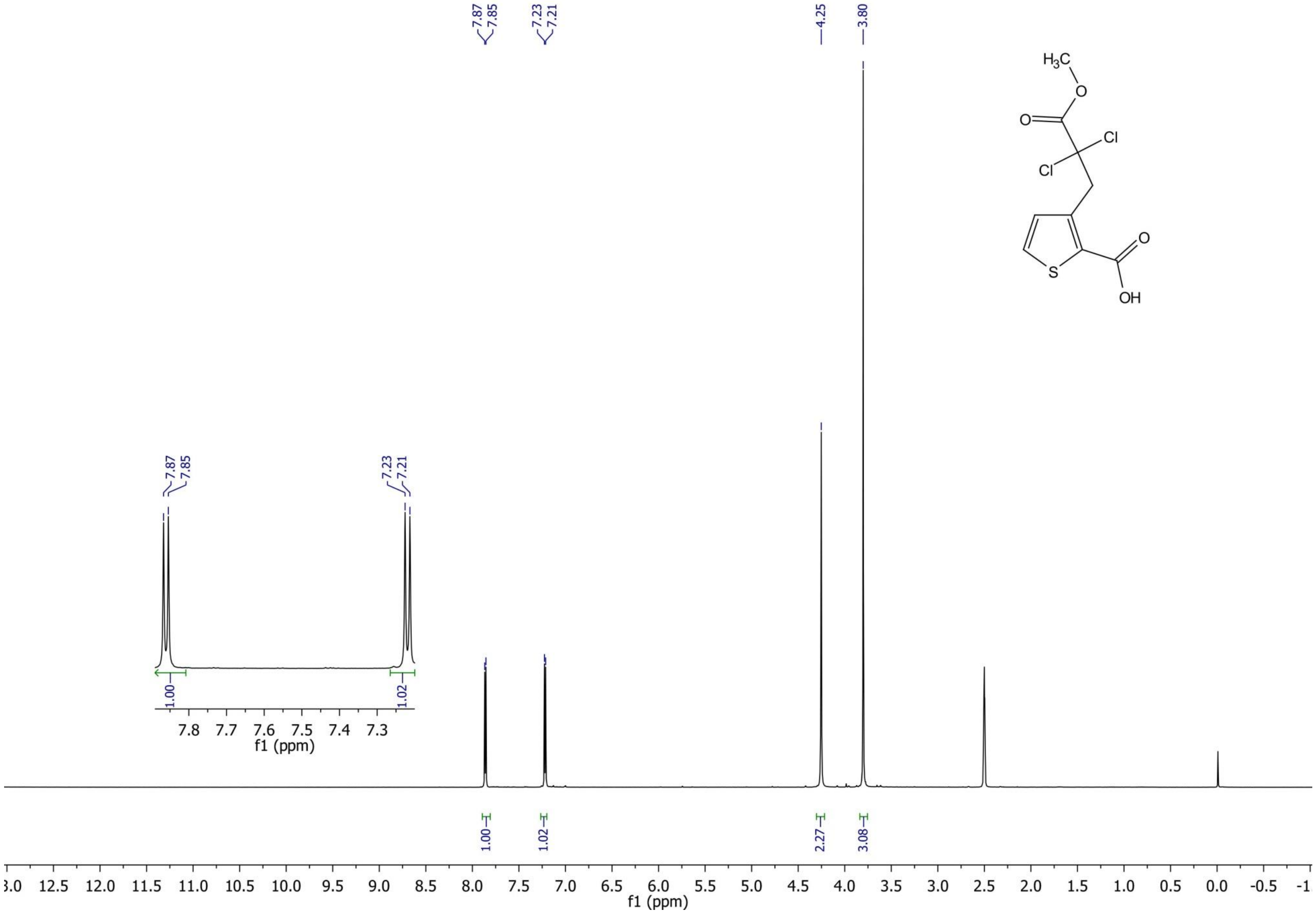

2,2-Dichloro-3-(2-(methoxycarbonyl)thiophen-3-yl)propanoic acid 17.

^{13}C NMR (101 MHz, [D$_6$]DMSO)

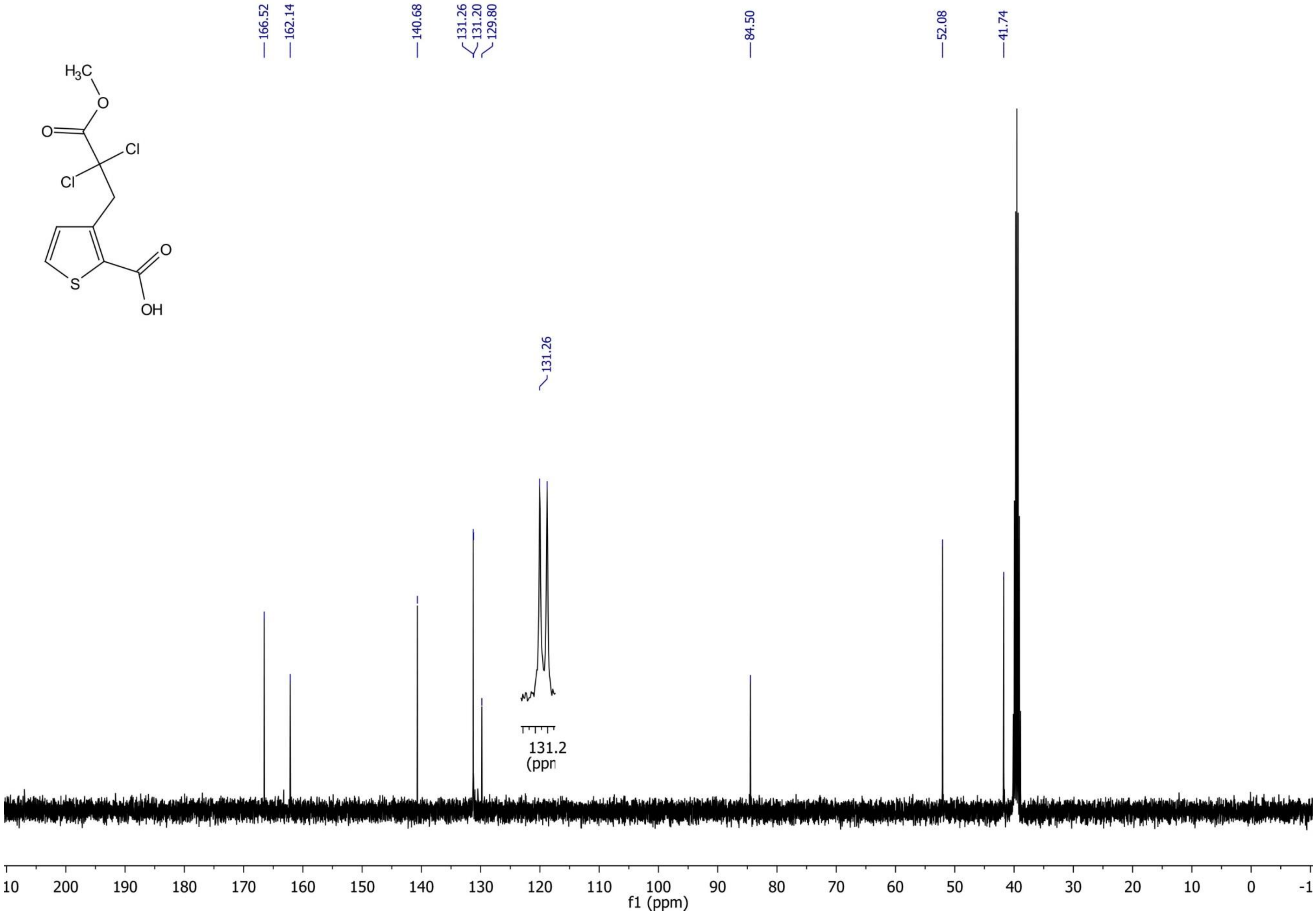

3-(2-Carboxy-2-hydroxypropyl)thiophene-2-carboxylic acid 18.

^{1}H NMR (400 MHz, $[D_6]$DMSO)

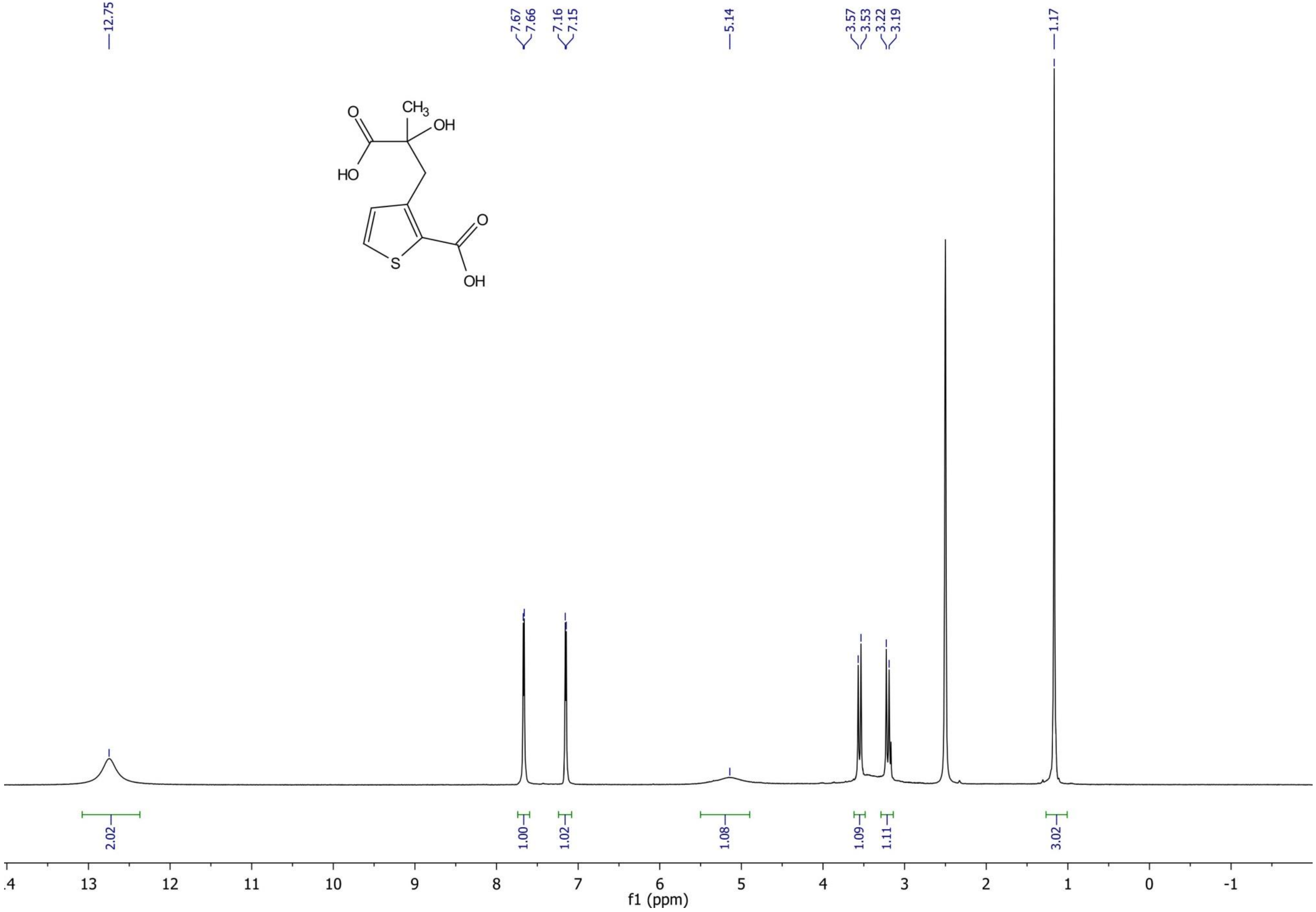

3-(2-Carboxy-2-hydroxypropyl)thiophene-2-carboxylic acid 18.

^{13}C NMR (101 MHz, [D_6]DMSO)

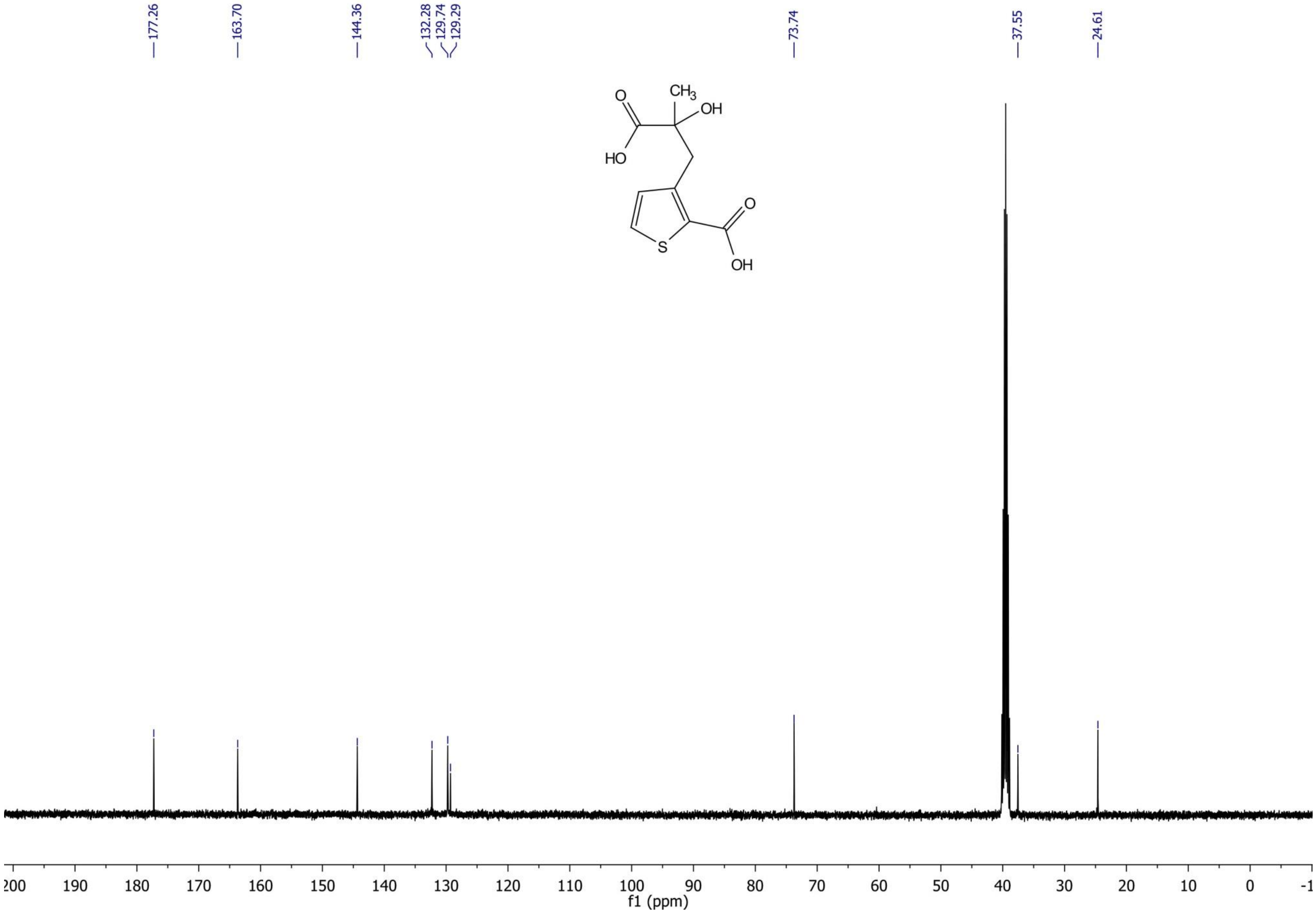

Methyl 3-((2-amino-4-methylthiazol-5-yl)methyl)thiophene-2-carboxylate 19a.

^{1}H NMR (400 MHz, $CDCl_3$)

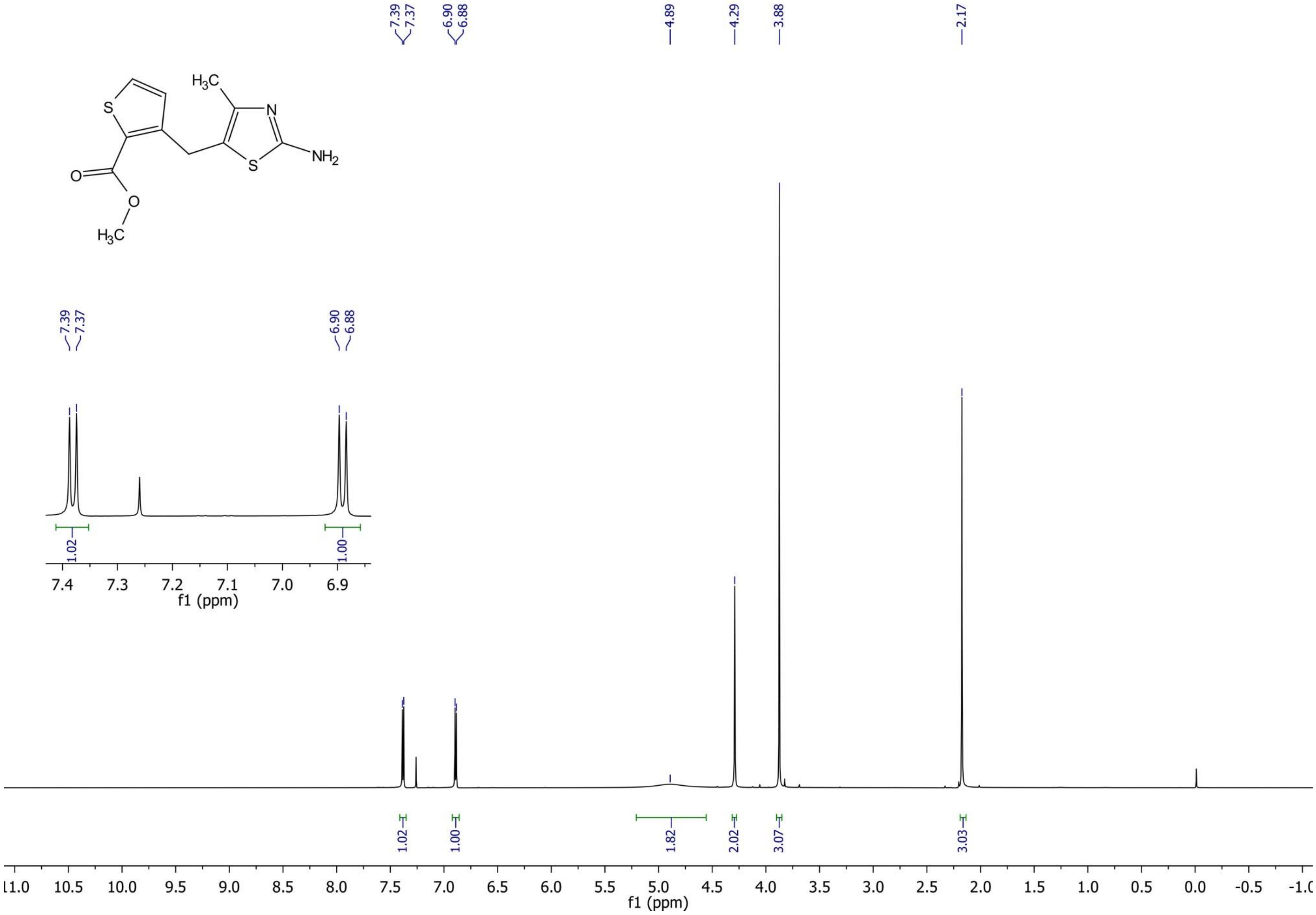

Methyl 3-((2-amino-4-methylthiazol-5-yl)methyl)thiophene-2-carboxylate 19a.

^{1}H NMR (500 MHz, [D_6]DMSO)

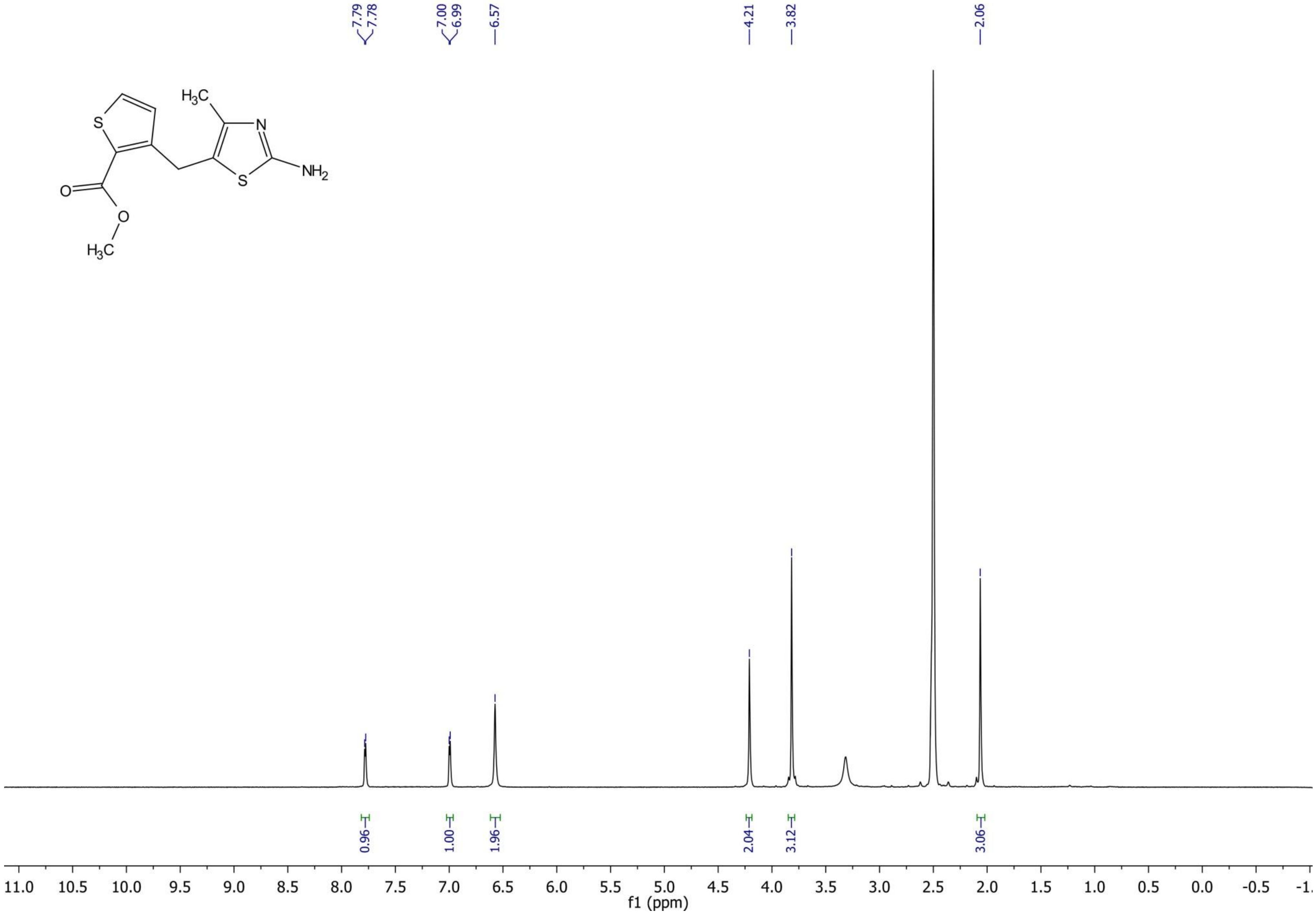

Methyl 3-((2-amino-4-methylthiazol-5-yl)methyl)thiophene-2-carboxylate 19a.

^{13}C NMR (101 MHz, $CDCl_3$)

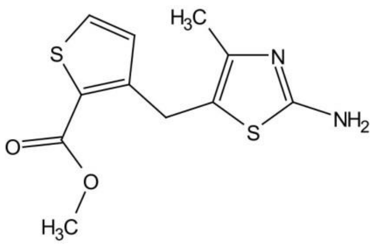

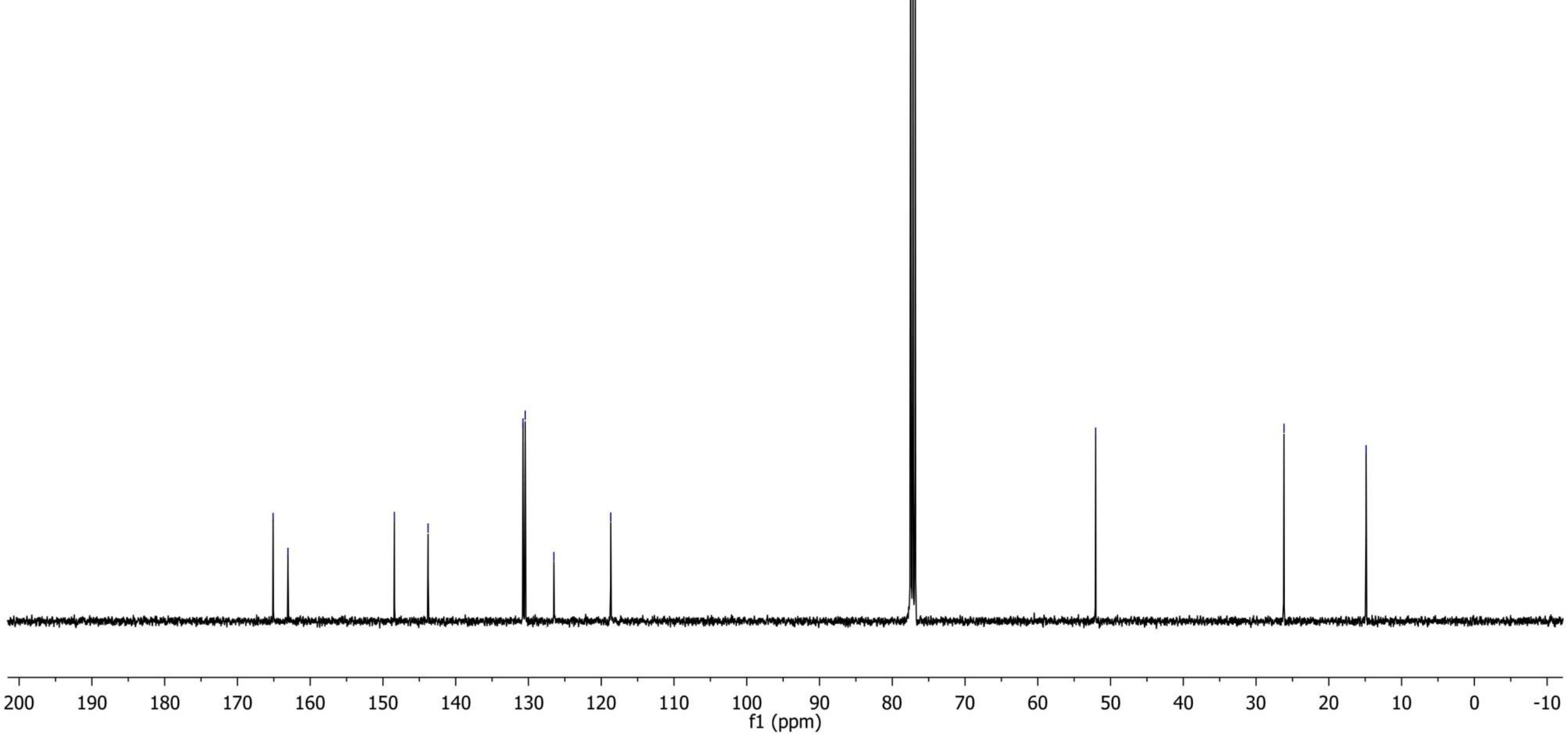

Methyl 3-((2-aminothiazol-5-yl)methyl)thiophene-2-carboxylate 19b.

^{1}H NMR (400 MHz, $[D_6]DMSO$)

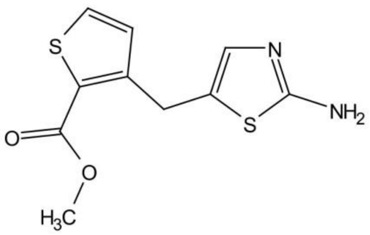

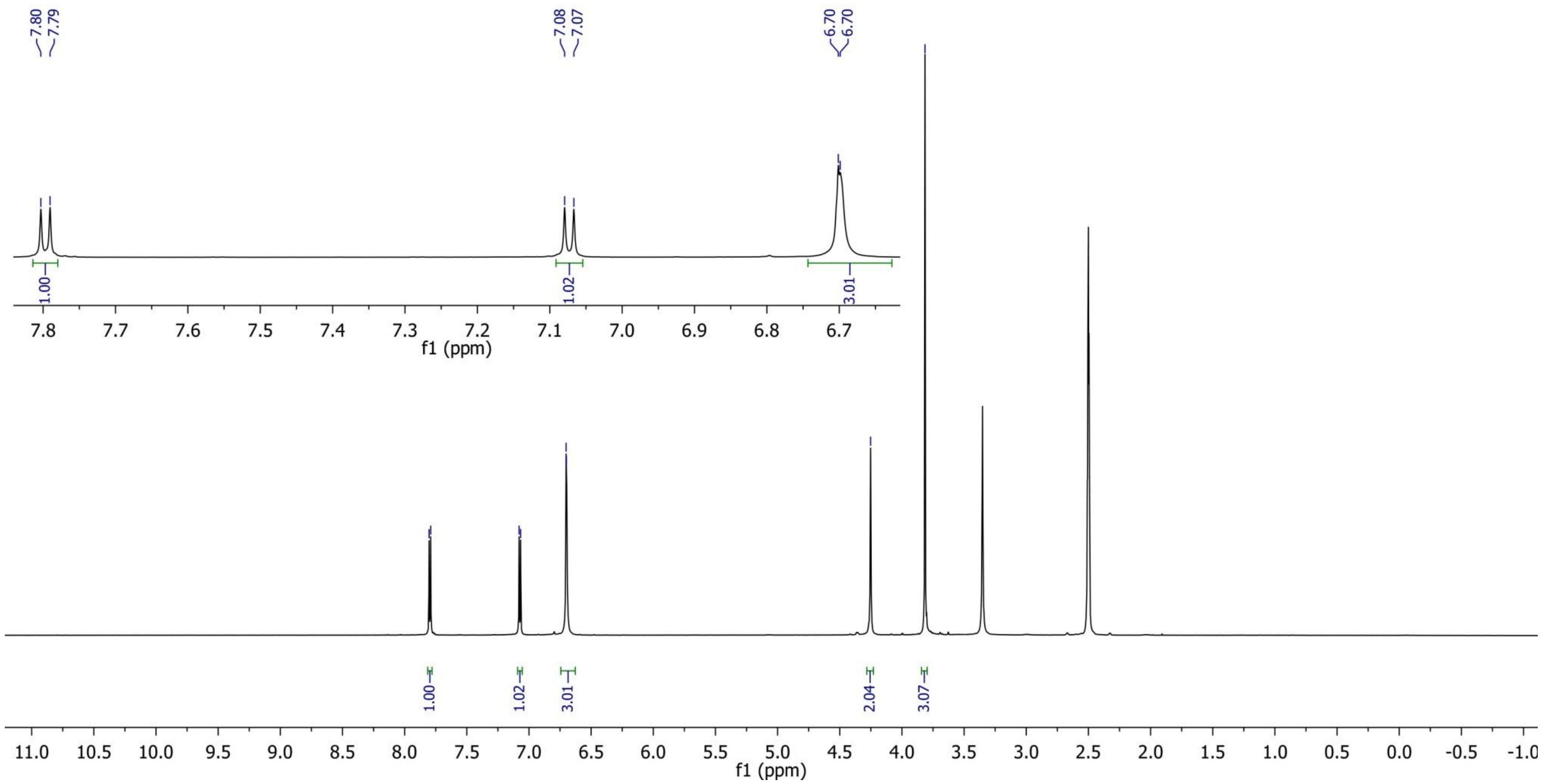

Methyl 3-((2-aminothiazol-5-yl)methyl)thiophene-2-carboxylate 19b.

^{13}C NMR (101 MHz, [D$_6$]DMSO)

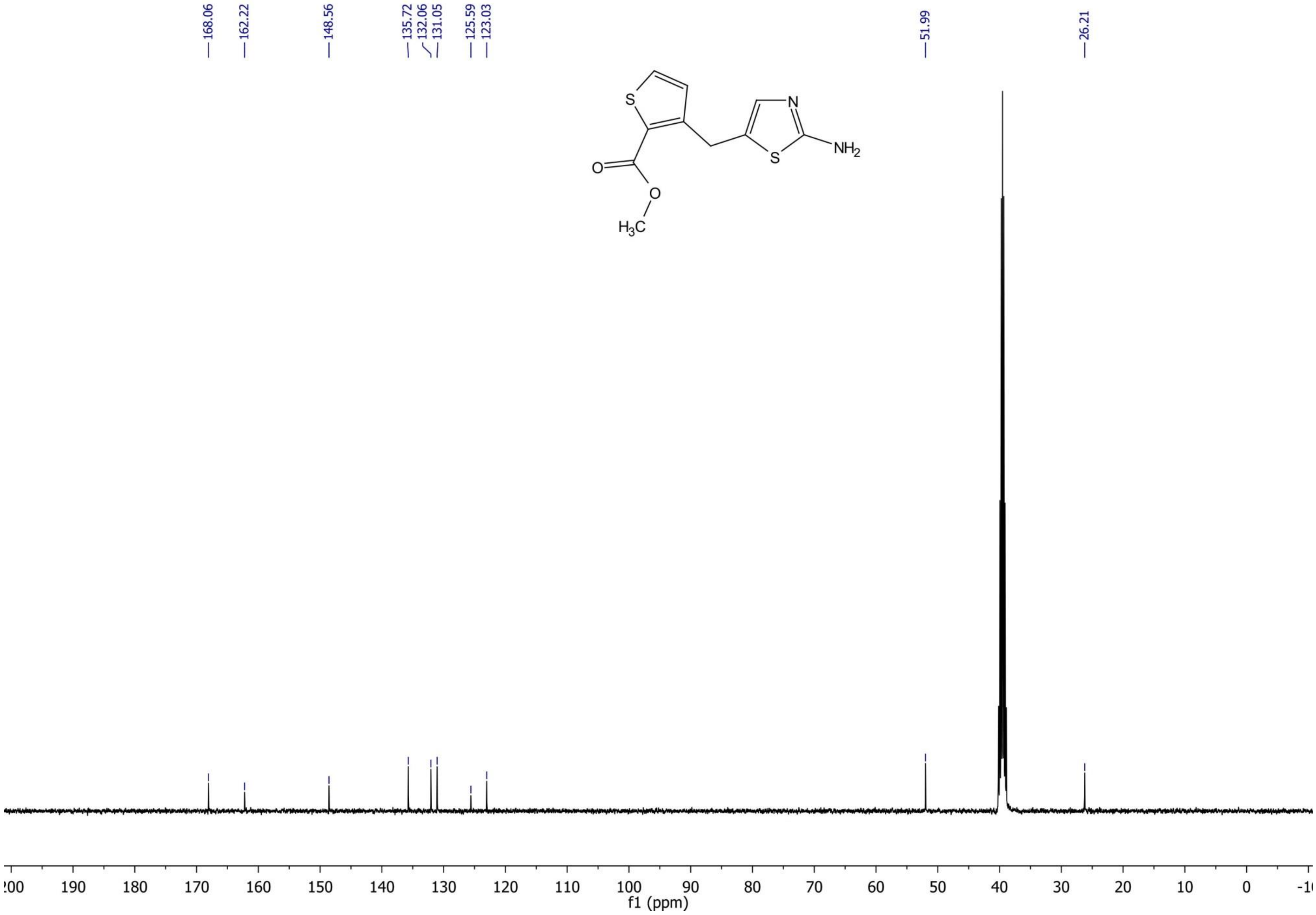

Methyl 3-((2-amino-4-methyl-1,3-selenazol-5-yl)methyl)thiophene-2-carboxylate 20a.

^{1}H NMR (400 MHz, [D$_6$]DMSO)

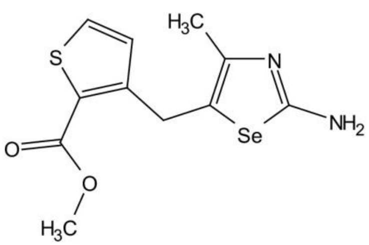

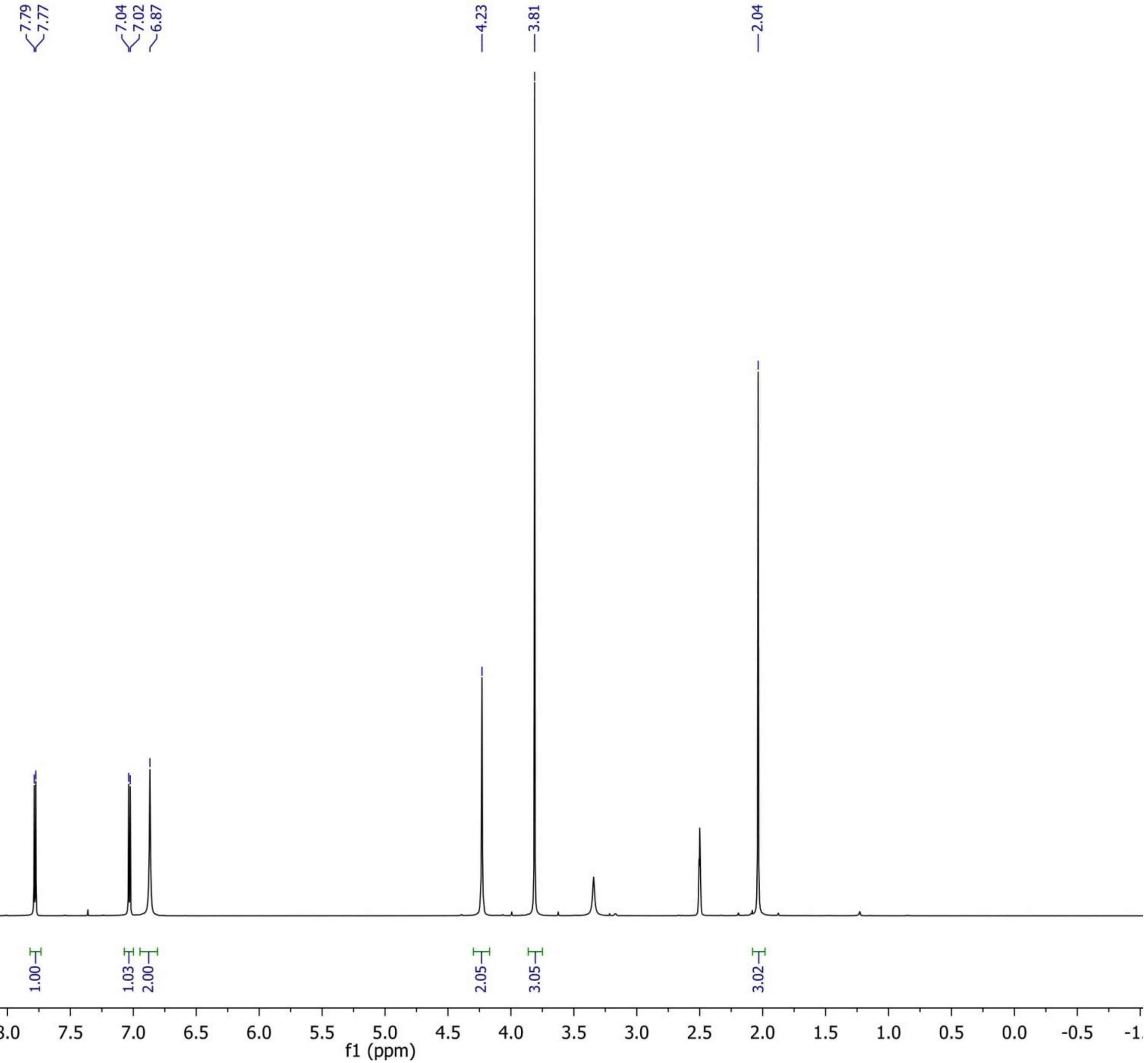

Methyl 3-((2-amino-4-methyl-1,3-selenazol-5-yl)methyl)thiophene-2-carboxylate 20a.

^{13}C NMR (101 MHz, [D$_6$]DMSO)

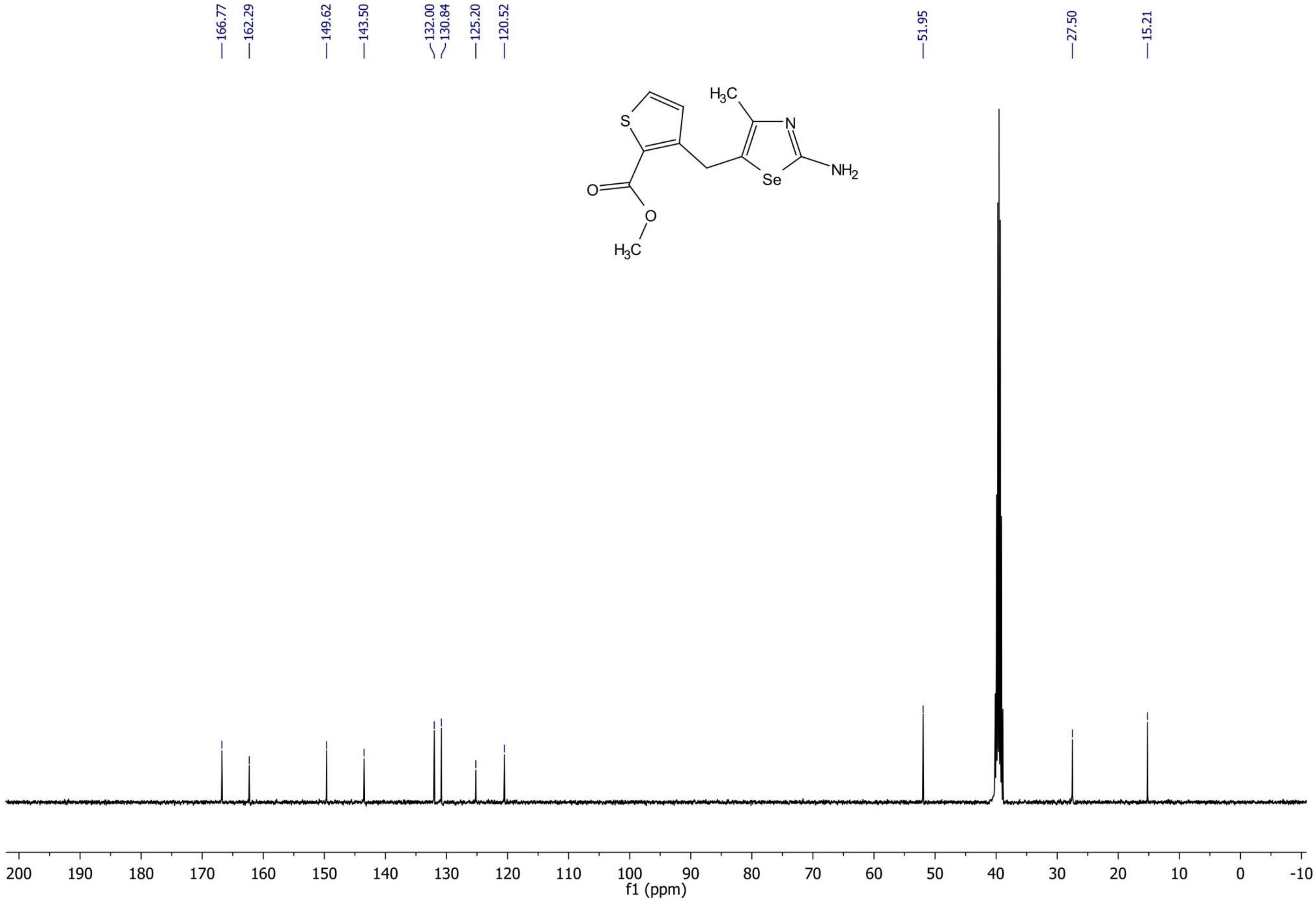

Methyl 3-((2-amino-4-methyl-1,3-selenazol-5-yl)methyl)thiophene-2-carboxylate 20a.

^{77}Se NMR (76 MHz, [D$_6$]DMSO)

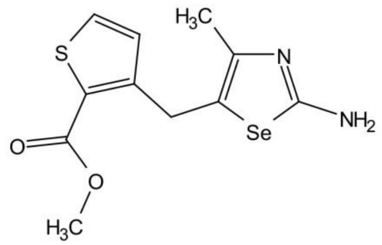

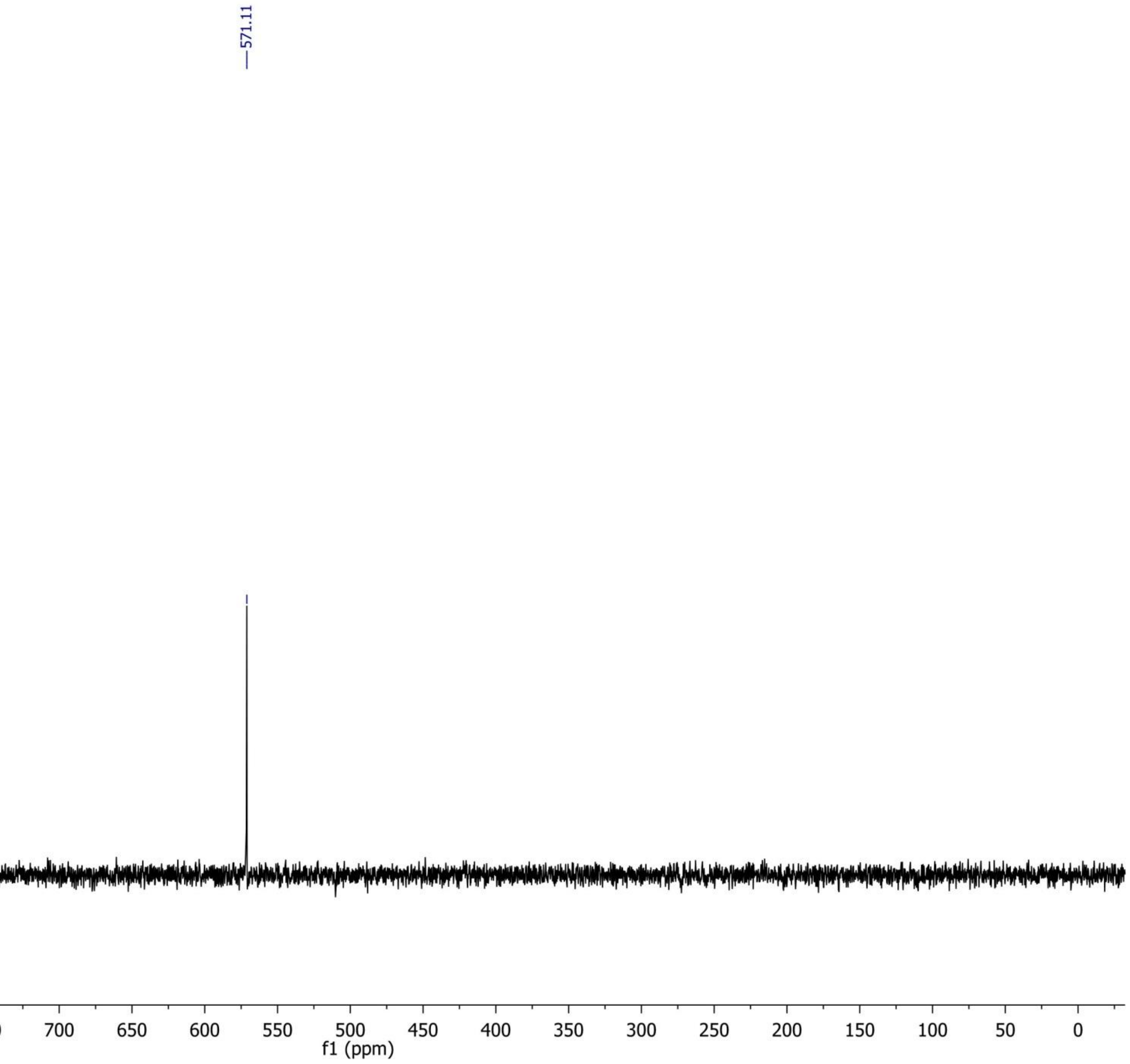

Methyl 3-((2-amino-1,3-selenazol-5-yl)methyl)thiophene-2-carboxylate 20b.

^{1}H NMR (400 MHz, [D_6]DMSO)

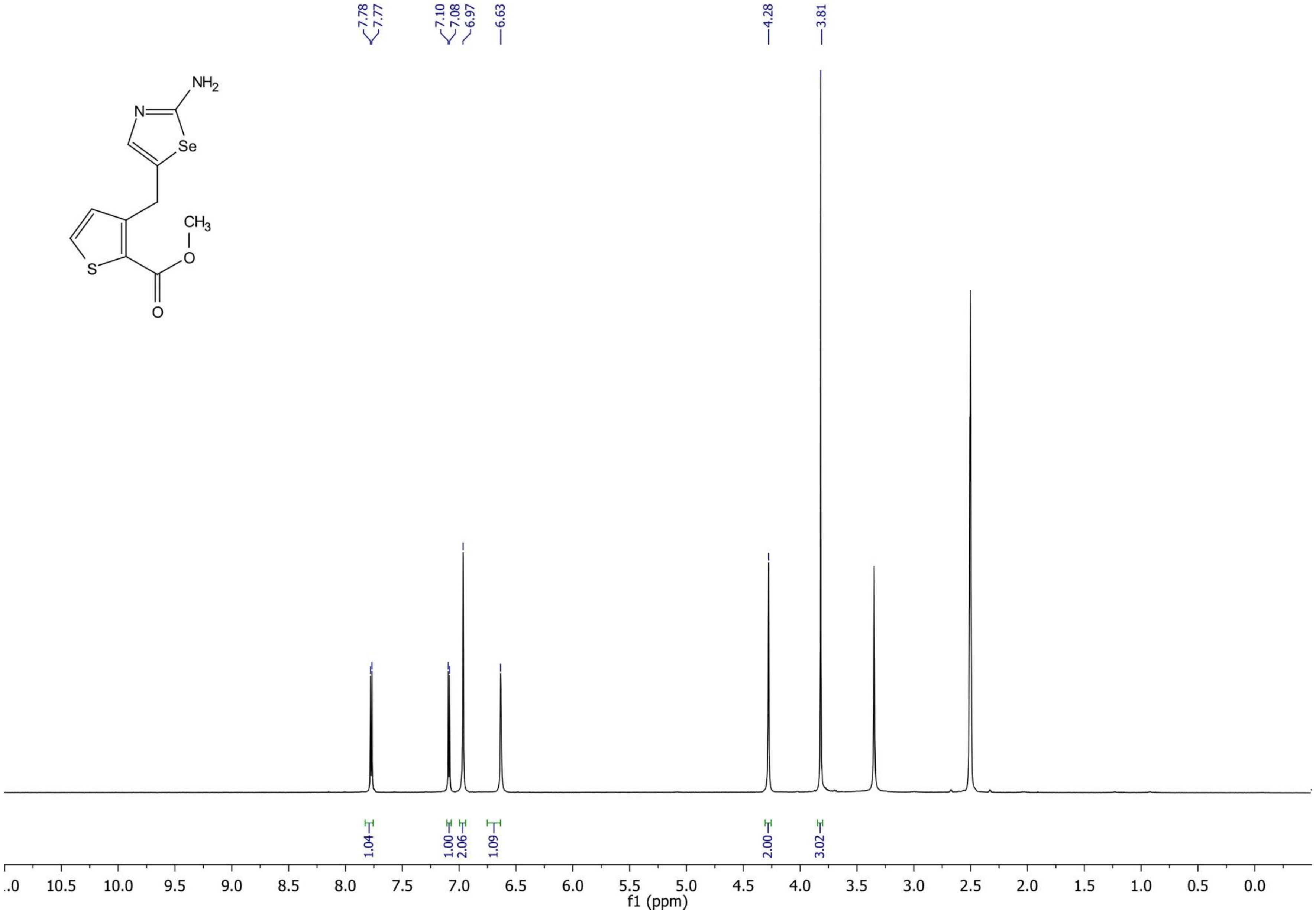

Methyl 3-((2-amino-1,3-selenazol-5-yl)methyl)thiophene-2-carboxylate 20b.

^{13}C NMR (101 MHz, [D$_6$]DMSO)

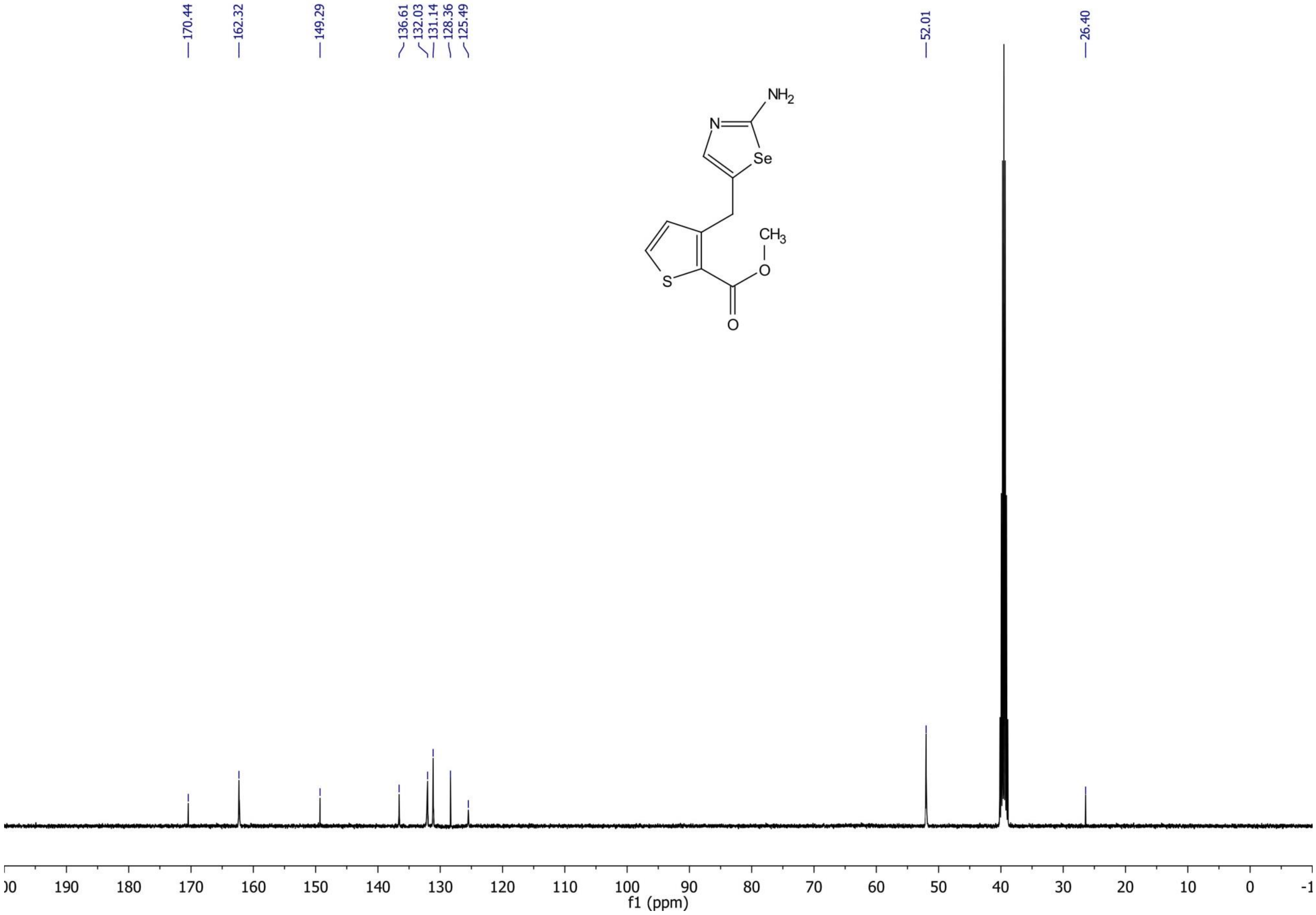

Methyl 3-((2-imino-4-oxothiazolidin-5-yl)methyl)thiophene-2-carboxylate hydrobromide 21.

^{1}H NMR (400 MHz, [D$_6$]DMSO)

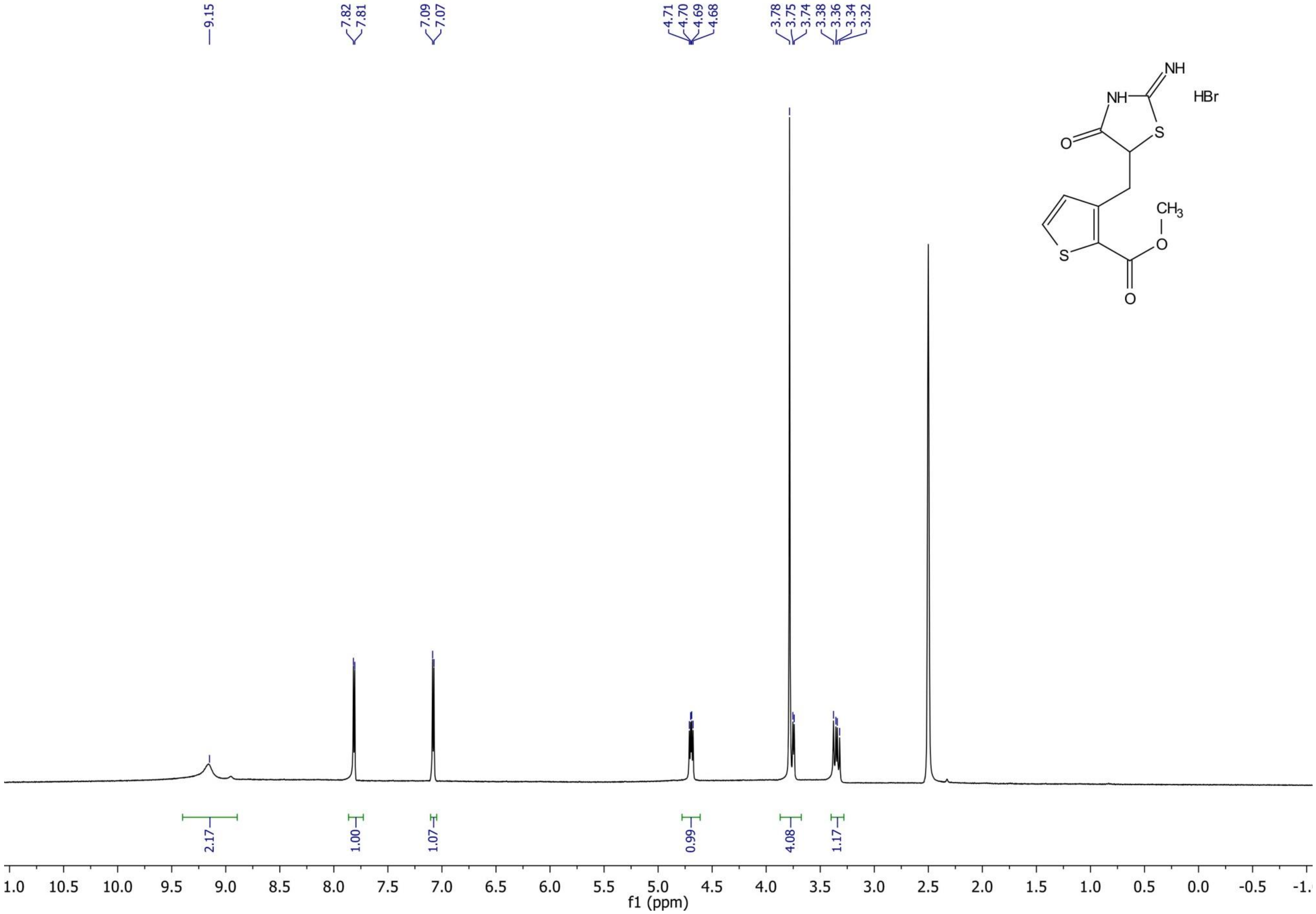

Methyl 3-((2-imino-4-oxothiazolidin-5-yl)methyl)thiophene-2-carboxylate hydrobromide 21.

^{13}C NMR (101 MHz, [D$_6$]DMSO)

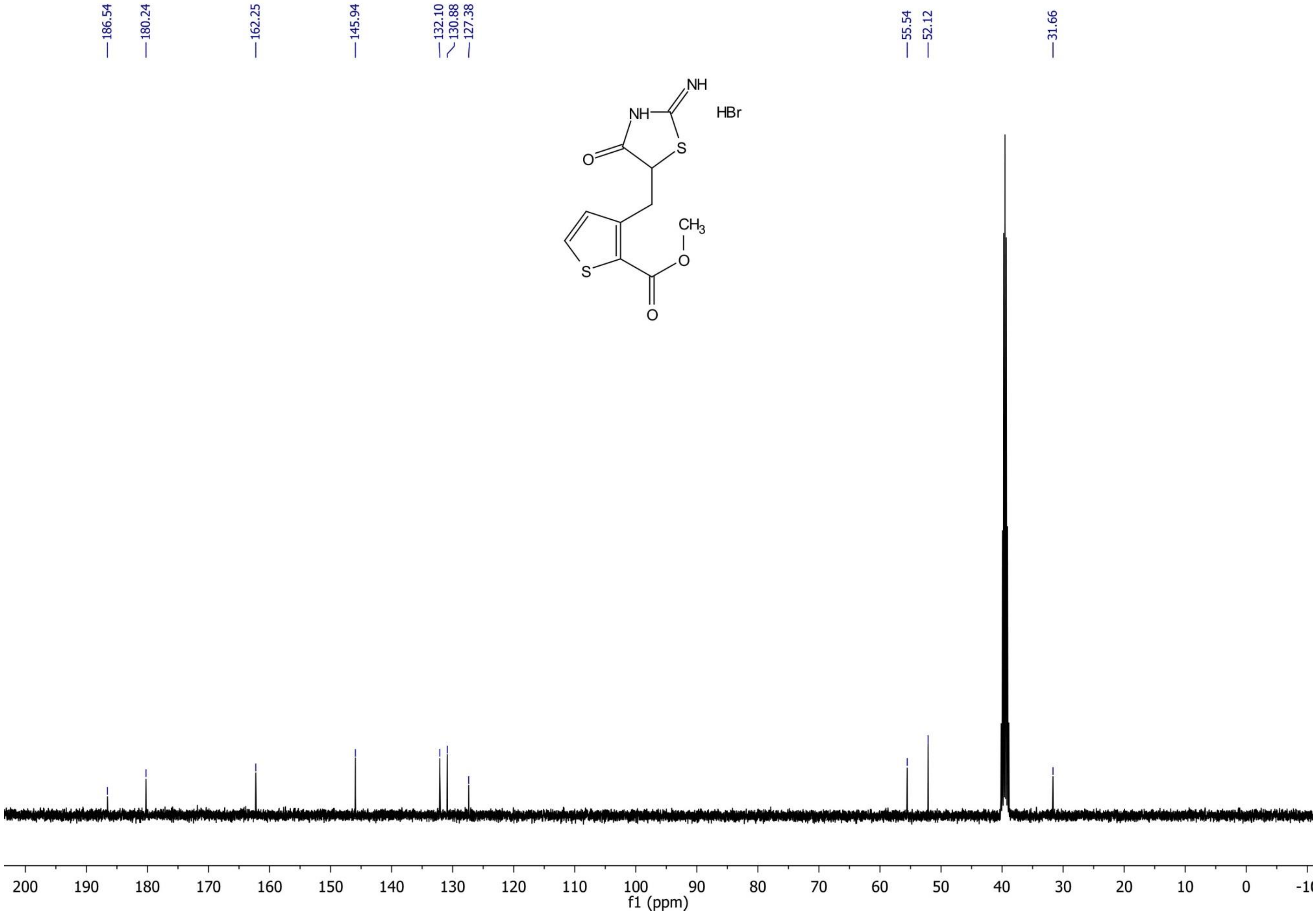

Methyl 3-((2,4-diiminothiazolidin-5-yl)methyl)thiophene-2-carboxylate hydrobromide 22.

^{1}H NMR (400 MHz, [D$_6$]DMSO)

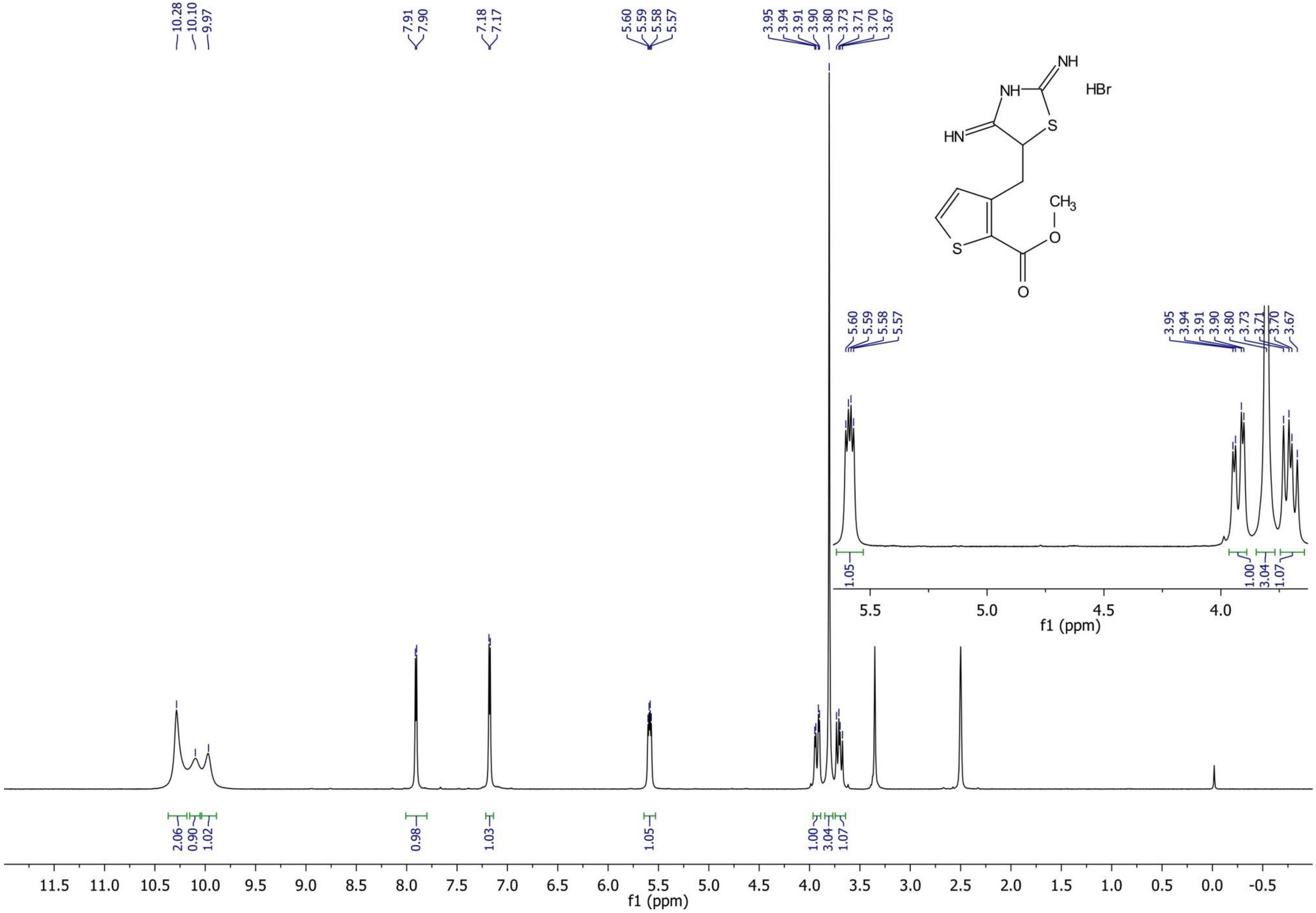

Methyl 3-((2,4-diiminothiazolidin-5-yl)methyl)thiophene-2-carboxylate hydrobromide 22.

^{13}C NMR (101 MHz, $[D_6]DMSO$)

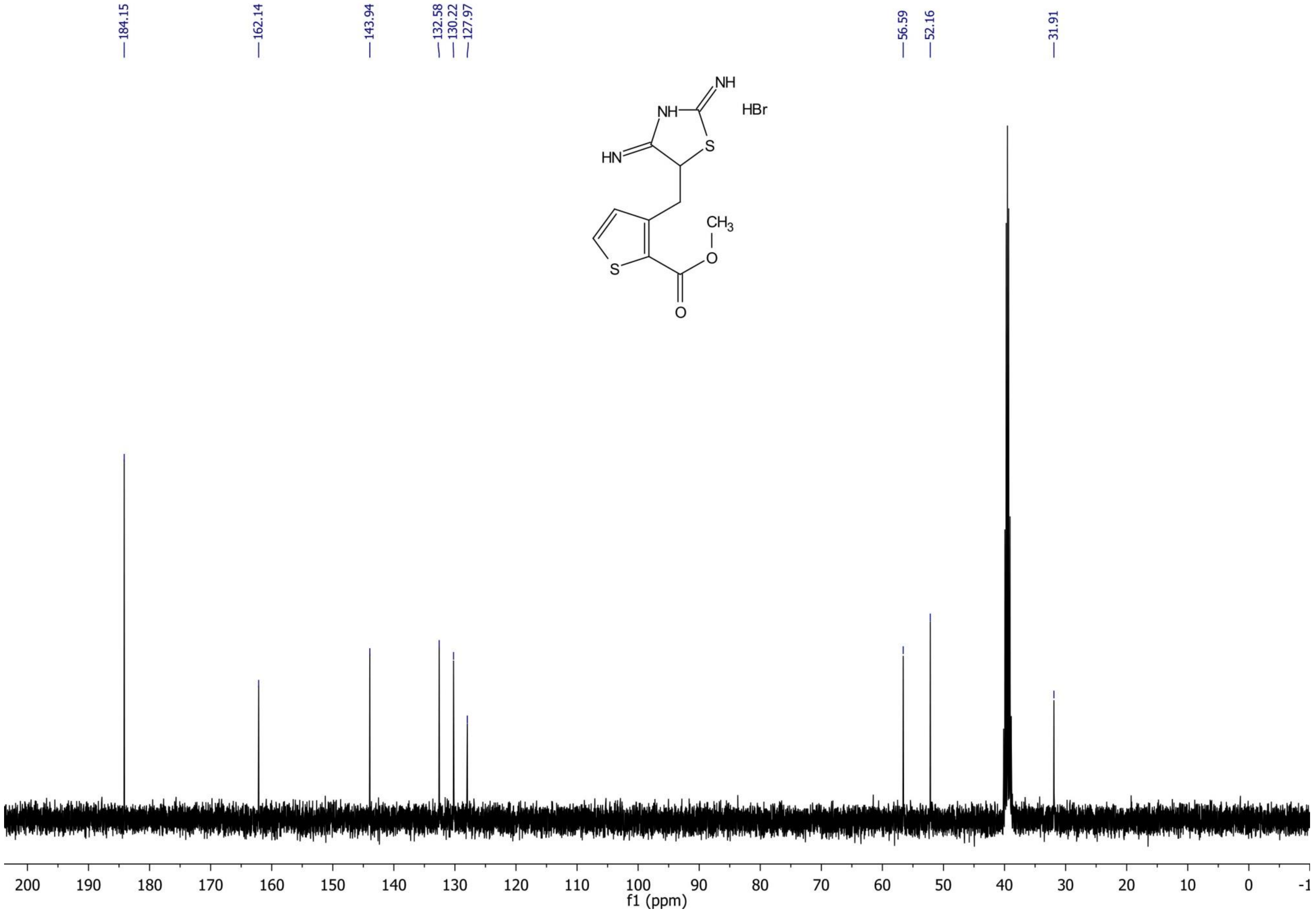

Methyl 3-((2-imino-4-oxo-1,3-selenazolidin-5-yl)methyl)thiophene-2-carboxylate hydrobromide 23.

^{1}H NMR (400 MHz, [D$_6$]DMSO)

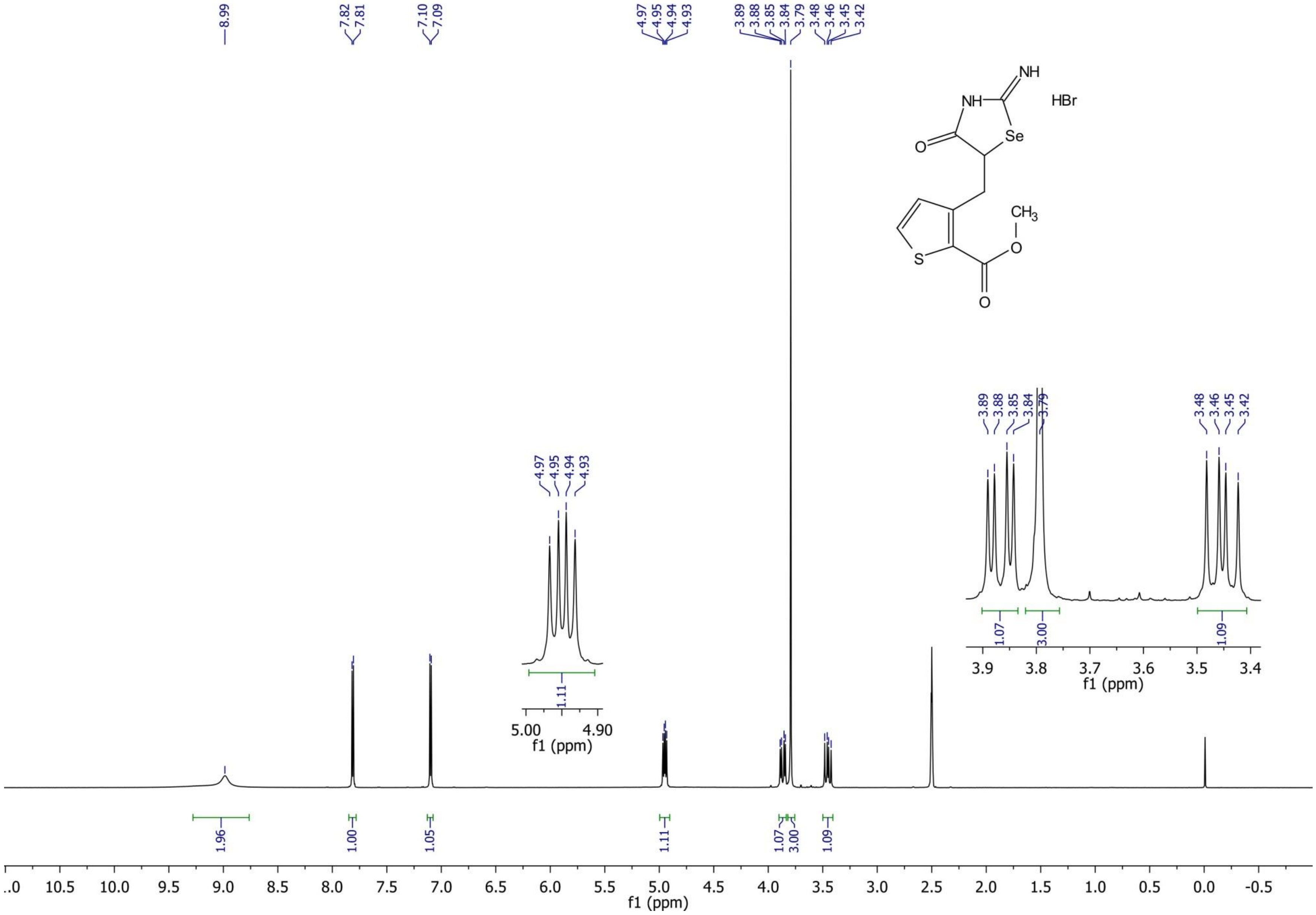

Methyl 3-((2-imino-4-oxo-1,3-selenazolidin-5-yl)methyl)thiophene-2-carboxylate hydrobromide 23.

^{13}C NMR (101 MHz, [D$_6$]DMSO)

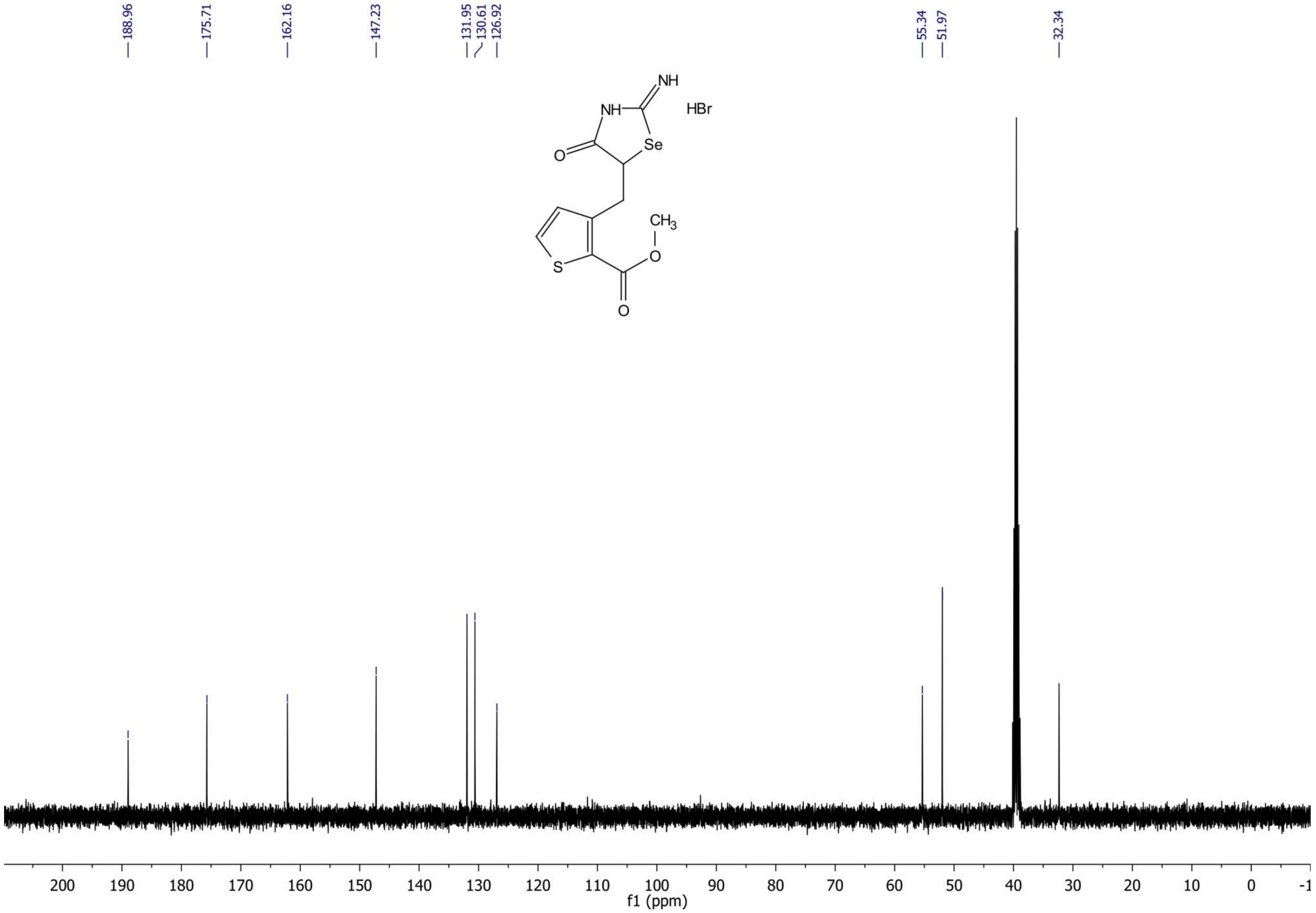

Methyl 3-((2,4-diimino-1,3-selenazolidin-5-yl)methyl)thiophene-2-carboxylate hydrobromide 24.

^{1}H NMR (400 MHz, [D$_6$]DMSO)

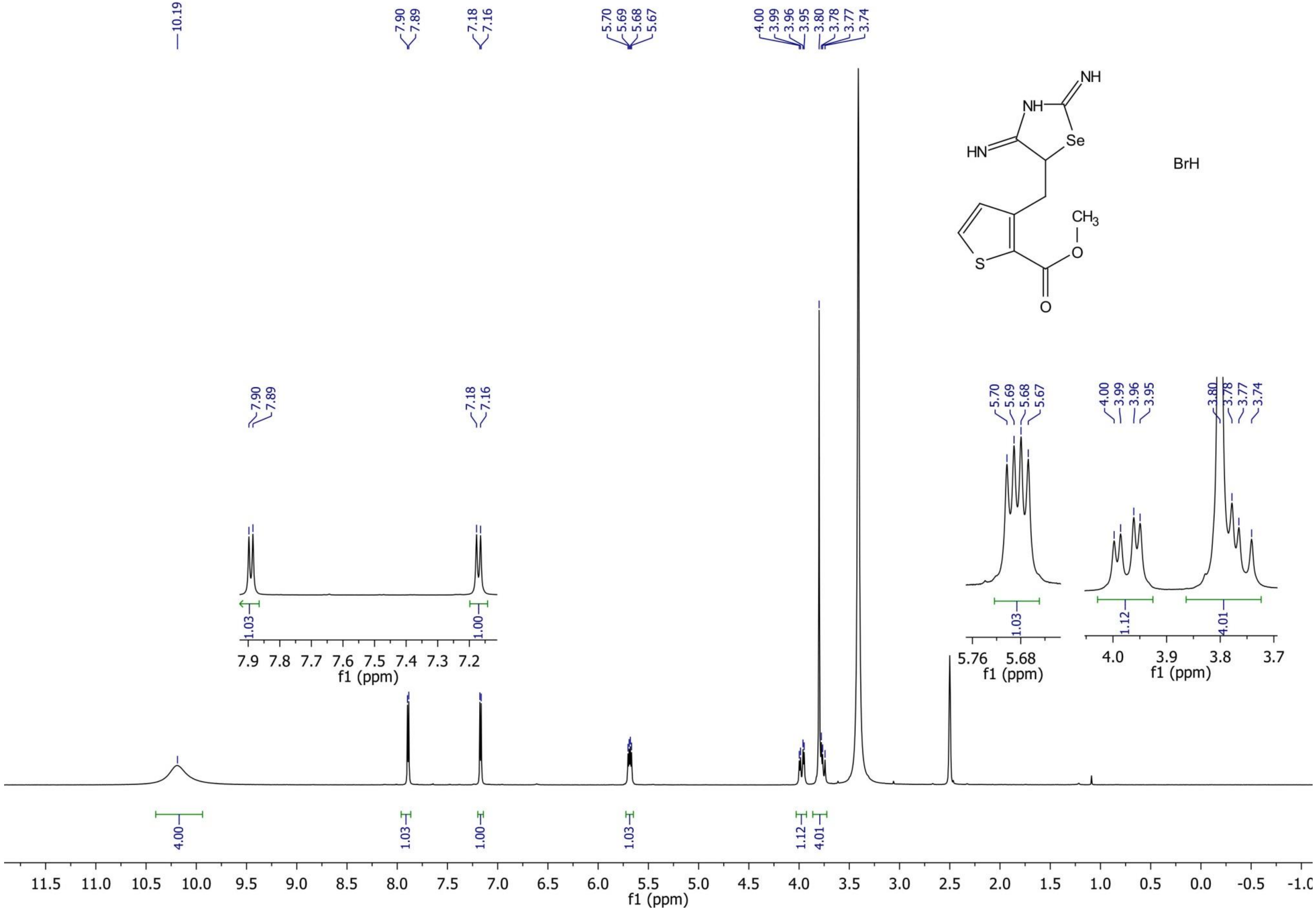

Methyl 3-((2,4-diimino-1,3-selenazolidin-5-yl)methyl)thiophene-2-carboxylate hydrobromide 24.

^{13}C NMR (101 MHz, [D$_6$]DMSO)

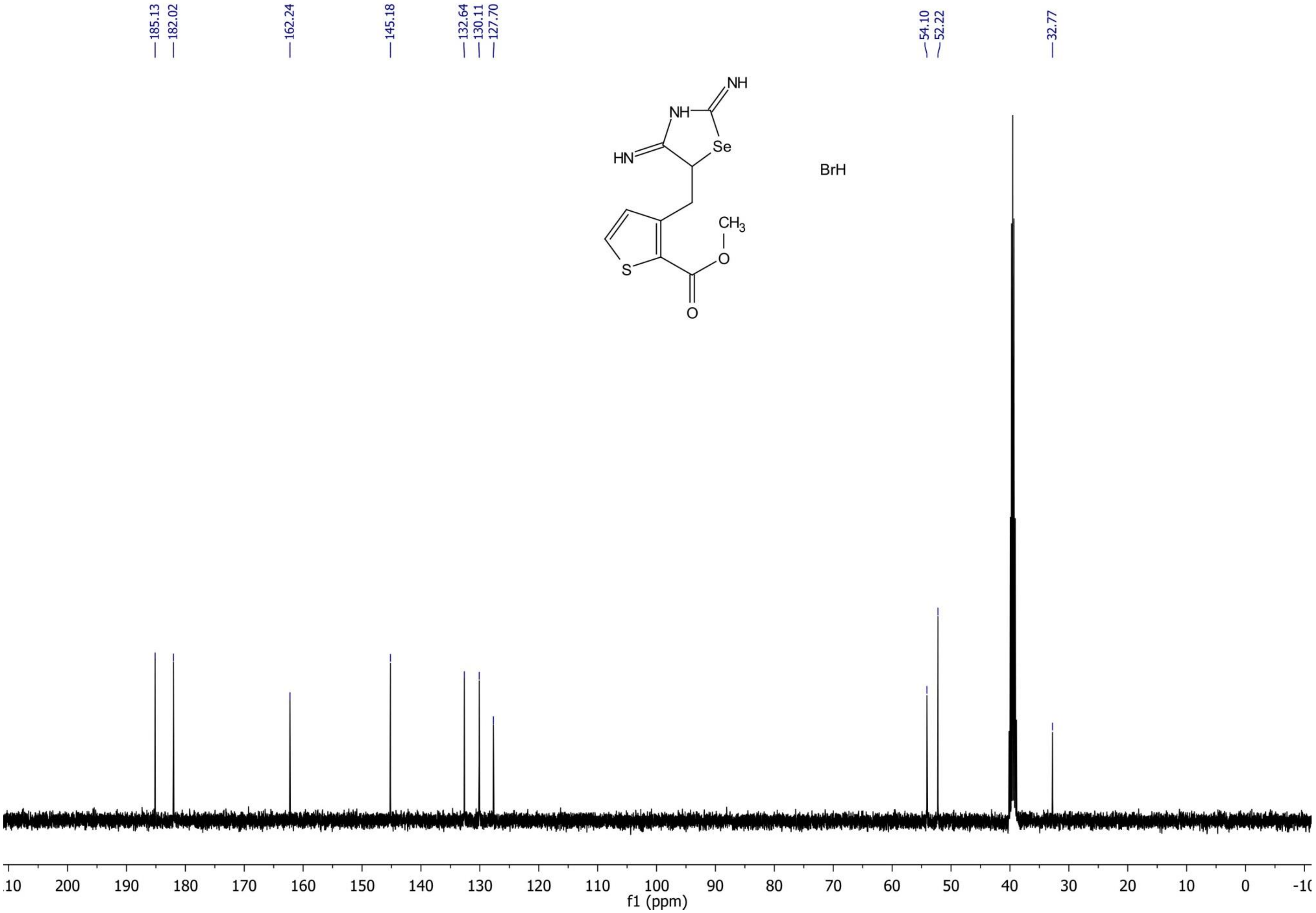

Dimethyl 4-amino-[2,3'-bithiophene]-2',5-dicarboxylate 26a.

^{1}H NMR (400 MHz, [D_6]DMSO)

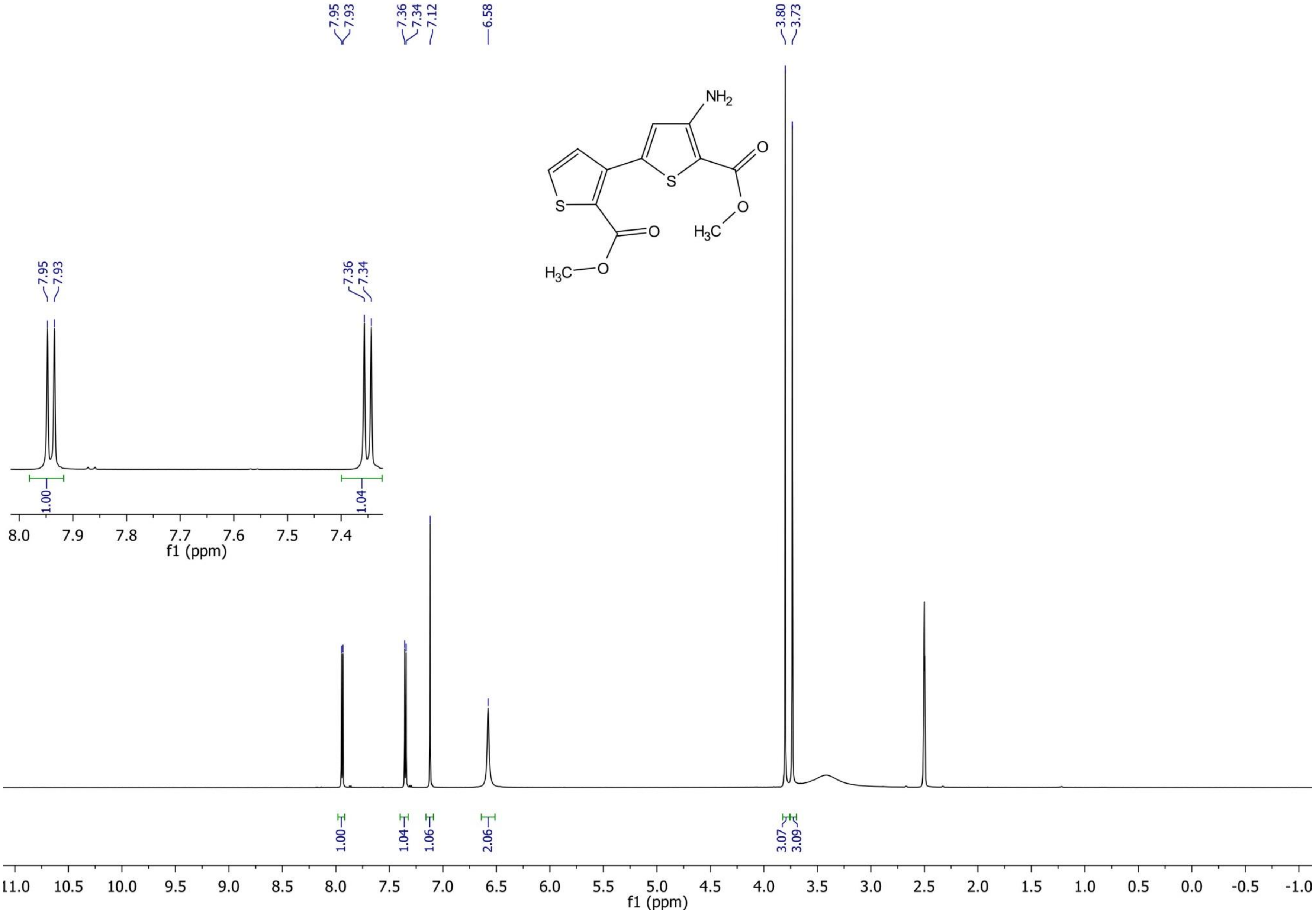

Dimethyl 4-amino-[2,3'-bithiophene]-2',5-dicarboxylate 26a.

^{13}C NMR (101 MHz, [D$_6$]DMSO)

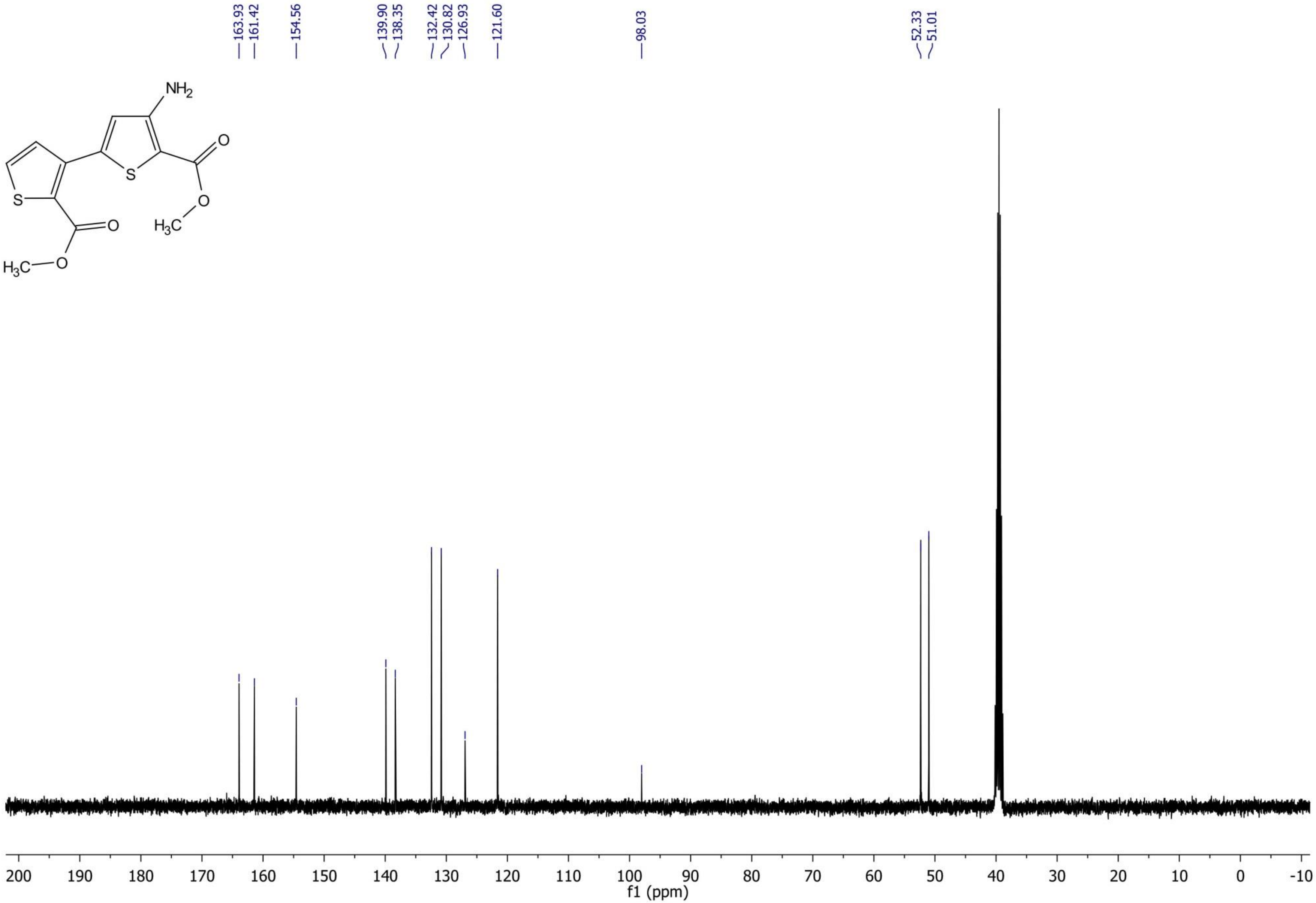

Methyl 4-amino-5-(phenylcarbamoyl)-[2,3'-bithiophene]-2'-carboxylate 26b.

^{1}H NMR (400 MHz, [D$_6$]DMSO)

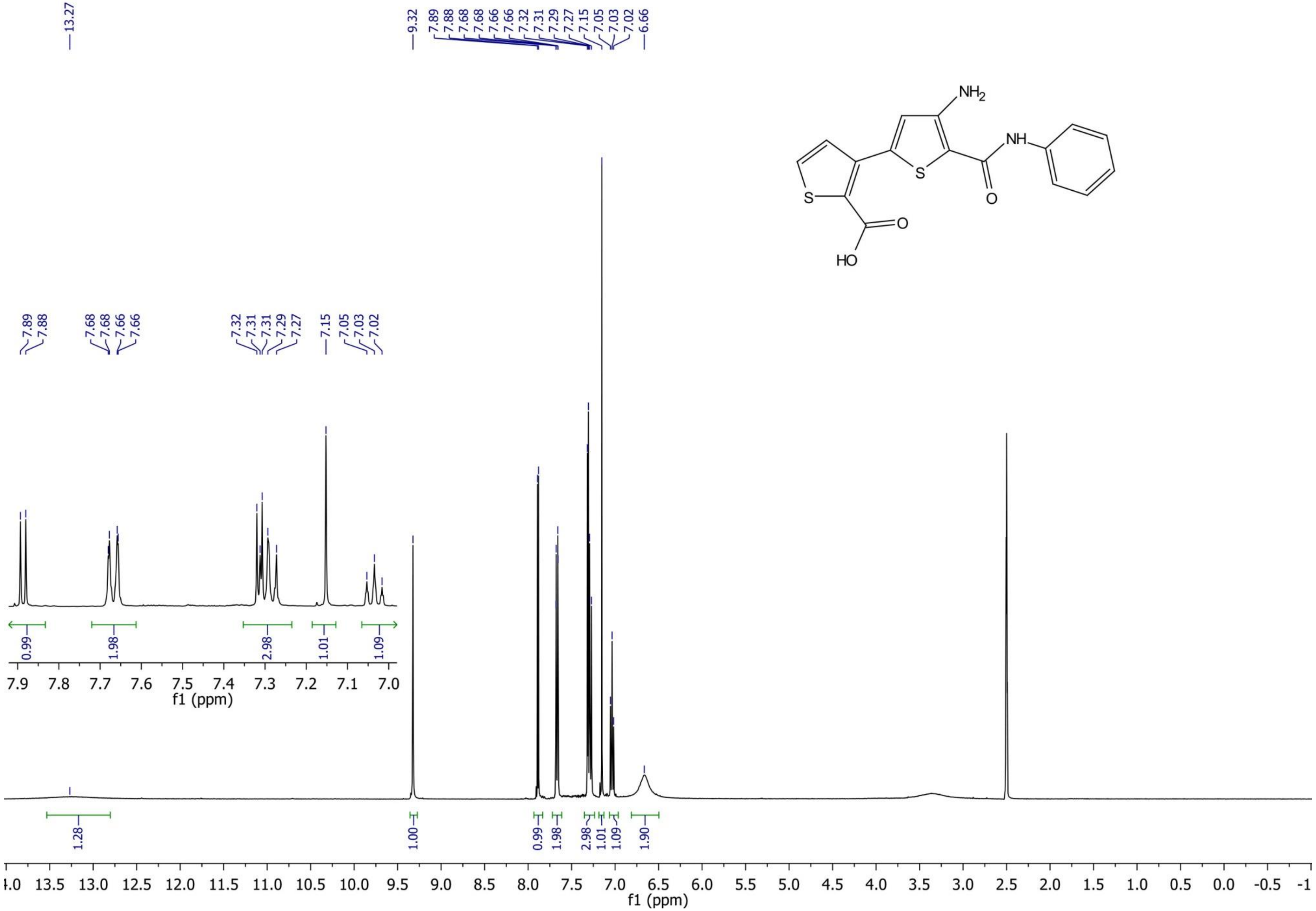

Methyl 4-amino-5-(phenylcarbamoyl)-[2,3'-bithiophene]-2'-carboxylate 26b.

^{13}C NMR (101 MHz, $[D_6]DMSO$)

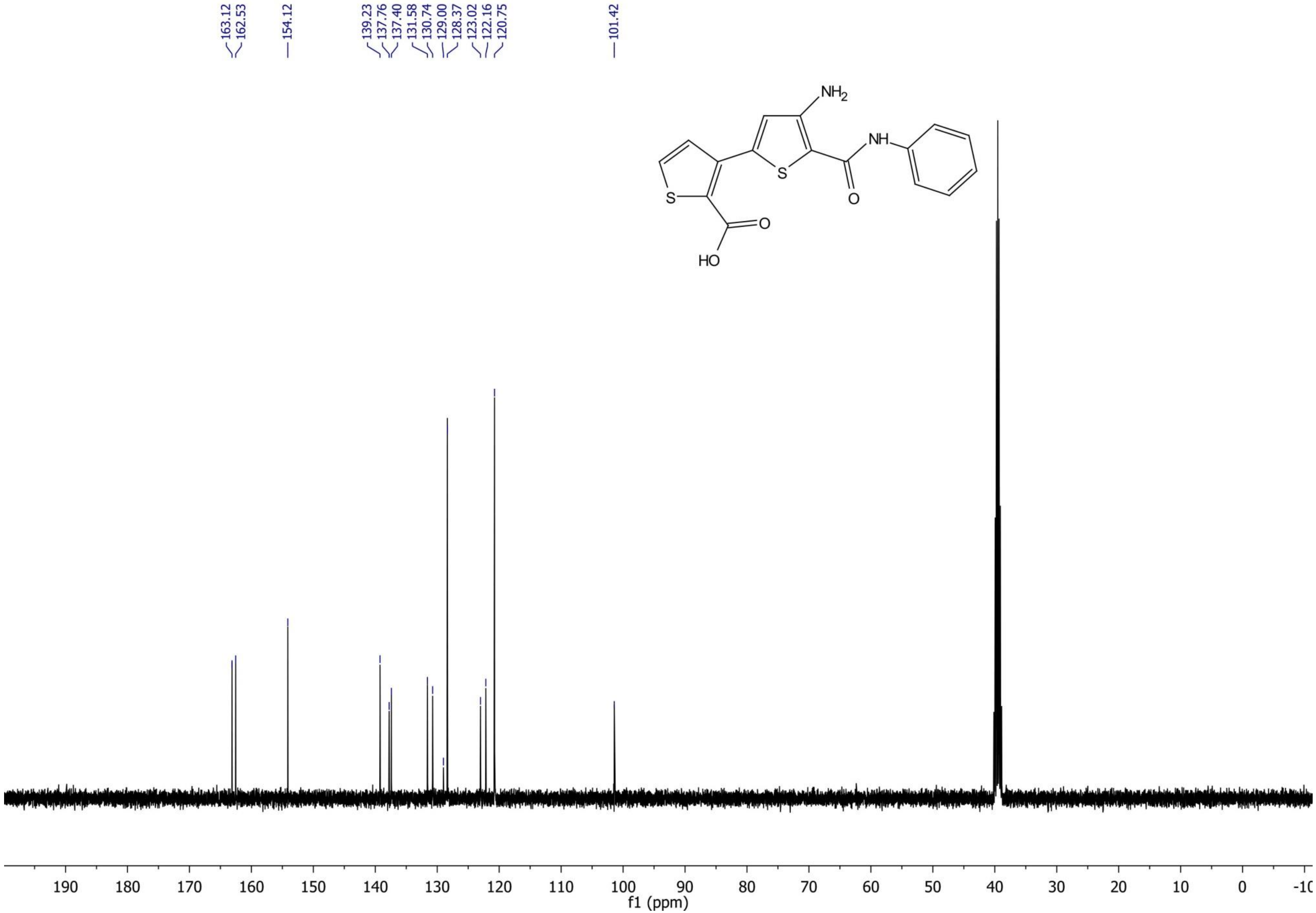

Methyl 5-acetyl-4-amino-[2,3'-bithiophene]-2'-carboxylate hydrochloride 26c.

^{1}H NMR (400 MHz, [D$_6$]DMSO)

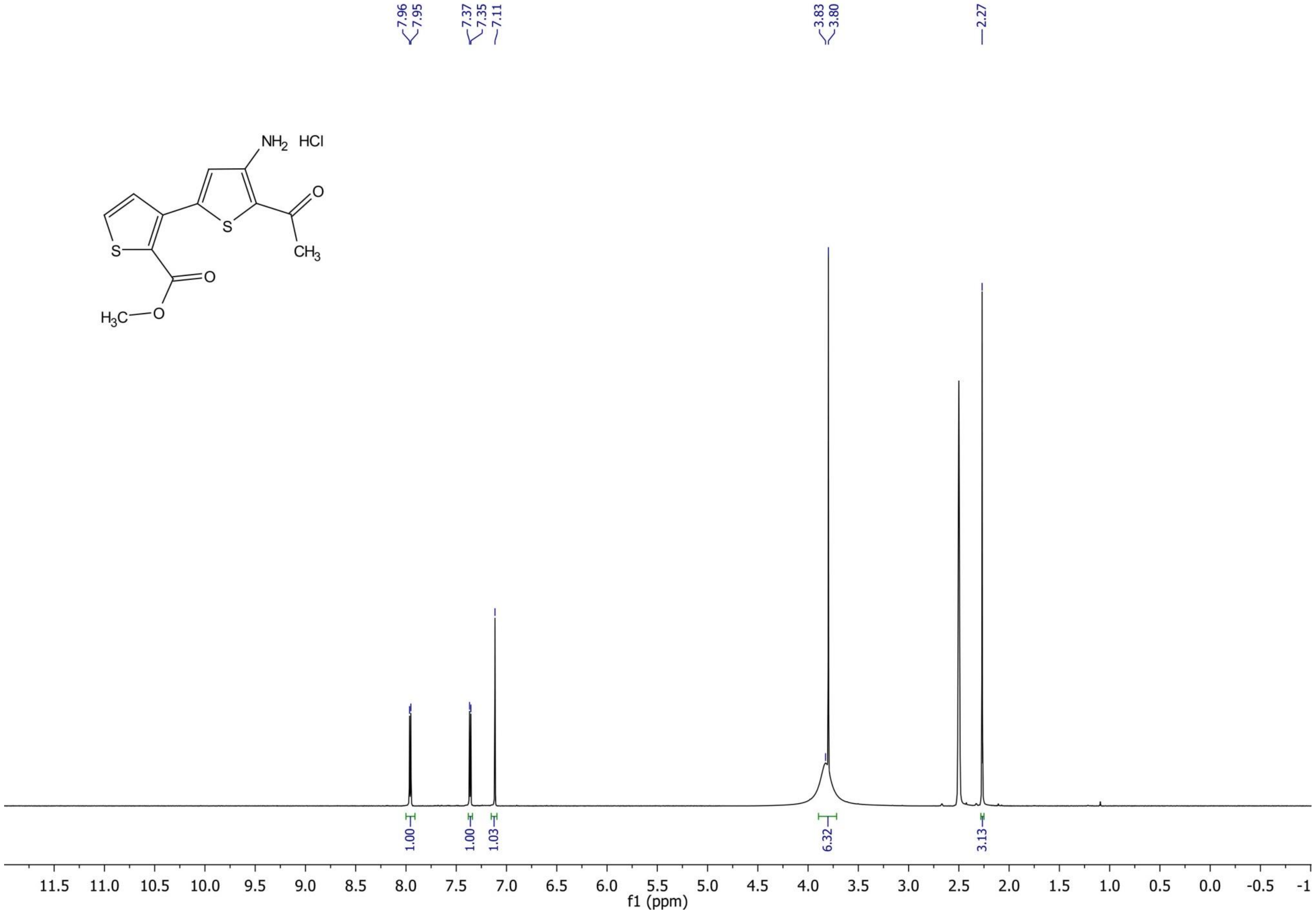

Methyl 5-acetyl-4-amino-[2,3'-bithiophene]-2'-carboxylate hydrochloride 26c.

^{13}C NMR (101 MHz, [D$_6$]DMSO)

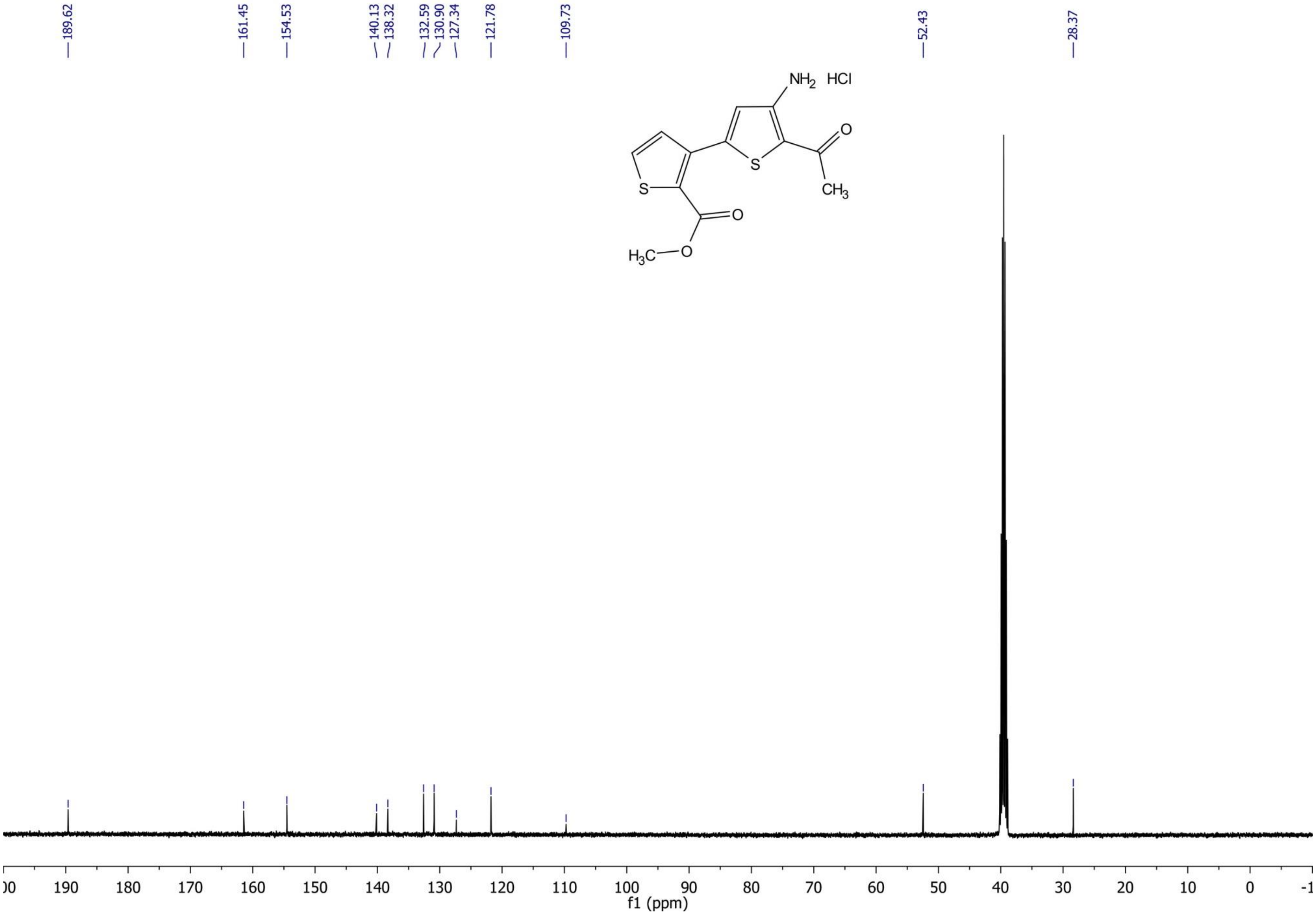

Dimethyl 4-hydroxy-[2,3'-bithiophene]-2',5-dicarboxylate 27a.

^{1}H NMR (400 MHz, [D_6]DMSO)

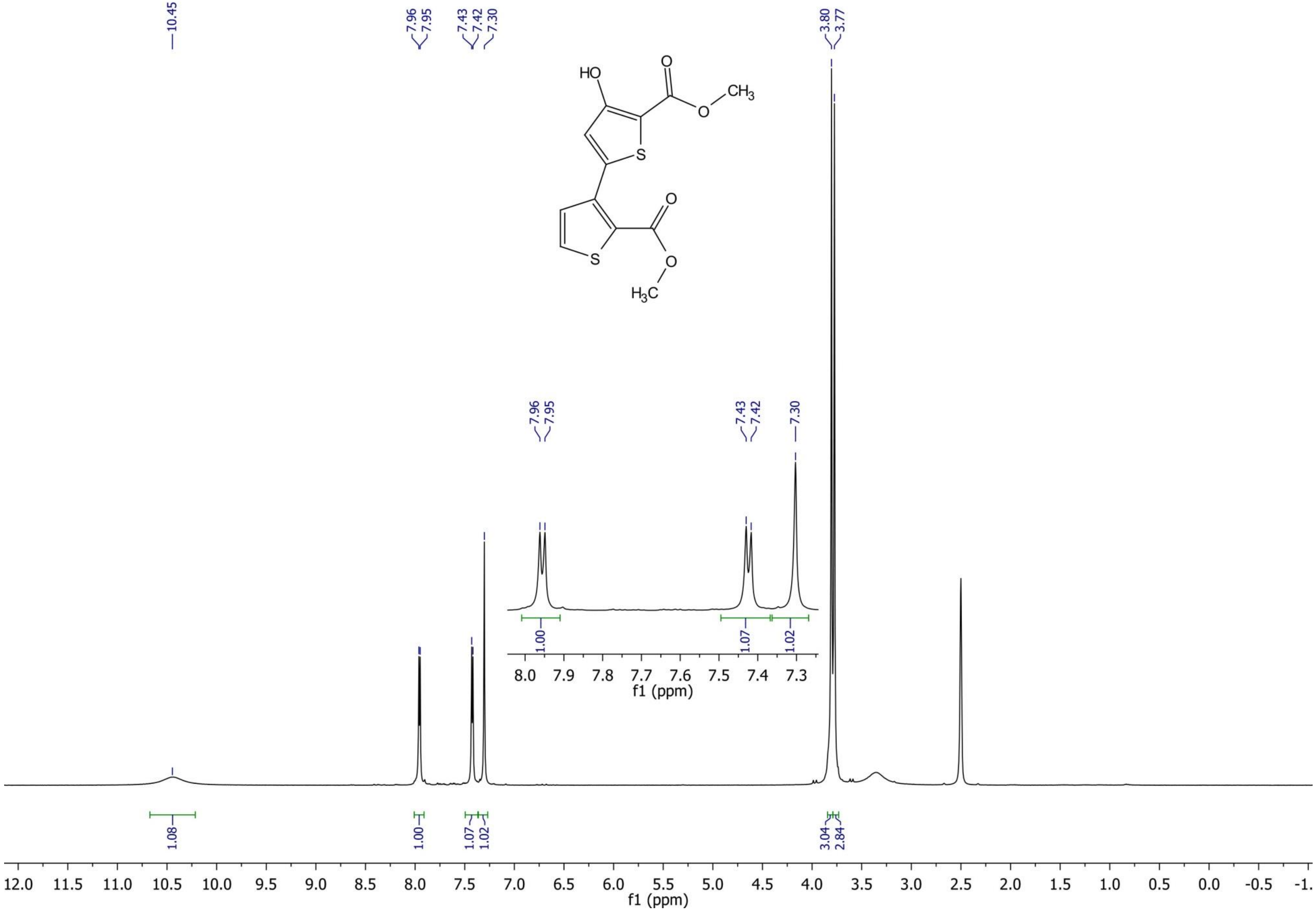

Dimethyl 4-hydroxy-[2,3'-bithiophene]-2',5-dicarboxylate 27a.

^{13}C NMR (101 MHz, [D$_6$]DMSO)

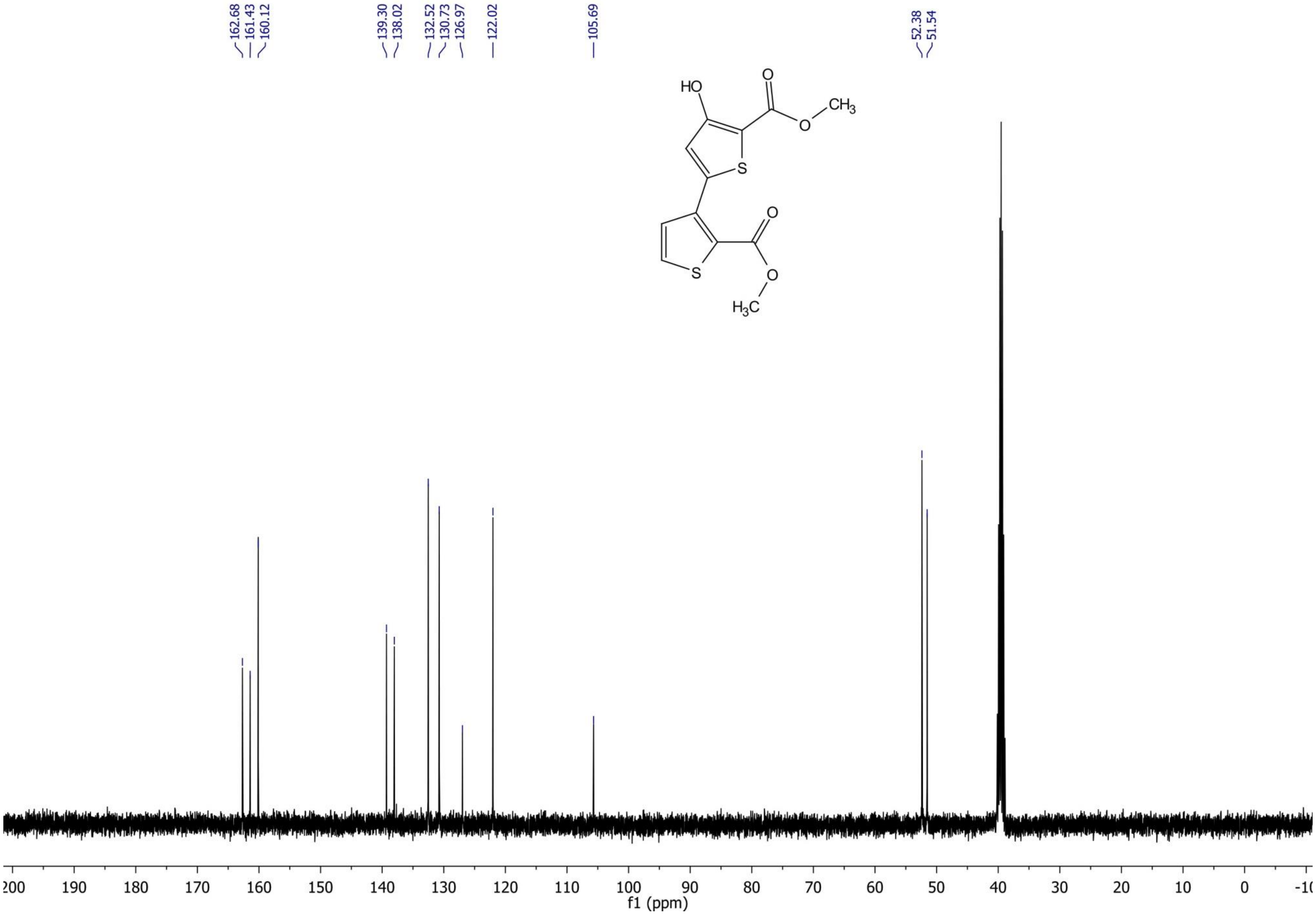

Methyl 4-hydroxy-5-(phenylcarbamoyl)-[2,3'-bithiophene]-2'-carboxylate 27b.

^{1}H NMR (400 MHz, [D$_6$]DMSO)

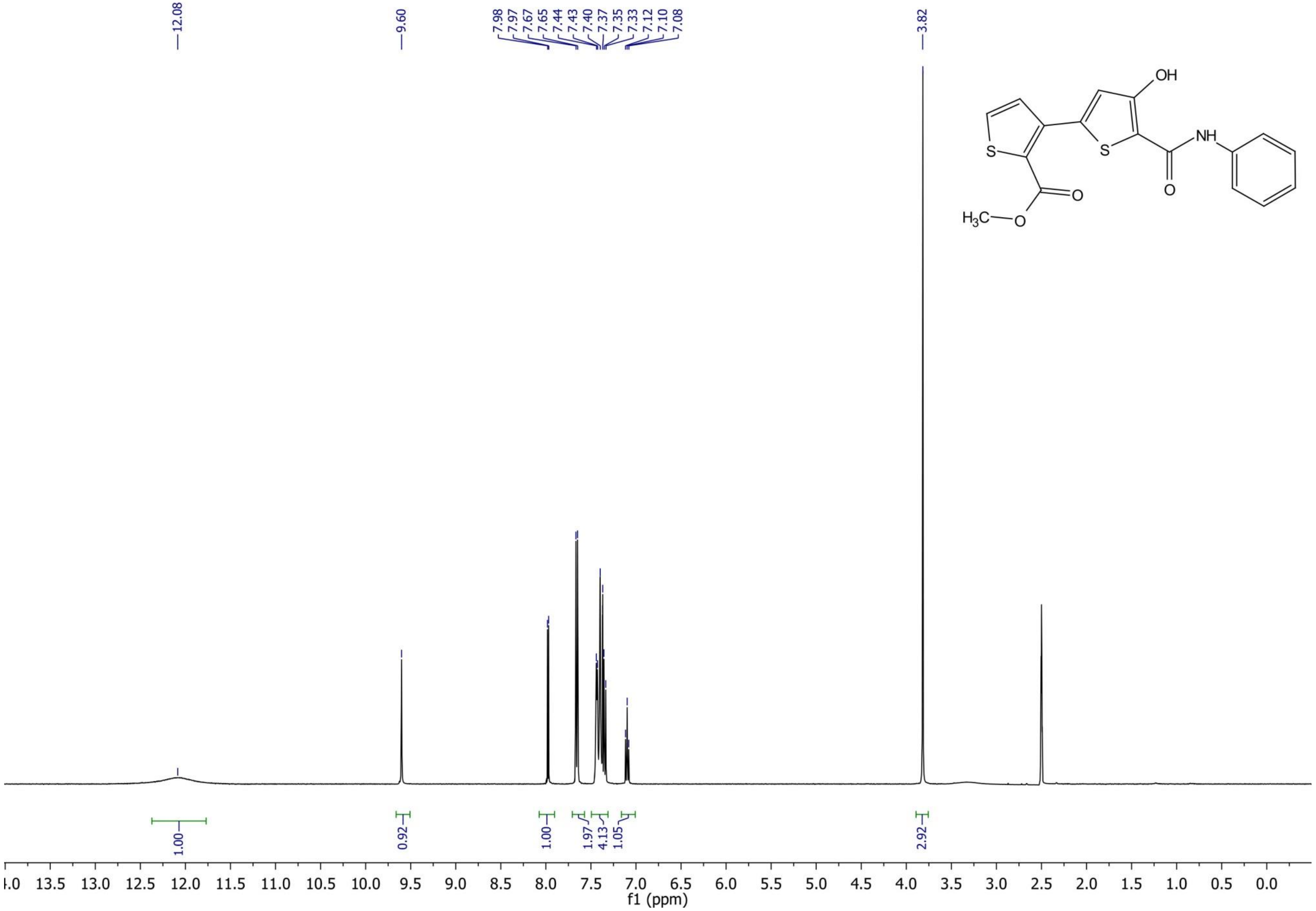

Methyl 4-hydroxy-5-(phenylcarbamoyl)-[2,3'-bithiophene]-2'-carboxylate 27b.

^{13}C NMR (101 MHz, [D$_6$]DMSO)

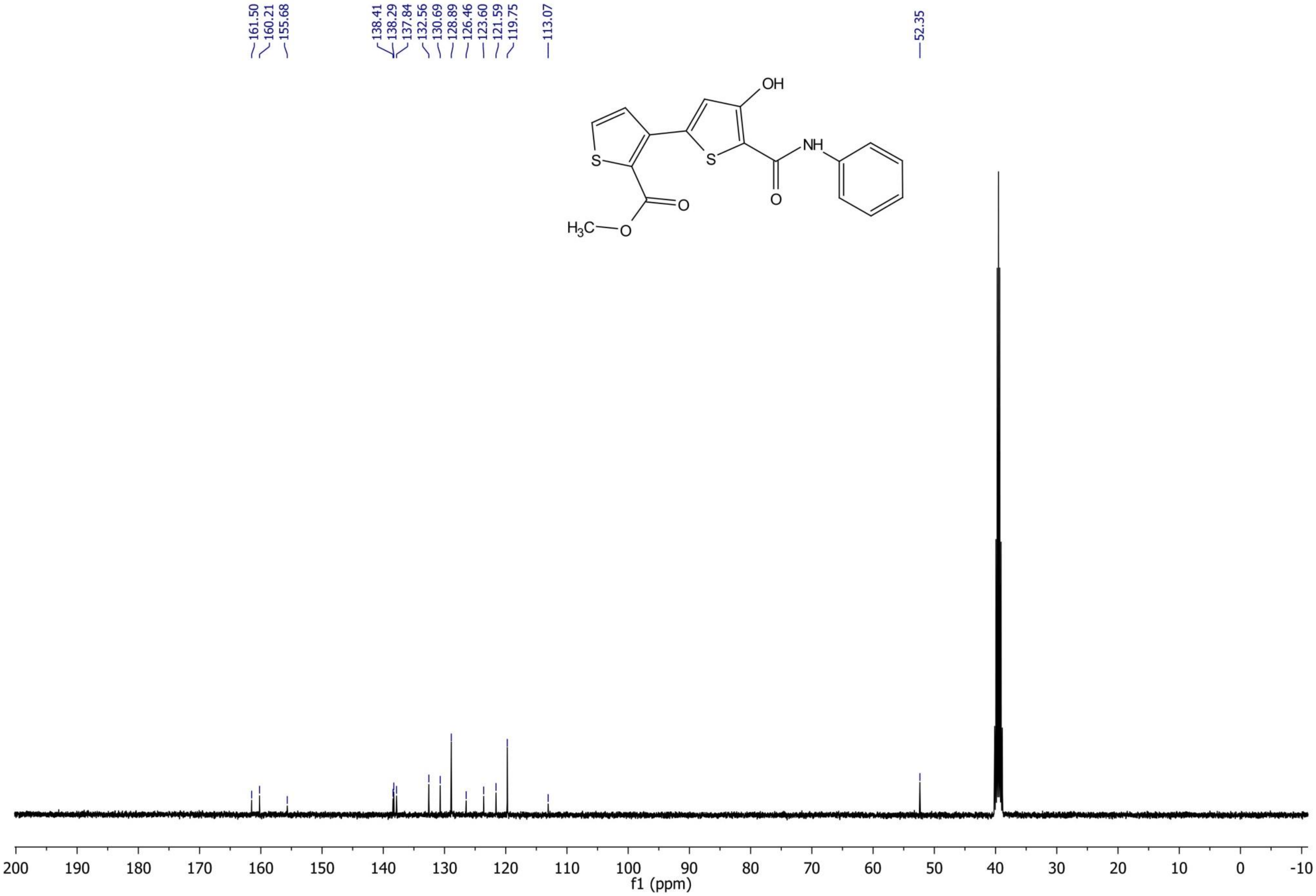

Methyl 5-acetyl-4-hydroxy-[2,3'-bithiophene]-2'-carboxylate 27c.

^{1}H NMR (400 MHz, $[D_6]DMSO$)

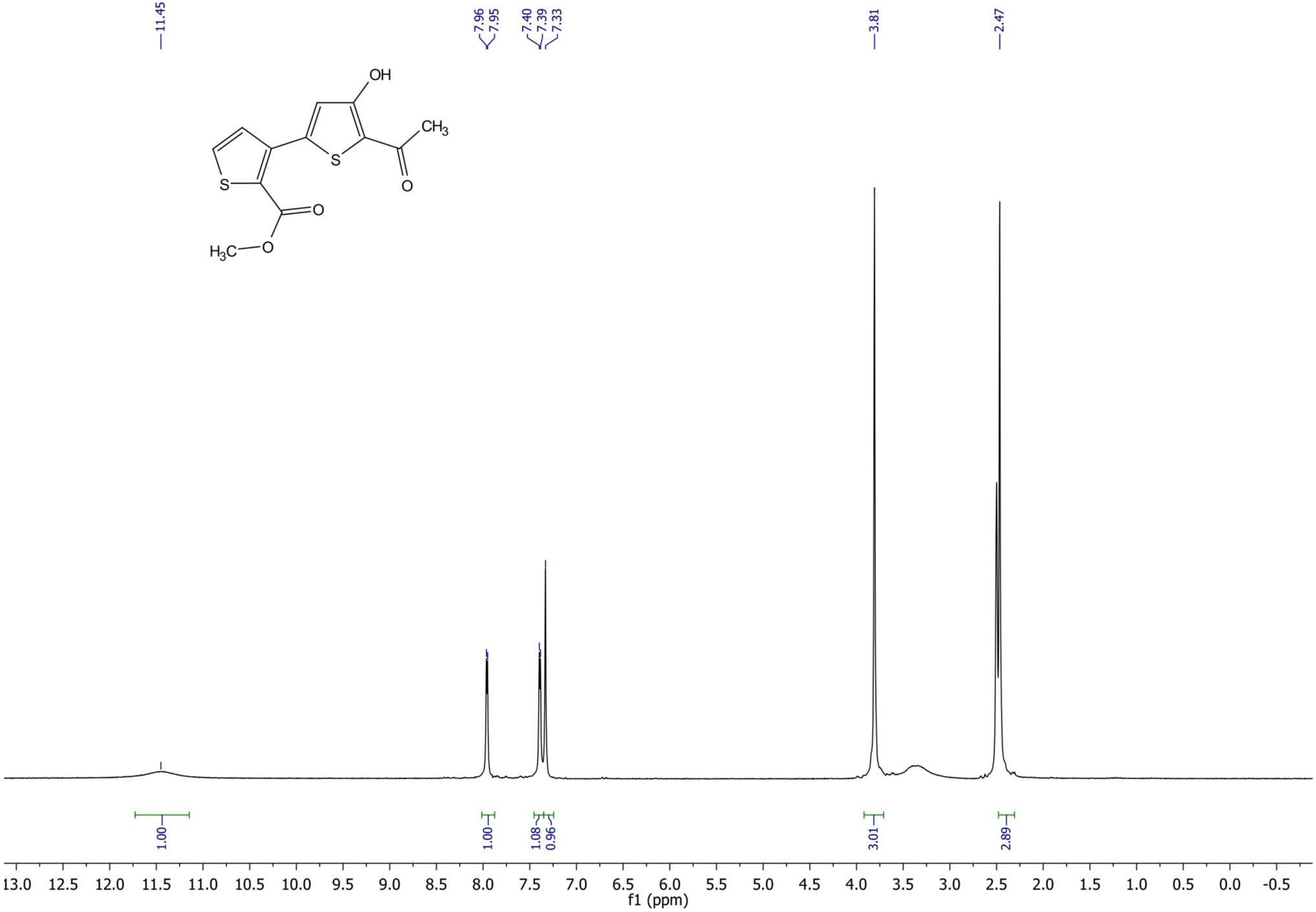

Methyl 5-acetyl-4-hydroxy-[2,3'-bithiophene]-2'-carboxylate 27c.

^{13}C NMR (101 MHz, $[D_6]DMSO$)

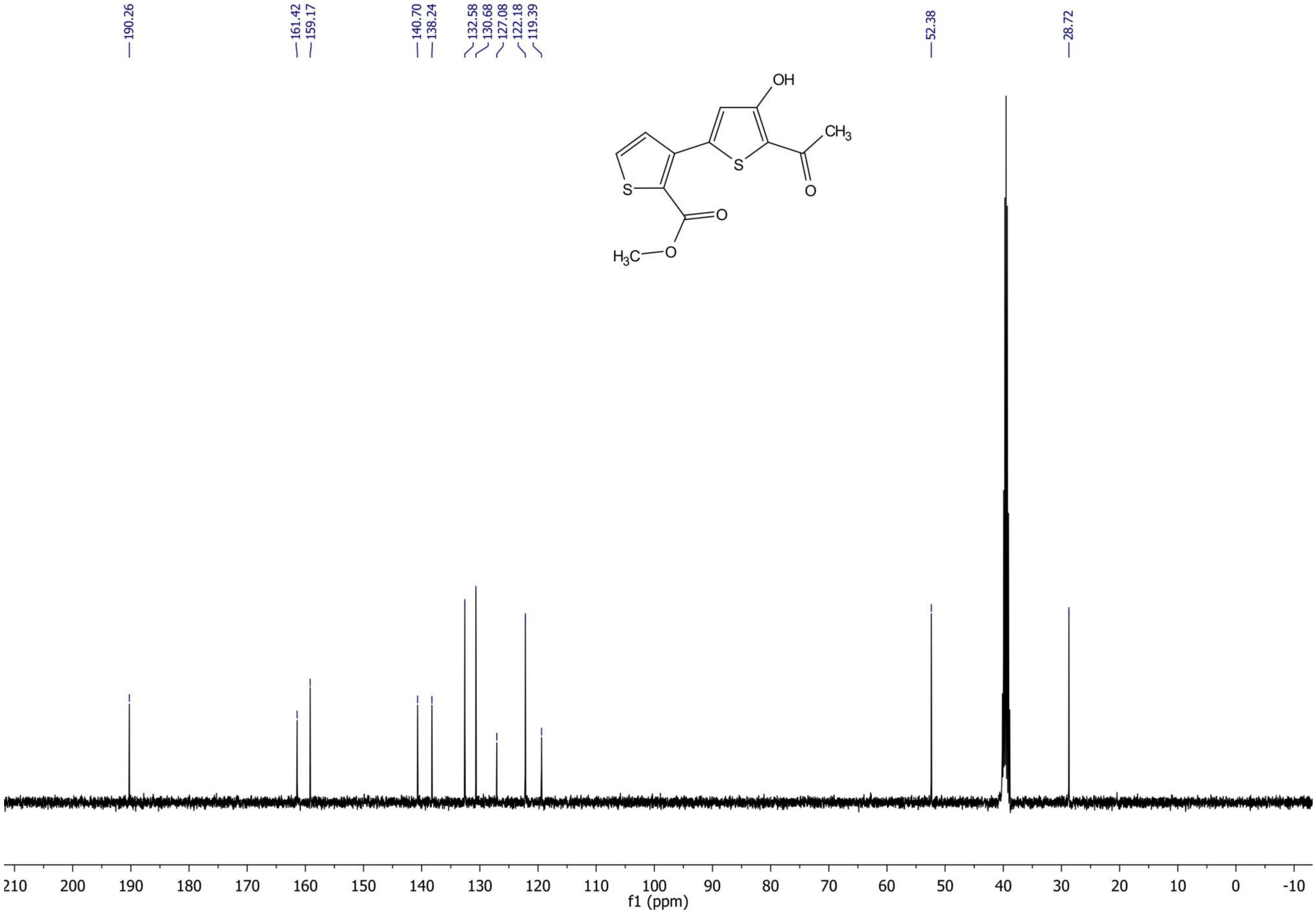

Supplement: Supplementary file 1 [file molecules-30-03758-s001.zip › molecules-3881044-supplementary.pdf]
